# Supplementary material for: Efficacy of Integrating a Novel 16-Gene Biomarker Panel and Intelligence Classifiers for Differential Diagnosis of Rheumatoid Arthritis and Osteoarthritis
Source: J Clin Med. 2019 Jan 6;8(1):50. doi: 10.3390/jcm8010050 (PMC6352223; doi:10.3390/jcm8010050)
Supplement: Supplementary file 1 [file jcm-08-00050-s001.pdf]

Article

Efficacy of integrating a novel 16-gene biomarker panel and intelligence classifiers for differential diagnosis of rheumatoid arthritis and osteoarthritis

Nguyen Phuoc Long[1], Seongoh Park[2], Nguyen Hoang Anh[1], Jung Eun Min[1], Sang Jun Yoon[1], Hyung Min Kim[1], Tran Diem Nghi[3], Dong Kyu Lim[1], Jeong Hill Park[1], Johan Lim[2], Sung Won Kwon[1,*]

1 College of Pharmacy and Research Institute of Pharmaceutical Sciences, Seoul National University, Seoul 08826, Republic of Korea.
2 Department of Statistics, Seoul National University, Seoul, 08826, Republic of Korea.
3 School of Medicine, Vietnam National University, Ho Chi Minh 700000, Vietnam.

* Correspondence: SWK (swkwon@snu.ac.kr); Tel.: +82-2-880-7844

Table S1. Microarray data sets employed in the current study

	Author	Data set	Year	Platform	Country	Participants		
						RA	OA	Normalcy
**RA versus OA**								
	Ungethuem *et al.* [1]	GSE1919	2004	Affymetrix U95A	Germany	3	3	
	Chang *et al.*	GSE39340	2012	Illumina V4.0	China	10	7	
	Lauwery *et al.* [2]	GSE36700	2012	Affymetrix 133 Plus 2.0	Belgium	7	5	
	Kinne *et al.* [3]	GSE55457	2014	Affymetrix 133A	Germany	13	10	
	Stiehl *et al.* [3]	GSE55584	2014	Affymetrix 133A	Germany	10	6	
	Häupl *et al.* [3]	GSE55235	2014	Affymetrix 133A	Germany	10	10	
**RA versus Normalcy**								
	Ungethuem *et al.* [1]	GSE1919	2004	Affymetrix U95A	Germany	3		5
	Kinne *et al.* [3]	GSE55457	2014	Affymetrix 133A	Germany	13		10
	Häupl *et al.* [3]	GSE55235	2014	Afffymetrix 133A	Germany	10		10
**OA versus Normalcy**								
	Ungethuem *et al.* [1]	GSE1919	2004	Affymetrix U95A	Germany		3	5
	Kinne *et al.* [3]	GSE55457	2014	Afffymetrix 133A	Germany		10	10
	Häupl *et al.* [3]	GSE55235	2014	Affymetrix 133A	Germany		10	10

Considering the biological and technical heterogeneity, we chose GSE1919, GSE39340, and GSE36700 for variable selection purpose as they were based on different microarray platforms and different sample properties. The established signature was then tested using three other datasets from the same study (GSE55457, GSE55584, and GSE55235). The number of samples for classification purpose is larger than that for variable selection purpose to ensure the training and the test set size.

Reference

1. Ungethuem U, Haeupl T, Witt H, Koczan D, Krenn V, Huber H, et al. Molecular signatures and new candidates to target the pathogenesis of rheumatoid arthritis. *Physiological genomics.* 2010;42a(4):267-282.

2. Nzeusseu Toukap A, Galant C, Theate I, Maudoux AL, Lories RJ, Houssiau FA, et al. Identification of distinct gene expression profiles in the synovium of patients with systemic lupus erythematosus. *Arthritis and rheumatism.* 2007;56(5):1579-1588.

3. Woetzel D, Huber R, Kupfer P, Pohlers D, Pfaff M, Driesch D, et al. Identification of rheumatoid arthritis and osteoarthritis patients by transcriptome-based rule set generation. *Arthritis research & therapy.* 2014;16(2):R84.

Table S2. GO and KEGG enriched pathways in RA versus OA.

Table S2.1. GO pathway enrichment in upregulated group of RA versus OA

Pathway ID	Pathway description	Observed gene count	False discovery rate
GO.0006955	Immune response	94	8.86E-50
GO.0050776	Regulation of immune response	71	1.44E-41
GO.0002376	Immune system process	101	2.64E-41
GO.0006952	Defense response	85	6.94E-39
GO.0002684	Positive regulation of immune system process	66	4.52E-36
GO.0002682	Regulation of immune system process	79	1.23E-34
GO.0050778	Positive regulation of immune response	54	7.78E-33
GO.0007166	Cell surface receptor signaling pathway	92	1.48E-32
GO.0002252	Immune effector process	42	9.42E-25
GO.0045087	Innate immune response	56	1.41E-23
GO.0051249	Regulation of lymphocyte activation	37	2.40E-23
GO.0002694	Regulation of leukocyte activation	39	3.29E-23
GO.0007165	Signal transduction	120	4.64E-23
GO.0048583	Regulation of response to stimulus	101	4.64E-23
GO.0050865	Regulation of cell activation	40	4.64E-23
GO.0002253	Activation of immune response	40	5.42E-23
GO.0002757	Immune response-activating signal transduction	38	8.76E-23
GO.0006950	Response to stress	102	8.76E-23
GO.0007159	Leukocyte cell-cell adhesion	33	1.10E-22
GO.0002764	Immune response-regulating signaling pathway	41	2.57E-22
GO.0002429	Immune response-activating cell surface receptor signaling pathway	33	3.64E-22
GO.0070486	Leukocyte aggregation	31	4.46E-22
GO.0046649	Lymphocyte activation	35	5.82E-22
GO.0016337	Single organismal cell-cell adhesion	39	6.73E-22
GO.0050867	Positive regulation of cell activation	32	6.73E-22
GO.0019221	Cytokine-mediated signaling pathway	37	9.61E-22
GO.0048584	Positive regulation of response to stimulus	74	1.61E-21
GO.0050851	Antigen receptor-mediated signaling pathway	24	1.85E-21
GO.0045321	Leukocyte activation	37	1.87E-21
GO.0002696	Positive regulation of leukocyte activation	31	2.55E-21
GO.0071345	Cellular response to cytokine stimulus	41	6.35E-21
GO.0044700	Single organism signaling	120	6.38E-21
GO.0002768	Immune response-regulating cell surface receptor signaling pathway	36	7.41E-21
GO.0034097	Response to cytokine	44	7.95E-21
GO.0051251	Positive regulation of lymphocyte activation	29	2.23E-20
GO.0071593	Lymphocyte aggregation	29	2.86E-20
GO.1903037	Regulation of leukocyte cell-cell adhesion	30	7.73E-20
GO.0007154	Cell communication	119	1.44E-19

GO.0001775	Cell activation	42	3.76E-19
GO.0050863	Regulation of t cell activation	29	4.27E-19
GO.0042110	T cell activation	27	4.06E-18
GO.0022409	Positive regulation of cell-cell adhesion	26	5.50E-18
GO.0030155	Regulation of cell adhesion	39	6.68E-18
GO.1903039	Positive regulation of leukocyte cell-cell adhesion	24	1.67E-17
GO.0009607	Response to biotic stimulus	42	2.04E-17
GO.0051707	Response to other organism	41	2.97E-17
GO.0050852	T cell receptor signaling pathway	19	3.71E-17
GO.0050896	Response to stimulus	136	6.21E-17
GO.0050870	Positive regulation of t cell activation	23	1.37E-16
GO.0022407	Regulation of cell-cell adhesion	30	1.47E-16
GO.0007155	Cell adhesion	47	2.45E-16
GO.0050900	Leukocyte migration	26	3.08E-16
GO.0001817	Regulation of cytokine production	35	7.81E-16
GO.0070663	Regulation of leukocyte proliferation	23	1.12E-15
GO.0045785	Positive regulation of cell adhesion	29	1.22E-15
GO.0072676	Lymphocyte migration	13	2.64E-15
GO.0031347	Regulation of defense response	40	3.35E-15
GO.0051716	Cellular response to stimulus	121	4.38E-15
GO.0050670	Regulation of lymphocyte proliferation	22	5.34E-15
GO.0006954	Inflammatory response	31	9.22E-15
GO.0030098	Lymphocyte differentiation	23	9.29E-15
GO.0002697	Regulation of immune effector process	30	1.08E-14
GO.1902105	Regulation of leukocyte differentiation	24	2.21E-14
GO.0048518	Positive regulation of biological process	108	2.55E-14
GO.0002250	Adaptive immune response	20	1.44E-13
GO.0045580	Regulation of t cell differentiation	17	2.57E-13
GO.0098542	Defense response to other organism	27	3.35E-13
GO.0001819	Positive regulation of cytokine production	27	3.83E-13
GO.0030217	T cell differentiation	18	4.24E-13
GO.0042129	Regulation of t cell proliferation	18	4.87E-13
GO.0050671	Positive regulation of lymphocyte proliferation	17	5.70E-13
GO.1902107	Positive regulation of leukocyte differentiation	18	8.46E-13
GO.0045619	Regulation of lymphocyte differentiation	18	1.11E-12
GO.1903706	Regulation of hemopoiesis	25	2.03E-12
GO.0030595	Leukocyte chemotaxis	16	2.56E-12
GO.0002703	Regulation of leukocyte mediated immunity	18	3.18E-12
GO.0002366	Leukocyte activation involved in immune response	17	3.96E-12
GO.0045088	Regulation of innate immune response	26	4.21E-12
GO.0009605	Response to external stimulus	58	4.59E-12
GO.1902531	Regulation of intracellular signal transduction	49	5.93E-12
GO.0002819	Regulation of adaptive immune response	16	6.38E-12
GO.0009967	Positive regulation of signal transduction	47	1.12E-11
GO.0002521	Leukocyte differentiation	23	1.39E-11

GO.1902533	Positive regulation of intracellular signal transduction	37	1.39E-11
GO.0009615	Response to virus	21	1.59E-11
GO.0048522	Positive regulation of cellular process	93	2.36E-11
GO.0009966	Regulation of signal transduction	65	2.83E-11
GO.0045621	Positive regulation of lymphocyte differentiation	14	2.83E-11
GO.0010033	Response to organic substance	66	3.06E-11
GO.0051704	Multi-organism process	62	3.16E-11
GO.0060326	Cell chemotaxis	18	3.84E-11
GO.0046631	Alpha-beta t cell activation	12	5.54E-11
GO.0035556	Intracellular signal transduction	55	7.19E-11
GO.0023056	Positive regulation of signaling	48	9.42E-11
GO.0009617	Response to bacterium	27	1.07E-10
GO.0002285	Lymphocyte activation involved in immune response	14	1.29E-10
GO.0065009	Regulation of molecular function	68	1.44E-10
GO.0042102	Positive regulation of t cell proliferation	13	2.51E-10
GO.0002460	Adaptive immune response based on somatic recombination of immune receptors built from immunoglobulin superfamily domains	15	3.19E-10
GO.0002822	Regulation of adaptive immune response based on somatic recombination of immune receptors built from immunoglobulin superfamily domains	14	4.42E-10
GO.0023051	Regulation of signaling	67	5.81E-10
GO.0080134	Regulation of response to stress	46	6.53E-10
GO.0002520	Immune system development	29	1.10E-09
GO.0045582	Positive regulation of t cell differentiation	12	1.10E-09
GO.0070887	Cellular response to chemical stimulus	60	1.19E-09
GO.0002683	Negative regulation of immune system process	23	1.23E-09
GO.0048247	Lymphocyte chemotaxis	8	1.23E-09
GO.0010647	Positive regulation of cell communication	48	1.81E-09
GO.0032496	Response to lipopolysaccharide	20	3.11E-09
GO.0007596	Blood coagulation	27	3.82E-09
GO.0006968	Cellular defense response	11	4.58E-09
GO.0010646	Regulation of cell communication	67	5.20E-09
GO.0050853	B cell receptor signaling pathway	9	5.20E-09
GO.0046632	Alpha-beta t cell differentiation	10	6.13E-09
GO.0050790	Regulation of catalytic activity	57	7.60E-09
GO.0002443	Leukocyte mediated immunity	15	9.01E-09
GO.0048534	Hematopoietic or lymphoid organ development	27	1.01E-08
GO.0071310	Cellular response to organic substance	51	1.13E-08
GO.0016477	Cell migration	31	1.21E-08
GO.0009893	Positive regulation of metabolic process	75	1.30E-08
GO.0060333	Interferon-gamma-mediated signaling pathway	11	1.32E-08
GO.0034341	Response to interferon-gamma	13	2.37E-08

GO.0042127	Regulation of cell proliferation	44	2.37E-08
GO.0072678	T cell migration	7	2.47E-08
GO.0002449	Lymphocyte mediated immunity	13	2.62E-08
GO.0050777	Negative regulation of immune response	13	3.29E-08
GO.0030097	Hemopoiesis	25	3.63E-08
GO.0071346	Cellular response to interferon-gamma	12	3.63E-08
GO.0065008	Regulation of biological quality	67	3.86E-08
GO.0002286	T cell activation involved in immune response	10	5.77E-08
GO.0051240	Positive regulation of multicellular organismal process	41	6.32E-08
GO.0032879	Regulation of localization	56	6.87E-08
GO.0070098	Chemokine-mediated signaling pathway	9	6.99E-08
GO.0006915	Apoptotic process	36	7.45E-08
GO.0031349	Positive regulation of defense response	21	7.62E-08
GO.0040011	Locomotion	38	8.06E-08
GO.0060341	Regulation of cellular localization	38	8.22E-08
GO.0002706	Regulation of lymphocyte mediated immunity	12	8.37E-08
GO.0051674	Localization of cell	31	8.70E-08
GO.0006935	Chemotaxis	27	8.81E-08
GO.2000406	Positive regulation of t cell migration	7	9.32E-08
GO.0048585	Negative regulation of response to stimulus	40	9.73E-08
GO.0002687	Positive regulation of leukocyte migration	12	1.02E-07
GO.0030335	Positive regulation of cell migration	20	1.02E-07
GO.0046634	Regulation of alpha-beta t cell activation	10	1.07E-07
GO.0042060	Wound healing	28	1.19E-07
GO.0050864	Regulation of b cell activation	12	1.27E-07
GO.0043067	Regulation of programmed cell death	41	1.37E-07
GO.0044093	Positive regulation of molecular function	46	1.38E-07
GO.0045058	T cell selection	8	1.38E-07
GO.0042113	B cell activation	13	1.42E-07
GO.0050871	Positive regulation of b cell activation	10	1.44E-07
GO.0050707	Regulation of cytokine secretion	13	1.70E-07
GO.0007162	Negative regulation of cell adhesion	16	1.75E-07
GO.0051239	Regulation of multicellular organismal process	56	1.79E-07
GO.0051607	Defense response to virus	14	1.82E-07
GO.0032844	Regulation of homeostatic process	20	2.13E-07
GO.0009611	Response to wounding	29	2.22E-07
GO.0002685	Regulation of leukocyte migration	13	2.86E-07
GO.0012501	Programmed cell death	35	2.86E-07
GO.2000401	Regulation of lymphocyte migration	8	2.86E-07
GO.0060337	Type i interferon signaling pathway	10	2.94E-07
GO.0071357	Cellular response to type i interferon	10	2.94E-07
GO.0042981	Regulation of apoptotic process	40	3.36E-07
GO.0032663	Regulation of interleukin-2 production	9	3.61E-07
GO.0051223	Regulation of protein transport	28	4.04E-07
GO.0042035	Regulation of cytokine biosynthetic process	11	4.34E-07

GO.0050854	Regulation of antigen receptor-mediated signaling pathway	8	5.54E-07
GO.0070201	Regulation of establishment of protein localization	29	6.02E-07
GO.0048519	Negative regulation of biological process	80	7.20E-07
GO.0051049	Regulation of transport	45	7.40E-07
GO.0006928	Movement of cell or subcellular component	38	1.06E-06
GO.0071216	Cellular response to biotic stimulus	13	1.06E-06
GO.0042221	Response to chemical	73	1.10E-06
GO.0071353	Cellular response to interleukin-4	7	1.19E-06
GO.0051247	Positive regulation of protein metabolic process	39	1.27E-06
GO.0071222	Cellular response to lipopolysaccharide	12	1.37E-06
GO.0050715	Positive regulation of cytokine secretion	10	1.39E-06
GO.0050727	Regulation of inflammatory response	16	1.39E-06
GO.2000107	Negative regulation of leukocyte apoptotic process	8	1.50E-06
GO.2000026	Regulation of multicellular organismal development	41	1.75E-06
GO.0072503	Cellular divalent inorganic cation homeostasis	17	1.93E-06
GO.0031325	Positive regulation of cellular metabolic process	60	2.11E-06
GO.0065007	Biological regulation	139	2.55E-06
GO.0038093	Fc receptor signaling pathway	15	2.62E-06
GO.0042592	Homeostatic process	37	2.62E-06
GO.0051336	Regulation of hydrolase activity	36	2.87E-06
GO.0050793	Regulation of developmental process	49	3.07E-06
GO.0010604	Positive regulation of macromolecule metabolic process	57	3.22E-06
GO.0006875	Cellular metal ion homeostasis	19	3.52E-06
GO.0048523	Negative regulation of cellular process	74	3.53E-06
GO.0051050	Positive regulation of transport	29	3.59E-06
GO.0001816	Cytokine production	11	3.88E-06
GO.0031341	Regulation of cell killing	8	4.19E-06
GO.0032845	Negative regulation of homeostatic process	10	4.19E-06
GO.0002699	Positive regulation of immune effector process	12	4.86E-06
GO.0010819	Regulation of t cell chemotaxis	5	5.06E-06
GO.0033993	Response to lipid	27	5.49E-06
GO.0002274	Myeloid leukocyte activation	10	5.62E-06
GO.0006874	Cellular calcium ion homeostasis	16	5.71E-06
GO.0046640	Regulation of alpha-beta t cell proliferation	6	5.99E-06
GO.0045089	Positive regulation of innate immune response	16	6.59E-06
GO.0043069	Negative regulation of programmed cell death	28	6.65E-06
GO.0040012	Regulation of locomotion	25	7.48E-06
GO.0050789	Regulation of biological process	134	7.51E-06
GO.0050714	Positive regulation of protein secretion	13	7.66E-06
GO.0002293	Alpha-beta t cell differentiation involved in immune response	6	7.83E-06
GO.0002456	T cell mediated immunity	6	7.83E-06

GO.0019835	Cytolysis	6	7.83E-06
GO.1903034	Regulation of response to wounding	18	7.95E-06
GO.0050708	Regulation of protein secretion	18	9.73E-06
GO.0051046	Regulation of secretion	24	9.90E-06
GO.0032673	Regulation of interleukin-4 production	6	1.02E-05
GO.0002690	Positive regulation of leukocyte chemotaxis	9	1.07E-05
GO.0032101	Regulation of response to external stimulus	28	1.12E-05
GO.0042108	Positive regulation of cytokine biosynthetic process	8	1.30E-05
GO.0002705	Positive regulation of leukocyte mediated immunity	9	1.32E-05
GO.2000106	Regulation of leukocyte apoptotic process	9	1.32E-05
GO.0055082	Cellular chemical homeostasis	21	1.43E-05
GO.0032386	Regulation of intracellular transport	22	1.48E-05
GO.0043085	Positive regulation of catalytic activity	37	1.60E-05
GO.0051246	Regulation of protein metabolic process	52	1.60E-05
GO.0043066	Negative regulation of apoptotic process	27	1.85E-05
GO.0050857	Positive regulation of antigen receptor-mediated signaling pathway	5	1.86E-05
GO.0001910	Regulation of leukocyte mediated cytotoxicity	7	2.04E-05
GO.0002824	Positive regulation of adaptive immune response based on somatic recombination of immune receptors built from immunoglobulin superfamily domains	8	2.15E-05
GO.0032649	Regulation of interferon-gamma production	9	2.20E-05
GO.0001818	Negative regulation of cytokine production	13	2.37E-05
GO.2000145	Regulation of cell motility	23	2.41E-05
GO.0033081	Regulation of t cell differentiation in thymus	6	2.66E-05
GO.0046641	Positive regulation of alpha-beta t cell proliferation	5	2.67E-05
GO.0050801	Ion homeostasis	21	2.67E-05
GO.0046637	Regulation of alpha-beta t cell differentiation	7	2.76E-05
GO.1903530	Regulation of secretion by cell	22	2.99E-05
GO.0002218	Activation of innate immune response	14	3.19E-05
GO.0002637	Regulation of immunoglobulin production	7	3.20E-05
GO.0030334	Regulation of cell migration	22	3.21E-05
GO.0050794	Regulation of cellular process	128	3.55E-05
GO.0002407	Dendritic cell chemotaxis	5	3.70E-05
GO.0046635	Positive regulation of alpha-beta t cell activation	7	3.70E-05
GO.0032479	Regulation of type i interferon production	10	3.85E-05
GO.0051222	Positive regulation of protein transport	18	3.85E-05
GO.0002639	Positive regulation of immunoglobulin production	6	3.94E-05
GO.0032735	Positive regulation of interleukin-12 production	6	3.94E-05
GO.0050850	Positive regulation of calcium-mediated signaling	6	3.94E-05

GO.0032270	Positive regulation of cellular protein metabolic process	34	4.01E-05
GO.0050848	Regulation of calcium-mediated signaling	7	4.90E-05
GO.0098771	Inorganic ion homeostasis	20	4.93E-05
GO.0070233	Negative regulation of t cell apoptotic process	5	5.02E-05
GO.0046425	Regulation of jak-stat cascade	9	5.36E-05
GO.0002666	Positive regulation of t cell tolerance induction	4	5.38E-05
GO.0002693	Positive regulation of cellular extravasation	4	5.38E-05
GO.0032388	Positive regulation of intracellular transport	16	5.46E-05
GO.0045124	Regulation of bone resorption	6	5.82E-05
GO.0045937	Positive regulation of phosphate metabolic process	28	6.16E-05
GO.0048878	Chemical homeostasis	26	6.16E-05
GO.1901700	Response to oxygen-containing compound	34	6.16E-05
GO.0007204	Positive regulation of cytosolic calcium ion concentration	11	6.53E-05
GO.0032753	Positive regulation of interleukin-4 production	5	6.62E-05
GO.0042832	Defense response to protozoan	5	6.62E-05
GO.0051209	Release of sequestered calcium ion into cytosol	6	6.88E-05
GO.0008284	Positive regulation of cell proliferation	25	7.21E-05
GO.0043122	Regulation of i-kappab kinase/nf-kappab signaling	13	8.40E-05
GO.1903649	Regulation of cytoplasmic transport	18	8.52E-05
GO.0010818	T cell chemotaxis	4	9.09E-05
GO.0038096	Fc-gamma receptor signaling pathway involved in phagocytosis	8	9.34E-05
GO.2000514	Regulation of cd4-positive		
GO.0030168	Platelet activation	13	0.000104
GO.0042093	T-helper cell differentiation	5	0.000111
GO.0001932	Regulation of protein phosphorylation	31	0.000114
GO.0002758	Innate immune response-activating signal transduction	13	0.000119
GO.0051345	Positive regulation of hydrolase activity	25	0.000128
GO.0019220	Regulation of phosphate metabolic process	36	0.00013
GO.0044763	Single-organism cellular process	136	0.00013
GO.0030890	Positive regulation of b cell proliferation	6	0.000132
GO.0002695	Negative regulation of leukocyte activation	10	0.000136
GO.0010820	Positive regulation of t cell chemotaxis	4	0.000139
GO.0034103	Regulation of tissue remodeling	7	0.000143
GO.0051047	Positive regulation of secretion	15	0.000156
GO.0008283	Cell proliferation	22	0.000158
GO.0042325	Regulation of phosphorylation	32	0.000164
GO.0046651	Lymphocyte proliferation	8	0.000171
GO.0044092	Negative regulation of molecular function	29	0.000183
GO.0044403	Symbiosis		
GO.0051241	Negative regulation of multicellular organismal process	27	0.000191

GO.0043901	Negative regulation of multi-organism process	10	0.000192
GO.0031295	T cell costimulation	7	0.000196
GO.0042509	Regulation of tyrosine phosphorylation of stat protein	7	0.000196
GO.0050868	Negative regulation of t cell activation	8	0.000199
GO.2000021	Regulation of ion homeostasis	11	0.000199
GO.0002704	Negative regulation of leukocyte mediated immunity	6	0.0002
GO.0038083	Peptidyl-tyrosine autophosphorylation	6	0.0002
GO.1903532	Positive regulation of secretion by cell	14	0.000202
GO.0007169	Transmembrane receptor protein tyrosine kinase signaling pathway	22	0.000246
GO.0002688	Regulation of leukocyte chemotaxis	8	0.000255
GO.0031334	Positive regulation of protein complex assembly	10	0.000255
GO.0051250	Negative regulation of lymphocyte activation	9	0.000261
GO.0002698	Negative regulation of immune effector process	8	0.000297
GO.0032268	Regulation of cellular protein metabolic process	46	0.000297
GO.0033089	Positive regulation of t cell differentiation in thymus	4	0.000297
GO.1903305	Regulation of regulated secretory pathway	6	0.000302
GO.0002828	Regulation of type 2 immune response	5	0.000316
GO.0031399	Regulation of protein modification process	36	0.000351
GO.0046427	Positive regulation of jak-stat cascade	7	0.000353
GO.0016032	Viral process	22	0.00036
GO.1903555	Regulation of tumor necrosis factor superfamily cytokine production	8	0.000371
GO.0042742	Defense response to bacterium	11	0.000372
GO.0042327	Positive regulation of phosphorylation	24	0.000387
GO.0046777	Protein autophosphorylation	11	0.000387
GO.0050731	Positive regulation of peptidyl-tyrosine phosphorylation	10	0.000387
GO.0051092	Positive regulation of nf-kappab transcription factor activity	9	0.000399
GO.0045595	Regulation of cell differentiation	34	0.000427
GO.0002707	Negative regulation of lymphocyte mediated immunity	5	0.000449
GO.0050856	Regulation of t cell receptor signaling pathway	5	0.000449
GO.0043900	Regulation of multi-organism process	17	0.000467
GO.0034113	Heterotypic cell-cell adhesion	5	0.000537
GO.0006909	Phagocytosis	10	0.000557
GO.0006959	Humoral immune response	9	0.000571
GO.0031348	Negative regulation of defense response	9	0.000571
GO.0001934	Positive regulation of protein phosphorylation	23	0.000585
GO.0051094	Positive regulation of developmental process	28	0.000606
GO.0052548	Regulation of endopeptidase activity	15	0.00061
GO.0019222	Regulation of metabolic process	94	0.000614
GO.0045597	Positive regulation of cell differentiation	23	0.000617

GO.0002823	Negative regulation of adaptive immune response based on somatic recombination of immune receptors built from immunoglobulin superfamily domains	5	0.000622
GO.0051281	Positive regulation of release of sequestered calcium ion into cytosol	5	0.000622
GO.0022408	Negative regulation of cell-cell adhesion	9	0.000633
GO.0009968	Negative regulation of signal transduction	27	0.000689
GO.0032703	Negative regulation of interleukin-2 production	4	0.000713
GO.0071621	Granulocyte chemotaxis	6	0.000759
GO.1903557	Positive regulation of tumor necrosis factor superfamily cytokine production	6	0.000759
GO.0097529	Myeloid leukocyte migration	7	0.000905
GO.0043086	Negative regulation of catalytic activity	23	0.000947
GO.0045824	Negative regulation of innate immune response	5	0.001
GO.1903651	Positive regulation of cytoplasmic transport	12	0.00101
GO.0007259	Jak-stat cascade	6	0.00104
GO.0032729	Positive regulation of interferon-gamma production	6	0.00104
GO.0042531	Positive regulation of tyrosine phosphorylation of stat protein	6	0.00104
GO.0070228	Regulation of lymphocyte apoptotic process	6	0.00104
GO.0050920	Regulation of chemotaxis	9	0.0011
GO.0051346	Negative regulation of hydrolase activity	15	0.00115
GO.0045061	Thymic t cell selection	4	0.00117
GO.0001959	Regulation of cytokine-mediated signaling pathway	8	0.00131
GO.0032743	Positive regulation of interleukin-2 production	5	0.00131
GO.0070232	Regulation of t cell apoptotic process	5	0.00131
GO.2000116	Regulation of cysteine-type endopeptidase activity	11	0.00132
GO.0048872	Homeostasis of number of cells	10	0.00134
GO.0023057	Negative regulation of signaling	28	0.00138
GO.0048535	Lymph node development	4	0.00146
GO.0031401	Positive regulation of protein modification process	26	0.00149
GO.0033003	Regulation of mast cell activation	5	0.00149
GO.0032846	Positive regulation of homeostatic process	9	0.00155
GO.0051090	Regulation of sequence-specific dna binding transcription factor activity	14	0.00155
GO.0071396	Cellular response to lipid	14	0.00155
GO.0010648	Negative regulation of cell communication	28	0.00156
GO.0045859	Regulation of protein kinase activity	20	0.00157
GO.0022603	Regulation of anatomical structure morphogenesis	23	0.00158
GO.0045084	Positive regulation of interleukin-12 biosynthetic process	3	0.00158

GO.0050862	Positive regulation of t cell receptor signaling pathway	3	0.00158
GO.0034612	Response to tumor necrosis factor	8	0.00162
GO.0007167	Enzyme linked receptor protein signaling pathway	24	0.00163
GO.0042119	Neutrophil activation	4	0.00177
GO.0043368	Positive t cell selection	4	0.00177
GO.0031324	Negative regulation of cellular metabolic process	43	0.00183
GO.0050729	Positive regulation of inflammatory response	7	0.00184
GO.0002886	Regulation of myeloid leukocyte mediated immunity	5	0.00188
GO.0043300	Regulation of leukocyte degranulation	5	0.00188
GO.0046638	Positive regulation of alpha-beta t cell differentiation	5	0.00188
GO.2000351	Regulation of endothelial cell apoptotic process	5	0.00188
GO.0010951	Negative regulation of endopeptidase activity	11	0.0021
GO.0032680	Regulation of tumor necrosis factor production	7	0.0021
GO.0043154	Negative regulation of cysteine-type endopeptidase activity involved in apoptotic process	7	0.0021
GO.0010557	Positive regulation of macromolecule biosynthetic process	34	0.00212
GO.0042100	B cell proliferation	5	0.00212
GO.0002825	Regulation of t-helper 1 type immune response	4	0.00213
GO.0043087	Regulation of gtpase activity	17	0.00218
GO.0050795	Regulation of behavior	10	0.00225
GO.0002708	Positive regulation of lymphocyte mediated immunity	6	0.0023
GO.0043547	Positive regulation of gtpase activity	16	0.00234
GO.0009891	Positive regulation of biosynthetic process	36	0.00237
GO.0033628	Regulation of cell adhesion mediated by integrin	5	0.00237
GO.0033632	Regulation of cell-cell adhesion mediated by integrin	3	0.00237
GO.0034121	Regulation of toll-like receptor signaling pathway	5	0.00237
GO.0051130	Positive regulation of cellular component organization	27	0.00256
GO.0002889	Regulation of immunoglobulin mediated immune response	5	0.00265
GO.0019882	Antigen processing and presentation	10	0.00284
GO.0071356	Cellular response to tumor necrosis factor	7	0.00287
GO.0002709	Regulation of t cell mediated immunity	5	0.00296
GO.0032648	Regulation of interferon-beta production	5	0.00296
GO.0002573	Myeloid leukocyte differentiation	7	0.00302
GO.0010605	Negative regulation of macromolecule metabolic process	42	0.00302

GO.0043254	Regulation of protein complex assembly	12	0.00302
GO.0044089	Positive regulation of cellular component biogenesis	13	0.00302
GO.1902624	Positive regulation of neutrophil migration	4	0.00302
GO.0051279	Regulation of release of sequestered calcium ion into cytosol	6	0.00313
GO.0070664	Negative regulation of leukocyte proliferation	6	0.00313
GO.0006897	Endocytosis	16	0.00318
GO.0032480	Negative regulation of type i interferon production	5	0.00326
GO.0001911	Negative regulation of leukocyte mediated cytotoxicity	3	0.00333
GO.0002313	Mature b cell differentiation involved in immune response	3	0.00333
GO.0002676	Regulation of chronic inflammatory response	3	0.00333
GO.0031328	Positive regulation of cellular biosynthetic process	35	0.00333
GO.0045779	Negative regulation of bone resorption	3	0.00333
GO.0048302	Regulation of isotype switching to igg isotypes	3	0.00333
GO.0042267	Natural killer cell mediated cytotoxicity	4	0.0035
GO.1903307	Positive regulation of regulated secretory pathway	4	0.0035
GO.0001866	Nk t cell proliferation	2	0.00353
GO.0002457	T cell antigen processing and presentation	2	0.00353
GO.0003366	Cell-matrix adhesion involved in ameboidal cell migration	2	0.00353
GO.0038111	Interleukin-7-mediated signaling pathway	2	0.00353
GO.0043366	Beta selection	2	0.00353
GO.0051924	Regulation of calcium ion transport	10	0.00353
GO.0098761	Cellular response to interleukin-7	2	0.00353
GO.2000309	Positive regulation of tumor necrosis factor (ligand) superfamily member 11 production	2	0.00353
GO.2000412	Positive regulation of thymocyte migration	2	0.00353
GO.2000473	Positive regulation of hematopoietic stem cell migration	2	0.00353
GO.2000564	Regulation of cd8-positive		
GO.0038095	Fc-epsilon receptor signaling pathway	9	0.00356
GO.0006816	Calcium ion transport	10	0.00363
GO.0010628	Positive regulation of gene expression	34	0.00409
GO.0009892	Negative regulation of metabolic process	45	0.00438
GO.0045589	Regulation of regulatory t cell differentiation	3	0.00449
GO.2000345	Regulation of hepatocyte proliferation	3	0.00449
GO.0034105	Positive regulation of tissue remodeling	4	0.00469
GO.0043123	Positive regulation of i-kappab kinase/nf-kappab signaling	9	0.00478
GO.0051091	Positive regulation of sequence-specific dna binding transcription factor activity	10	0.00487

GO.0051179	Localization	67	0.00487
GO.0030162	Regulation of proteolysis	20	0.00515
GO.0044707	Single-multicellular organism process	80	0.00541
GO.2000516	Positive regulation of cd4-positive		
GO.0051928	Positive regulation of calcium ion transport	7	0.00551
GO.0032760	Positive regulation of tumor necrosis factor production	5	0.00571
GO.0032103	Positive regulation of response to external stimulus	13	0.00595
GO.0035458	Cellular response to interferon-beta	3	0.00595
GO.0045060	Negative thymic t cell selection	3	0.00595
GO.0030833	Regulation of actin filament polymerization	7	0.00611
GO.0007229	Integrin-mediated signaling pathway	6	0.00617
GO.0043299	Leukocyte degranulation	4	0.00617
GO.0060142	Regulation of syncytium formation by plasma membrane fusion	4	0.00617
GO.0001906	Cell killing	5	0.00677
GO.0042130	Negative regulation of t cell proliferation	5	0.00677
GO.0032501	Multicellular organismal process	82	0.00693
GO.0050688	Regulation of defense response to virus	9	0.00697
GO.0030101	Natural killer cell activation	5	0.0074
GO.0002761	Regulation of myeloid leukocyte differentiation	7	0.00751
GO.0002532	Production of molecular mediator involved in inflammatory response	3	0.00766
GO.0043304	Regulation of mast cell degranulation	4	0.00804
GO.0045581	Negative regulation of t cell differentiation	4	0.00804
GO.2001233	Regulation of apoptotic signaling pathway	13	0.00816
GO.0034138	Toll-like receptor 3 signaling pathway	6	0.00848
GO.1902106	Negative regulation of leukocyte differentiation	6	0.00848
GO.0051495	Positive regulation of cytoskeleton organization	8	0.00867
GO.0051153	Regulation of striated muscle cell differentiation	6	0.00903
GO.0043370	Regulation of cd4-positive		
GO.0048731	System development	57	0.00924
GO.0030099	Myeloid cell differentiation	9	0.00929
GO.0002291	T cell activation via t cell receptor contact with antigen bound to mhc molecule on antigen presenting cell	2	0.00948
GO.0006468	Protein phosphorylation	20	0.00948
GO.0034124	Regulation of myd88-dependent toll-like receptor signaling pathway	2	0.00948
GO.0043950	Positive regulation of camp-mediated signaling	3	0.00948
GO.0045086	Positive regulation of interleukin-2 biosynthetic process	3	0.00948
GO.0045780	Positive regulation of bone resorption	3	0.00948
GO.0072540	T-helper 17 cell lineage commitment	2	0.00948
GO.0090026	Positive regulation of monocyte chemotaxis	3	0.00948
GO.0030183	B cell differentiation	6	0.00999

GO.0002312	B cell activation involved in immune response	4	0.01
GO.0046627	Negative regulation of insulin receptor signaling pathway	4	0.01
GO.0045893	Positive regulation of transcription		
GO.0033209	Tumor necrosis factor-mediated signaling pathway	4	0.0112
GO.0050663	Cytokine secretion	4	0.0112
GO.0097028	Dendritic cell differentiation	4	0.0112
GO.0002577	Regulation of antigen processing and presentation	3	0.0116
GO.0002710	Negative regulation of t cell mediated immunity	3	0.0116
GO.0002827	Positive regulation of t-helper 1 type immune response	3	0.0116
GO.0002830	Positive regulation of type 2 immune response	3	0.0116
GO.0043306	Positive regulation of mast cell degranulation	3	0.0116
GO.0045579	Positive regulation of b cell differentiation	3	0.0116
GO.0051044	Positive regulation of membrane protein ectodomain proteolysis	3	0.0116
GO.0033674	Positive regulation of kinase activity	14	0.012
GO.0002220	Innate immune response activating cell surface receptor signaling pathway	7	0.0123
GO.0051338	Regulation of transferase activity	21	0.0124
GO.0032956	Regulation of actin cytoskeleton organization	10	0.0126
GO.0042990	Regulation of transcription factor import into nucleus	6	0.0131
GO.0043281	Regulation of cysteine-type endopeptidase activity involved in apoptotic process	9	0.0135
GO.0002544	Chronic inflammatory response	3	0.014
GO.0032609	Interferon-gamma production	3	0.014
GO.0032656	Regulation of interleukin-13 production	3	0.014
GO.0002673	Regulation of acute inflammatory response	5	0.0143
GO.0080090	Regulation of primary metabolic process	77	0.0143
GO.0019538	Protein metabolic process	61	0.0144
GO.0051173	Positive regulation of nitrogen compound metabolic process	33	0.0149
GO.0033157	Regulation of intracellular protein transport	12	0.015
GO.0031343	Positive regulation of cell killing	4	0.0151
GO.0071622	Regulation of granulocyte chemotaxis	4	0.0151
GO.0030838	Positive regulation of actin filament polymerization	5	0.0152
GO.0008285	Negative regulation of cell proliferation	17	0.0156
GO.1903707	Negative regulation of hemopoiesis	7	0.0157
GO.0031323	Regulation of cellular metabolic process	79	0.0158
GO.0032653	Regulation of interleukin-10 production	4	0.0166
GO.0042510	Regulation of tyrosine phosphorylation of stat1 protein	3	0.0166
GO.0070234	Positive regulation of t cell apoptotic process	3	0.0166

GO.0001915	Negative regulation of t cell mediated cytotoxicity	2	0.0169
GO.0033634	Positive regulation of cell-cell adhesion mediated by integrin	2	0.0169
GO.0035771	Interleukin-4-mediated signaling pathway	2	0.0169
GO.0038110	Interleukin-2-mediated signaling pathway	2	0.0169
GO.0045064	T-helper 2 cell differentiation	2	0.0169
GO.0071352	Cellular response to interleukin-2	2	0.0169
GO.1903036	Positive regulation of response to wounding	7	0.0169
GO.2000110	Negative regulation of macrophage apoptotic process	2	0.0169
GO.0060255	Regulation of macromolecule metabolic process	76	0.0172
GO.0097305	Response to alcohol	11	0.0174
GO.0001914	Regulation of t cell mediated cytotoxicity	3	0.0194
GO.0016064	Immunoglobulin mediated immune response	5	0.0194
GO.0002221	Pattern recognition receptor signaling pathway	7	0.0198
GO.0045860	Positive regulation of protein kinase activity	13	0.0198
GO.1901701	Cellular response to oxygen-containing compound	19	0.0213
GO.0016192	Vesicle-mediated transport	24	0.0228
GO.0033630	Positive regulation of cell adhesion mediated by integrin	3	0.0228
GO.0045576	Mast cell activation	3	0.0228
GO.0045921	Positive regulation of exocytosis	5	0.0235
GO.0002920	Regulation of humoral immune response	4	0.0236
GO.0032651	Regulation of interleukin-1 beta production	4	0.0236
GO.0060338	Regulation of type i interferon-mediated signaling pathway	4	0.0236
GO.1903827	Regulation of cellular protein localization	14	0.0248
GO.0010638	Positive regulation of organelle organization	16	0.0249
GO.0014066	Regulation of phosphatidylinositol 3-kinase signaling	5	0.0249
GO.0045637	Regulation of myeloid cell differentiation	8	0.0259
GO.2001242	Regulation of intrinsic apoptotic signaling pathway	7	0.0259
GO.0001820	Serotonin secretion	2	0.0263
GO.0002438	Acute inflammatory response to antigenic stimulus	2	0.0263
GO.0002767	Immune response-inhibiting cell surface receptor signaling pathway	2	0.0263
GO.0008626	Granzyme-mediated apoptotic signaling pathway	2	0.0263
GO.0032633	Interleukin-4 production	2	0.0263
GO.0034242	Negative regulation of syncytium formation by plasma membrane fusion	2	0.0263
GO.0035455	Response to interferon-alpha	3	0.0263
GO.0043011	Myeloid dendritic cell differentiation	3	0.0263

GO.0050858	Negative regulation of antigen receptor-mediated signaling pathway	3	0.0263
GO.0072538	T-helper 17 type immune response	2	0.0263
GO.0072539	T-helper 17 cell differentiation	2	0.0263
GO.2000347	Positive regulation of hepatocyte proliferation	2	0.0263
GO.0051347	Positive regulation of transferase activity	15	0.0269
GO.0045935	Positive regulation of nucleobase-containing compound metabolic process	31	0.0274
GO.0030593	Neutrophil chemotaxis	4	0.0275
GO.0090066	Regulation of anatomical structure size	11	0.0279
GO.0097190	Apoptotic signaling pathway	11	0.0285
GO.0006979	Response to oxidative stress	11	0.0292
GO.0051248	Negative regulation of protein metabolic process	22	0.0292
GO.0032461	Positive regulation of protein oligomerization	3	0.0296
GO.0090023	Positive regulation of neutrophil chemotaxis	3	0.0296
GO.1901739	Regulation of myoblast fusion	3	0.0296
GO.0045765	Regulation of angiogenesis	8	0.0316
GO.0070372	Regulation of erk1 and erk2 cascade	8	0.0337
GO.0002437	Inflammatory response to antigenic stimulus	3	0.034
GO.0032660	Regulation of interleukin-17 production	3	0.034
GO.0046903	Secretion	15	0.0343
GO.0017157	Regulation of exocytosis	7	0.0351
GO.0035666	Trif-dependent toll-like receptor signaling pathway	5	0.0361
GO.0002826	Negative regulation of t-helper 1 type immune response	2	0.0372
GO.0042092	Type 2 immune response	2	0.0372
GO.0043320	Natural killer cell degranulation	2	0.0372
GO.0045063	T-helper 1 cell differentiation	2	0.0372
GO.0045588	Positive regulation of gamma-delta t cell differentiation	2	0.0372
GO.0045862	Positive regulation of proteolysis	11	0.0372
GO.0048304	Positive regulation of isotype switching to igg isotypes	2	0.0372
GO.0060710	Chorio-allantoic fusion	2	0.0372
GO.1902715	Positive regulation of interferon-gamma secretion	2	0.0372
GO.2000503	Positive regulation of natural killer cell chemotaxis	2	0.0372
GO.0007568	Aging	9	0.0378
GO.0051125	Regulation of actin nucleation	3	0.0379
GO.1904292	Regulation of erad pathway	3	0.0379
GO.0051093	Negative regulation of developmental process	19	0.0383
GO.0030198	Extracellular matrix organization	11	0.0395
GO.0045596	Negative regulation of cell differentiation	16	0.0401
GO.0007267	Cell-cell signaling	20	0.0408

GO.0045471	Response to ethanol	6	0.041
GO.0032940	Secretion by cell	13	0.0413
GO.0048525	Negative regulation of viral process	5	0.0413
GO.0043406	Positive regulation of map kinase activity	8	0.0415
GO.0043410	Positive regulation of mapk cascade	12	0.0416
GO.0032733	Positive regulation of interleukin-10 production	3	0.042
GO.0033077	T cell differentiation in thymus	4	0.042
GO.0042993	Positive regulation of transcription factor import into nucleus	4	0.042
GO.0043124	Negative regulation of i-kappab kinase/nf-kappab signaling	4	0.042
GO.0050706	Regulation of interleukin-1 beta secretion	3	0.042
GO.0032481	Positive regulation of type i interferon production	5	0.0428
GO.0048660	Regulation of smooth muscle cell proliferation	5	0.0428
GO.0002223	Stimulatory c-type lectin receptor signaling pathway	6	0.0471
GO.0042269	Regulation of natural killer cell mediated cytotoxicity	3	0.0471
GO.0060334	Regulation of interferon-gamma-mediated signaling pathway	3	0.0471
GO.2000352	Negative regulation of endothelial cell apoptotic process	3	0.0471
GO.0001913	T cell mediated cytotoxicity	2	0.0484
GO.0002480	Antigen processing and presentation of exogenous peptide antigen via MHC class I		
GO.0043374	Cd8-positive		
GO.0045591	Positive regulation of regulatory t cell differentiation	2	0.0484
GO.0051126	Negative regulation of actin nucleation	2	0.0484
GO.0060753	Regulation of mast cell chemotaxis	2	0.0484
GO.0070424	Regulation of nucleotide-binding oligomerization domain containing signaling pathway	2	0.0484
GO.2001198	Regulation of dendritic cell differentiation	2	0.0484

Table S2.2. KEGG pathway enrichment in upregulated group of RA versus OA

**Pathway ID**	**Pathway description**	**Observed gene count**	**False discovery rate**
4650	Natural killer cell mediated cytotoxicity	21	1.27E-16
5340	Primary immunodeficiency	14	1.27E-16
4060	Cytokine-cytokine receptor interaction	27	3.14E-16
4064	NF-kappa B signaling pathway	17	1.26E-14
4062	Chemokine signaling pathway	19	1.33E-11
4660	T cell receptor signaling pathway	15	1.92E-11
5169	Epstein-Barr virus infection	18	2.92E-10
5166	HTLV-I infection	20	4.24E-10
4640	Hematopoietic cell lineage	13	4.93E-10
5162	Measles	15	6.89E-10
4514	Cell adhesion molecules (CAMs)	15	1.35E-09
4672	Intestinal immune network for IgA production	9	3.57E-08
5168	Herpes simplex infection	15	3.74E-08
4612	Antigen processing and presentation	10	7.60E-08
5330	Allograft rejection	8	7.60E-08
4662	B cell receptor signaling pathway	10	1.11E-07
5416	Viral myocarditis	9	2.03E-07
5332	Graft-versus-host disease	7	2.42E-06
4380	Osteoclast differentiation	11	2.74E-06
4620	Toll-like receptor signaling pathway	10	3.65E-06
4940	Type I diabetes mellitus	7	4.45E-06
4668	TNF signaling pathway	10	5.24E-06
5144	Malaria	7	1.10E-05
5140	Leishmaniasis	8	1.47E-05
5320	Autoimmune thyroid disease	7	1.58E-05
4630	Jak-STAT signaling pathway	11	1.91E-05
5164	Influenza A	11	3.82E-05
5323	Rheumatoid arthritis	8	6.20E-05
4670	Leukocyte transendothelial migration	9	6.26E-05
5321	Inflammatory bowel disease (IBD)	7	6.64E-05
4144	Endocytosis	11	0.000133
5143	African trypanosomiasis	5	0.000314
5142	Chagas disease (American trypanosomiasis)	7	0.00105
4145	Phagosome	8	0.00203
5203	Viral carcinogenesis	9	0.00208
5145	Toxoplasmosis	7	0.00251
5200	Pathways in cancer	12	0.00271
4666	Fc gamma R-mediated phagocytosis	6	0.00379
5202	Transcriptional misregulation in cancer	8	0.00465
4623	Cytosolic DNA-sensing pathway	5	0.00485
5152	Tuberculosis	8	0.0056
4664	Fc epsilon RI signaling pathway	5	0.00662
3050	Proteasome	4	0.00984

4722	Neurotrophin signaling pathway	6	0.014
4810	Regulation of actin cytoskeleton	8	0.0179
5222	Small cell lung cancer	5	0.0179
5160	Hepatitis C	6	0.0223
4621	NOD-like receptor signaling pathway	4	0.0234
4370	VEGF signaling pathway	4	0.0276
5120	Epithelial cell signaling in Helicobacter pylori infection	4	0.0382
4920	Adipocytokine signaling pathway	4	0.0416
4622	RIG-I-like receptor signaling pathway	4	0.0452
4010	MAPK signaling pathway	8	0.0471

Table S2.3. GO pathway enrichment in downregulated group of RA versus OA

Pathway ID	Pathway description	Observed gene count	False discovery rate
GO.0048731	System development	91	8.90E-12
GO.0007275	Multicellular organismal development	97	3.68E-11
GO.0044767	Single-organism developmental process	100	2.74E-09
GO.0048856	Anatomical structure development	91	1.29E-08
GO.0051239	Regulation of multicellular organismal process	63	5.14E-08
GO.0009653	Anatomical structure morphogenesis	58	6.65E-08
GO.0032501	Multicellular organismal process	109	6.65E-08
GO.0032502	Developmental process	96	6.65E-08
GO.0060284	Regulation of cell development	33	6.65E-08
GO.0048513	Organ development	67	7.59E-08
GO.0044707	Single-multicellular organism process	105	1.30E-07
GO.0040008	Regulation of growth	29	2.13E-07
GO.0007399	Nervous system development	54	5.27E-07
GO.0045597	Positive regulation of cell differentiation	32	6.87E-07
GO.0009888	Tissue development	46	8.63E-07
GO.0030154	Cell differentiation	70	1.46E-06
GO.0042063	Gliogenesis	15	1.46E-06
GO.0050793	Regulation of developmental process	54	2.03E-06
GO.0051128	Regulation of cellular component organization	55	2.03E-06
GO.0061061	Muscle structure development	23	2.45E-06
GO.0048519	Negative regulation of biological process	85	2.55E-06
GO.0022008	Neurogenesis	42	2.87E-06
GO.0048869	Cellular developmental process	71	3.20E-06
GO.0007517	Muscle organ development	18	3.36E-06
GO.0060537	Muscle tissue development	18	3.36E-06
GO.2000026	Regulation of multicellular organismal development	44	3.36E-06
GO.0001558	Regulation of cell growth	20	3.68E-06
GO.0030198	Extracellular matrix organization	20	4.12E-06
GO.0048589	Developmental growth	19	4.52E-06
GO.0048732	Gland development	21	5.56E-06
GO.0007155	Cell adhesion	33	6.06E-06
GO.0040007	Growth	21	6.25E-06
GO.0042127	Regulation of cell proliferation	42	6.70E-06
GO.0048523	Negative regulation of cellular process	79	6.70E-06
GO.0014706	Striated muscle tissue development	17	8.25E-06
GO.0090092	Regulation of transmembrane receptor protein serine/threonine kinase signaling pathway	15	1.01E-05
GO.0072358	Cardiovascular system development	29	1.08E-05
GO.0072359	Circulatory system development	29	1.08E-05

GO.0051241	Negative regulation of multicellular organismal process	32	1.81E-05
GO.0001655	Urogenital system development	17	2.13E-05
GO.0032330	Regulation of chondrocyte differentiation	8	2.13E-05
GO.0050767	Regulation of neurogenesis	24	2.13E-05
GO.0051960	Regulation of nervous system development	26	2.13E-05
GO.0010720	Positive regulation of cell development	21	2.39E-05
GO.0065008	Regulation of biological quality	64	2.75E-05
GO.0051093	Negative regulation of developmental process	29	3.87E-05
GO.0007417	Central nervous system development	29	4.10E-05
GO.0045664	Regulation of neuron differentiation	21	4.38E-05
GO.0048583	Regulation of response to stimulus	68	4.46E-05
GO.0023051	Regulation of signaling	59	4.89E-05
GO.0007167	Enzyme linked receptor protein signaling pathway	30	5.69E-05
GO.0001822	Kidney development	15	5.89E-05
GO.0042221	Response to chemical	73	6.49E-05
GO.0001503	Ossification	15	7.44E-05
GO.0051094	Positive regulation of developmental process	33	7.65E-05
GO.0071363	Cellular response to growth factor stimulus	24	7.97E-05
GO.0070887	Cellular response to chemical stimulus	52	8.88E-05
GO.0045595	Regulation of cell differentiation	39	9.36E-05
GO.0072001	Renal system development	15	9.85E-05
GO.0010001	Glial cell differentiation	11	0.000104
GO.0022603	Regulation of anatomical structure morphogenesis	28	0.000123
GO.0048468	Cell development	41	0.000123
GO.0010033	Response to organic substance	54	0.000137
GO.0061037	Negative regulation of cartilage development	6	0.000154
GO.0090287	Regulation of cellular response to growth factor stimulus	14	0.000154
GO.0051270	Regulation of cellular component movement	25	0.000155
GO.0061448	Connective tissue development	13	0.000182
GO.0010646	Regulation of cell communication	59	0.000203
GO.0061035	Regulation of cartilage development	8	0.000209
GO.0001501	Skeletal system development	19	0.000258
GO.2000145	Regulation of cell motility	23	0.000258
GO.0016477	Cell migration	25	0.000306
GO.0051962	Positive regulation of nervous system development	18	0.000315
GO.0072191	Ureter smooth muscle development	3	0.000319
GO.0072193	Ureter smooth muscle cell differentiation	3	0.000319
GO.0030334	Regulation of cell migration	22	0.000321
GO.0001654	Eye development	16	0.000323

GO.2000241	Regulation of reproductive process	9	0.000323
GO.0042692	Muscle cell differentiation	14	0.000333
GO.0048699	Generation of neurons	35	0.000364
GO.0043010	Camera-type eye development	15	0.000391
GO.0001101	Response to acid chemical	15	0.000399
GO.0048522	Positive regulation of cellular process	78	0.000399
GO.0032331	Negative regulation of chondrocyte differentiation	5	0.00042
GO.0051240	Positive regulation of multicellular organismal process	35	0.000443
GO.0001944	Vasculature development	19	0.000459
GO.0009966	Regulation of signal transduction	51	0.000523
GO.0030510	Regulation of BMP signaling pathway	8	0.000561
GO.0050678	Regulation of epithelial cell proliferation	14	0.000623
GO.0050769	Positive regulation of neurogenesis	16	0.000667
GO.0048518	Positive regulation of biological process	85	0.000858
GO.0008285	Negative regulation of cell proliferation	22	0.00089
GO.0060429	Epithelium development	28	0.000891
GO.0045666	Positive regulation of neuron differentiation	14	0.000951
GO.0044597	Daunorubicin metabolic process	3	0.000986
GO.0044598	Doxorubicin metabolic process	3	0.000986
GO.0071310	Cellular response to organic substance	42	0.000986
GO.0000904	Cell morphogenesis involved in differentiation	22	0.001
GO.0051674	Localization of cell	25	0.00102
GO.0007050	Cell cycle arrest	10	0.00103
GO.0007166	Cell surface receptor signaling pathway	45	0.00103
GO.0048598	Embryonic morphogenesis	20	0.00109
GO.2000736	Regulation of stem cell differentiation	9	0.00109
GO.0032268	Regulation of cellular protein metabolic process	48	0.00111
GO.0001932	Regulation of protein phosphorylation	31	0.00121
GO.0051146	Striated muscle cell differentiation	11	0.00121
GO.0051271	Negative regulation of cellular component movement	12	0.00127
GO.0006629	Lipid metabolic process	29	0.00128
GO.0048705	Skeletal system morphogenesis	12	0.00131
GO.0033993	Response to lipid	24	0.00134
GO.0040011	Locomotion	31	0.00134
GO.0042592	Homeostatic process	33	0.00138
GO.0001657	Ureteric bud development	8	0.00145
GO.0006928	Movement of cell or subcellular component	33	0.00154
GO.0050794	Regulation of cellular process	130	0.0016
GO.0032989	Cellular component morphogenesis	28	0.00162
GO.0035272	Exocrine system development	6	0.00168
GO.0007154	Cell communication	84	0.00179
GO.0040012	Regulation of locomotion	22	0.00179

GO.0051216	Cartilage development	10	0.00179
GO.0001823	Mesonephros development	8	0.00186
GO.0009719	Response to endogenous stimulus	34	0.00188
GO.0030336	Negative regulation of cell migration	11	0.00188
GO.0035270	Endocrine system development	9	0.00188
GO.0065009	Regulation of molecular function	53	0.00188
GO.0007507	Heart development	17	0.00193
GO.0023057	Negative regulation of signaling	30	0.00207
GO.0009887	Organ morphogenesis	25	0.00214
GO.0032879	Regulation of localization	47	0.00214
GO.0007423	Sensory organ development	18	0.00221
GO.0009968	Negative regulation of signal transduction	28	0.00226
GO.0051246	Regulation of protein metabolic process	49	0.00226
GO.0010648	Negative regulation of cell communication	30	0.00231
GO.0045596	Negative regulation of cell differentiation	21	0.00231
GO.0061036	Positive regulation of cartilage development	5	0.00236
GO.0000902	Cell morphogenesis	26	0.00238
GO.2000738	Positive regulation of stem cell differentiation	6	0.00238
GO.1901343	Negative regulation of vasculature development	7	0.00263
GO.0048585	Negative regulation of response to stimulus	32	0.00267
GO.0051726	Regulation of cell cycle	26	0.00271
GO.0016202	Regulation of striated muscle tissue development	8	0.00287
GO.0051336	Regulation of hydrolase activity	31	0.00296
GO.0048553	Negative regulation of metalloenzyme activity	3	0.0031
GO.0051045	Negative regulation of membrane protein ectodomain proteolysis	3	0.0031
GO.1901700	Response to oxygen-containing compound	32	0.0031
GO.0050789	Regulation of biological process	132	0.00312
GO.0042325	Regulation of phosphorylation	31	0.00324
GO.0022604	Regulation of cell morphogenesis	17	0.00335
GO.0051716	Cellular response to stimulus	93	0.00335
GO.0050896	Response to stimulus	104	0.00368
GO.0022612	Gland morphogenesis	8	0.00378
GO.1901342	Regulation of vasculature development	11	0.00378
GO.0060065	Uterus development	4	0.00385
GO.0060560	Developmental growth involved in morphogenesis	8	0.004
GO.0009605	Response to external stimulus	40	0.0041
GO.0010721	Negative regulation of cell development	12	0.0041
GO.0044255	Cellular lipid metabolic process	23	0.00416
GO.0001656	Metanephros development	7	0.00419
GO.0032332	Positive regulation of chondrocyte differentiation	4	0.00472

GO.0032808	Lacrimal gland development	3	0.00491
GO.0052547	Regulation of peptidase activity	15	0.00492
GO.1902533	Positive regulation of intracellular signal transduction	23	0.00504
GO.0048738	Cardiac muscle tissue development	9	0.00533
GO.0051098	Regulation of binding	12	0.00533
GO.0016049	Cell growth	8	0.00552
GO.0050680	Negative regulation of epithelial cell proliferation	8	0.00552
GO.0023056	Positive regulation of signaling	33	0.00565
GO.0045786	Negative regulation of cell cycle	16	0.00569
GO.0044700	Single organism signaling	80	0.00577
GO.0055123	Digestive system development	9	0.00577
GO.0010632	Regulation of epithelial cell migration	9	0.00599
GO.0001568	Blood vessel development	16	0.00601
GO.0042327	Positive regulation of phosphorylation	23	0.00619
GO.0009790	Embryo development	25	0.00649
GO.0034308	Primary alcohol metabolic process	5	0.00697
GO.0045937	Positive regulation of phosphate metabolic process	25	0.00698
GO.0048709	Oligodendrocyte differentiation	6	0.00721
GO.0044092	Negative regulation of molecular function	27	0.00735
GO.0051248	Negative regulation of protein metabolic process	26	0.00767
GO.2000242	Negative regulation of reproductive process	5	0.00767
GO.0048771	Tissue remodeling	7	0.00782
GO.0007519	Skeletal muscle tissue development	8	0.00809
GO.0045765	Regulation of angiogenesis	10	0.00809
GO.0035282	Segmentation	7	0.00822
GO.0008284	Positive regulation of cell proliferation	22	0.00851
GO.0001934	Positive regulation of protein phosphorylation	22	0.00863
GO.0010634	Positive regulation of epithelial cell migration	7	0.00868
GO.0010717	Regulation of epithelial to mesenchymal transition	6	0.00888
GO.0001502	Cartilage condensation	4	0.00895
GO.0032270	Positive regulation of cellular protein metabolic process	30	0.00895
GO.0035295	Tube development	18	0.00895
GO.0043567	Regulation of insulin-like growth factor receptor signaling pathway	4	0.00895
GO.0052548	Regulation of endopeptidase activity	14	0.00895
GO.0065007	Biological regulation	133	0.00895
GO.0090100	Positive regulation of transmembrane receptor protein serine/threonine kinase signaling pathway	7	0.00895

GO.0030850	Prostate gland development	5	0.00896
GO.0010975	Regulation of neuron projection development	13	0.00929
GO.0033689	Negative regulation of osteoblast proliferation	3	0.00929
GO.0009967	Positive regulation of signal transduction	30	0.0094
GO.0048584	Positive regulation of response to stimulus	39	0.00942
GO.0006714	Sesquiterpenoid metabolic process	2	0.00949
GO.0043627	Response to estrogen	10	0.00949
GO.0007169	Transmembrane receptor protein tyrosine kinase signaling pathway	20	0.00956
GO.0022414	Reproductive process	28	0.00967
GO.0016525	Negative regulation of angiogenesis	6	0.00968
GO.0019220	Regulation of phosphate metabolic process	33	0.00999
GO.0072073	Kidney epithelium development	8	0.00999
GO.2000826	Regulation of heart morphogenesis	4	0.00999
GO.0044711	Single-organism biosynthetic process	30	0.0107
GO.0007173	Epidermal growth factor receptor signaling pathway	10	0.0108
GO.0048706	Embryonic skeletal system development	8	0.011
GO.0031399	Regulation of protein modification process	34	0.0113
GO.0071709	Membrane assembly	4	0.0115
GO.0035265	Organ growth	6	0.0118
GO.0051247	Positive regulation of protein metabolic process	31	0.0118
GO.0007548	Sex differentiation	11	0.0119
GO.0008354	Germ cell migration	3	0.0119
GO.0048568	Embryonic organ development	15	0.0119
GO.0060538	Skeletal muscle organ development	8	0.0123
GO.0071495	Cellular response to endogenous stimulus	25	0.0129
GO.0060740	Prostate gland epithelium morphogenesis	4	0.013
GO.0030278	Regulation of ossification	9	0.0138
GO.0032269	Negative regulation of cellular protein metabolic process	24	0.0139
GO.2000147	Positive regulation of cell motility	13	0.014
GO.0003007	Heart morphogenesis	10	0.0148
GO.0001658	Branching involved in ureteric bud morphogenesis	5	0.0149
GO.0044272	Sulfur compound biosynthetic process	9	0.0149
GO.0032970	Regulation of actin filament-based process	12	0.0154
GO.0008283	Cell proliferation	19	0.0157
GO.0048545	Response to steroid hormone	14	0.0157
GO.0045667	Regulation of osteoblast differentiation	7	0.016
GO.0016101	Diterpenoid metabolic process	6	0.0172
GO.0048871	Multicellular organismal homeostasis	11	0.0174
GO.0031401	Positive regulation of protein modification process	25	0.0175

GO.0035239	Tube morphogenesis	13	0.0178
GO.0046824	Positive regulation of nucleocytoplasmic transport	7	0.0186
GO.0010604	Positive regulation of macromolecule metabolic process	48	0.0189
GO.0031128	Developmental induction	4	0.0189
GO.0060512	Prostate gland morphogenesis	4	0.0189
GO.0010647	Positive regulation of cell communication	33	0.019
GO.0072044	Collecting duct development	3	0.0193
GO.0090101	Negative regulation of transmembrane receptor protein serine/threonine kinase signaling pathway	7	0.0193
GO.0048729	Tissue morphogenesis	17	0.0202
GO.0055002	Striated muscle cell development	7	0.0202
GO.0072006	Nephron development	7	0.0202
GO.0043410	Positive regulation of MAPK cascade	14	0.0204
GO.0044091	Membrane biogenesis	4	0.0209
GO.0044344	Cellular response to fibroblast growth factor stimulus	9	0.0212
GO.0048638	Regulation of developmental growth	11	0.0212
GO.0050679	Positive regulation of epithelial cell proliferation	8	0.0213
GO.0051179	Localization	69	0.0213
GO.0010035	Response to inorganic substance	14	0.0215
GO.0019448	L-cysteine catabolic process	2	0.0215
GO.0030162	Regulation of proteolysis	20	0.0215
GO.0044057	Regulation of system process	14	0.0215
GO.0045726	Positive regulation of integrin biosynthetic process	2	0.0215
GO.0046903	Secretion	17	0.0215
GO.0048660	Regulation of smooth muscle cell proliferation	6	0.0215
GO.0060322	Head development	19	0.0215
GO.0060395	SMAD protein signal transduction	5	0.0215
GO.0072190	Ureter urothelium development	2	0.0215
GO.0072197	Ureter morphogenesis	2	0.0215
GO.0043523	Regulation of neuron apoptotic process	9	0.0217
GO.0014068	Positive regulation of phosphatidylinositol 3-kinase signaling	5	0.022
GO.0044763	Single-organism cellular process	134	0.022
GO.0048546	Digestive tract morphogenesis	5	0.022
GO.0060038	Cardiac muscle cell proliferation	3	0.022
GO.0060442	Branching involved in prostate gland morphogenesis	3	0.022
GO.0060675	Ureteric bud morphogenesis	5	0.022
GO.0060766	Negative regulation of androgen receptor signaling pathway	3	0.022

GO.0051896	Regulation of protein kinase B signaling	7	0.0235
GO.0010951	Negative regulation of endopeptidase activity	10	0.0239
GO.1901214	Regulation of neuron death	10	0.0239
GO.0006790	Sulfur compound metabolic process	12	0.0241
GO.0043393	Regulation of protein binding	8	0.0241
GO.0048514	Blood vessel morphogenesis	13	0.0242
GO.1901615	Organic hydroxy compound metabolic process	15	0.0253
GO.0010718	Positive regulation of epithelial to mesenchymal transition	4	0.0271
GO.0051493	Regulation of cytoskeleton organization	13	0.0272
GO.0043408	Regulation of MAPK cascade	17	0.0273
GO.0038179	Neurotrophin signaling pathway	11	0.0284
GO.0048562	Embryonic organ morphogenesis	11	0.0291
GO.0060548	Negative regulation of cell death	22	0.0293
GO.0030335	Positive regulation of cell migration	12	0.0298
GO.0031344	Regulation of cell projection organization	14	0.0301
GO.0044089	Positive regulation of cellular component biogenesis	12	0.0304
GO.0060350	Endochondral bone morphogenesis	5	0.0309
GO.0030182	Neuron differentiation	23	0.0314
GO.0007165	Signal transduction	72	0.0316
GO.0030277	Maintenance of gastrointestinal epithelium	3	0.0316
GO.0030855	Epithelial cell differentiation	15	0.0317
GO.0060348	Bone development	8	0.0317
GO.0030879	Mammary gland development	7	0.0323
GO.0032355	Response to estradiol	7	0.0323
GO.0044281	Small molecule metabolic process	40	0.0325
GO.0030203	Glycosaminoglycan metabolic process	8	0.0326
GO.0030111	Regulation of Wnt signaling pathway	11	0.0328
GO.0009991	Response to extracellular stimulus	14	0.0329
GO.0007568	Aging	10	0.0332
GO.0045861	Negative regulation of proteolysis	12	0.0332
GO.0006066	Alcohol metabolic process	12	0.0338
GO.0001974	Blood vessel remodeling	4	0.0347
GO.0008543	Fibroblast growth factor receptor signaling pathway	8	0.0347
GO.0032956	Regulation of actin cytoskeleton organization	10	0.0347
GO.0042307	Positive regulation of protein import into nucleus	6	0.0347
GO.0044848	Biological phase	2	0.0347
GO.0048646	Anatomical structure formation involved in morphogenesis	23	0.0347
GO.0060283	Negative regulation of oocyte development	2	0.0347
GO.0060428	Lung epithelium development	4	0.0347

GO.0060686	Negative regulation of prostatic bud formation	2	0.0347
GO.0061551	Trigeminal ganglion development	2	0.0347
GO.0072095	Regulation of branch elongation involved in ureteric bud branching	2	0.0347
GO.0072199	Regulation of mesenchymal cell proliferation involved in ureter development	2	0.0347
GO.0090191	Negative regulation of branching involved in ureteric bud morphogenesis	2	0.0347
GO.0097118	Neuroligin clustering involved in postsynaptic membrane assembly	2	0.0347
GO.1902531	Regulation of intracellular signal transduction	29	0.0347
GO.0009725	Response to hormone	21	0.0352
GO.2000136	Regulation of cell proliferation involved in heart morphogenesis	3	0.0353
GO.0051145	Smooth muscle cell differentiation	4	0.0367
GO.0051496	Positive regulation of stress fiber assembly	4	0.0367
GO.0001894	Tissue homeostasis	8	0.0396
GO.0032844	Regulation of homeostatic process	12	0.0397
GO.0002088	Lens development in camera-type eye	5	0.0398
GO.0007010	Cytoskeleton organization	20	0.04
GO.0045668	Negative regulation of osteoblast differentiation	4	0.04
GO.0048704	Embryonic skeletal system morphogenesis	6	0.04
GO.0090288	Negative regulation of cellular response to growth factor stimulus	7	0.0403
GO.0001759	Organ induction	3	0.0406
GO.0010941	Regulation of cell death	30	0.0406
GO.0014812	Muscle cell migration	3	0.0406
GO.0060541	Respiratory system development	9	0.0406
GO.1901135	Carbohydrate derivative metabolic process	23	0.0407
GO.0048608	Reproductive structure development	13	0.0413
GO.0010464	Regulation of mesenchymal cell proliferation	4	0.0432
GO.0030500	Regulation of bone mineralization	5	0.0443
GO.0061458	Reproductive system development	13	0.0449
GO.0044087	Regulation of cellular component biogenesis	17	0.045
GO.0045926	Negative regulation of growth	9	0.0455
GO.0061138	Morphogenesis of a branching epithelium	8	0.0455
GO.0051130	Positive regulation of cellular component organization	25	0.0461
GO.0007389	Pattern specification process	13	0.0469
GO.0021987	Cerebral cortex development	6	0.0469
GO.0030903	Notochord development	3	0.0469
GO.0045598	Regulation of fat cell differentiation	6	0.0469

GO.0050790	Regulation of catalytic activity	40	0.0469
GO.0048565	Digestive tract development	7	0.0475
GO.0097305	Response to alcohol	11	0.0475
GO.0042698	Ovulation cycle	6	0.049
GO.0048015	Phosphatidylinositol-mediated signaling	7	0.0492
GO.0048592	Eye morphogenesis	7	0.0492

Table S2.4. KEGG pathway enrichment in downregulated group of RA versus OA

Pathway ID	Pathway description	Observed gene count	False discovery rate
561	Glycerolipid metabolism	6	0.0142
5202	Transcriptional misregulation in cancer	9	0.0276

Table S3. GO and KEGG enrichment pathways in RA versus normalcy and OA versus normalcy.

Table S3.1. GO pathway enrichment in upregulated group of RA versus normalcy

Pathway ID	Pathway description	Observed gene count	False discovery rate
GO.0006955	Immune response	112	1.57E-44
GO.0002376	Immune system process	130	8.05E-41
GO.0006952	Defense response	92	1.18E-26
GO.0007166	Cell surface receptor signaling pathway	109	5.62E-25
GO.0006950	Response to stress	141	2.38E-23
GO.0050776	Regulation of immune response	63	4.92E-21
GO.0002252	Immune effector process	47	9.51E-21
GO.0050896	Response to stimulus	205	2.57E-20
GO.0002682	Regulation of immune system process	80	4.00E-20
GO.0050778	Positive regulation of immune response	51	1.32E-19
GO.0002684	Positive regulation of immune system process	61	2.22E-19
GO.0045087	Innate immune response	65	2.52E-19
GO.0007165	Signal transduction	157	1.62E-18
GO.0023052	Signaling	162	6.57E-18
GO.0051716	Cellular response to stimulus	181	7.71E-18
GO.0019221	Cytokine-mediated signaling pathway	41	8.79E-18
GO.0044700	Single organism signaling	161	1.41E-17
GO.0034097	Response to cytokine	51	2.08E-17
GO.0007154	Cell communication	162	4.74E-17
GO.0071345	Cellular response to cytokine stimulus	46	8.56E-17
GO.0009607	Response to biotic stimulus	51	1.75E-15
GO.0002757	Immune response-activating signal transduction	38	1.85E-15
GO.0048584	Positive regulation of response to stimulus	86	6.60E-15
GO.0051707	Response to other organism	49	6.60E-15
GO.0031347	Regulation of defense response	51	8.78E-15
GO.0002253	Activation of immune response	39	1.20E-14
GO.0010033	Response to organic substance	99	2.08E-14
GO.0048583	Regulation of response to stimulus	119	2.16E-14
GO.0031349	Positive regulation of defense response	36	3.64E-14
GO.0009605	Response to external stimulus	83	7.21E-14
GO.0051704	Multi-organism process	90	4.22E-13
GO.0035556	Intracellular signal transduction	79	1.79E-12
GO.0002764	Immune response-regulating signaling pathway	38	2.06E-12
GO.0009615	Response to virus	27	2.96E-12
GO.0070887	Cellular response to chemical stimulus	89	7.57E-12
GO.0050865	Regulation of cell activation	35	8.82E-12

GO.0002429	Immune response-activating cell surface receptor signaling pathway	28	1.94E-11
GO.0045088	Regulation of innate immune response	32	1.94E-11
GO.0048518	Positive regulation of biological process	146	2.27E-11
GO.0001817	Regulation of cytokine production	38	2.29E-11
GO.0071310	Cellular response to organic substance	77	2.32E-11
GO.0002694	Regulation of leukocyte activation	33	2.51E-11
GO.0050867	Positive regulation of cell activation	27	2.77E-11
GO.0046649	Lymphocyte activation	30	3.38E-11
GO.0080134	Regulation of response to stress	65	4.18E-11
GO.0048519	Negative regulation of biological process	129	8.49E-11
GO.0048522	Positive regulation of cellular process	131	9.81E-11
GO.0042221	Response to chemical	118	1.32E-10
GO.0002696	Positive regulation of leukocyte activation	25	5.01E-10
GO.0030595	Leukocyte chemotaxis	17	5.32E-10
GO.0006915	Apoptotic process	53	7.74E-10
GO.0006954	Inflammatory response	32	1.06E-09
GO.0012501	Programmed cell death	53	1.06E-09
GO.0008219	Cell death	54	1.21E-09
GO.0098542	Defense response to other organism	29	1.37E-09
GO.0048523	Negative regulation of cellular process	119	1.43E-09
GO.0050900	Leukocyte migration	24	1.64E-09
GO.0045089	Positive regulation of innate immune response	26	1.92E-09
GO.0065009	Regulation of molecular function	92	1.99E-09
GO.0051249	Regulation of lymphocyte activation	28	2.09E-09
GO.0060326	Cell chemotaxis	20	2.73E-09
GO.0001819	Positive regulation of cytokine production	28	7.38E-09
GO.0002768	Immune response-regulating cell surface receptor signaling pathway	29	7.38E-09
GO.0044093	Positive regulation of molecular function	67	7.62E-09
GO.0051607	Defense response to virus	19	1.05E-08
GO.0045321	Leukocyte activation	29	1.28E-08
GO.0051251	Positive regulation of lymphocyte activation	22	1.29E-08
GO.0009966	Regulation of signal transduction	83	1.55E-08
GO.0002697	Regulation of immune effector process	29	1.69E-08
GO.0009967	Positive regulation of signal transduction	56	2.30E-08
GO.0050790	Regulation of catalytic activity	78	2.66E-08
GO.0072676	Lymphocyte migration	10	3.45E-08
GO.0050794	Regulation of cellular process	206	6.03E-08
GO.0065008	Regulation of biological quality	94	9.59E-08
GO.0065007	Biological regulation	216	1.11E-07
GO.0023056	Positive regulation of signaling	58	1.15E-07
GO.0050878	Regulation of body fluid levels	37	1.18E-07
GO.0009893	Positive regulation of metabolic process	104	1.23E-07

GO.0023051	Regulation of signaling	87	1.60E-07
GO.0007159	Leukocyte cell-cell adhesion	22	2.43E-07
GO.0030155	Regulation of cell adhesion	33	2.85E-07
GO.0050789	Regulation of biological process	209	2.86E-07
GO.0050851	Antigen receptor-mediated signaling pathway	15	2.94E-07
GO.0009617	Response to bacterium	29	3.61E-07
GO.0051336	Regulation of hydrolase activity	52	3.81E-07
GO.0060337	Type i interferon signaling pathway	12	4.14E-07
GO.0071357	Cellular response to type i interferon	12	4.14E-07
GO.0002250	Adaptive immune response	17	4.68E-07
GO.0002218	Activation of innate immune response	21	4.87E-07
GO.0045785	Positive regulation of cell adhesion	24	9.09E-07
GO.1902105	Regulation of leukocyte differentiation	20	9.91E-07
GO.0007599	Hemostasis	31	1.01E-06
GO.1903047	Mitotic cell cycle process	37	1.01E-06
GO.0031325	Positive regulation of cellular metabolic process	87	1.04E-06
GO.0043085	Positive regulation of catalytic activity	55	1.08E-06
GO.0010604	Positive regulation of macromolecule metabolic process	83	1.14E-06
GO.0007167	Enzyme linked receptor protein signaling pathway	42	1.17E-06
GO.0034341	Response to interferon-gamma	14	1.17E-06
GO.0002758	Innate immune response-activating signal transduction	20	1.53E-06
GO.0010646	Regulation of cell communication	87	1.53E-06
GO.0000278	Mitotic cell cycle	39	1.67E-06
GO.0030162	Regulation of proteolysis	37	1.67E-06
GO.0051345	Positive regulation of hydrolase activity	39	1.67E-06
GO.0001775	Cell activation	33	1.90E-06
GO.0010647	Positive regulation of cell communication	58	1.95E-06
GO.0007169	Transmembrane receptor protein tyrosine kinase signaling pathway	35	2.08E-06
GO.0048534	Hematopoietic or lymphoid organ development	31	2.16E-06
GO.0002460	Adaptive immune response based on somatic recombination of immune receptors built from immunoglobulin superfamily domains	14	2.18E-06
GO.0071593	Lymphocyte aggregation	19	2.18E-06
GO.0048247	Lymphocyte chemotaxis	7	2.27E-06
GO.0030098	Lymphocyte differentiation	18	2.34E-06
GO.0060333	Interferon-gamma-mediated signaling pathway	11	2.34E-06
GO.0032496	Response to lipopolysaccharide	21	2.44E-06

GO.2000116	Regulation of cysteine-type endopeptidase activity	19	2.44E-06
GO.0022409	Positive regulation of cell-cell adhesion	18	2.47E-06
GO.0007596	Blood coagulation	30	2.50E-06
GO.1903557	Positive regulation of tumor necrosis factor superfamily cytokine production	10	2.50E-06
GO.2000406	Positive regulation of t cell migration	7	3.25E-06
GO.0033993	Response to lipid	37	3.38E-06
GO.0002703	Regulation of leukocyte mediated immunity	15	3.44E-06
GO.0016477	Cell migration	36	3.46E-06
GO.0002521	Leukocyte differentiation	21	4.30E-06
GO.0097296	Activation of cysteine-type endopeptidase activity involved in apoptotic signaling pathway	7	4.69E-06
GO.0002520	Immune system development	31	4.82E-06
GO.0070098	Chemokine-mediated signaling pathway	9	5.00E-06
GO.0051246	Regulation of protein metabolic process	76	5.21E-06
GO.0097202	Activation of cysteine-type endopeptidase activity	13	5.24E-06
GO.0002699	Positive regulation of immune effector process	15	5.25E-06
GO.1903039	Positive regulation of leukocyte cell-cell adhesion	16	5.93E-06
GO.0040011	Locomotion	47	6.10E-06
GO.0010950	Positive regulation of endopeptidase activity	15	6.21E-06
GO.0051247	Positive regulation of protein metabolic process	52	6.32E-06
GO.0045058	T cell selection	8	6.76E-06
GO.0050853	B cell receptor signaling pathway	8	6.76E-06
GO.0071346	Cellular response to interferon-gamma	12	7.56E-06
GO.0006928	Movement of cell or subcellular component	50	7.98E-06
GO.0042110	T cell activation	18	7.99E-06
GO.0042035	Regulation of cytokine biosynthetic process	12	8.36E-06
GO.0002456	T cell mediated immunity	7	8.77E-06
GO.0034109	Homotypic cell-cell adhesion	20	9.38E-06
GO.0007049	Cell cycle	50	9.80E-06
GO.0030097	Hemopoiesis	28	9.98E-06
GO.0042060	Wound healing	33	1.12E-05
GO.0044403	Symbiosis		
GO.0022407	Regulation of cell-cell adhesion	22	1.17E-05
GO.1902531	Regulation of intracellular signal transduction	51	1.18E-05

GO.1903037	Regulation of leukocyte cell-cell adhesion	19	1.20E-05
GO.0032101	Regulation of response to external stimulus	38	1.24E-05
GO.0043067	Regulation of programmed cell death	51	1.25E-05
GO.2000401	Regulation of lymphocyte migration	8	1.31E-05
GO.0038093	Fc receptor signaling pathway	18	1.35E-05
GO.2001056	Positive regulation of cysteine-type endopeptidase activity	14	1.39E-05
GO.0002705	Positive regulation of leukocyte mediated immunity	11	1.42E-05
GO.0006919	Activation of cysteine-type endopeptidase activity involved in apoptotic process	12	1.53E-05
GO.0006935	Chemotaxis	31	1.53E-05
GO.0016337	Single organismal cell-cell adhesion	25	1.54E-05
GO.0032760	Positive regulation of tumor necrosis factor production	9	1.64E-05
GO.1901700	Response to oxygen-containing compound	49	1.79E-05
GO.0052548	Regulation of endopeptidase activity	23	1.95E-05
GO.0043281	Regulation of cysteine-type endopeptidase activity involved in apoptotic process	17	1.99E-05
GO.0050870	Positive regulation of t cell activation	15	2.01E-05
GO.0008283	Cell proliferation	32	2.05E-05
GO.0072678	T cell migration	6	2.05E-05
GO.0051674	Localization of cell	36	2.07E-05
GO.0042981	Regulation of apoptotic process	50	2.22E-05
GO.0032649	Regulation of interferon-gamma production	11	2.45E-05
GO.0044763	Single-organism cellular process	211	2.45E-05
GO.0030217	T cell differentiation	13	2.63E-05
GO.0070201	Regulation of establishment of protein localization	35	2.95E-05
GO.0050863	Regulation of t cell activation	18	3.11E-05
GO.0032880	Regulation of protein localization	38	3.46E-05
GO.0032729	Positive regulation of interferon-gamma production	9	3.60E-05
GO.0050729	Positive regulation of inflammatory response	11	3.81E-05
GO.0022402	Cell cycle process	41	3.96E-05
GO.1902107	Positive regulation of leukocyte differentiation	13	4.03E-05
GO.0002449	Lymphocyte mediated immunity	12	4.37E-05
GO.0043900	Regulation of multi-organism process	25	4.77E-05
GO.0045619	Regulation of lymphocyte differentiation	13	4.77E-05
GO.0060255	Regulation of macromolecule metabolic process	129	4.98E-05

GO.0032268	Regulation of cellular protein metabolic process	69	5.18E-05
GO.0097190	Apoptotic signaling pathway	21	5.36E-05
GO.0042127	Regulation of cell proliferation	51	5.44E-05
GO.0032879	Regulation of localization	69	5.92E-05
GO.0010819	Regulation of t cell chemotaxis	5	5.98E-05
GO.0042102	Positive regulation of t cell proliferation	10	6.12E-05
GO.0006968	Cellular defense response	9	6.26E-05
GO.1903555	Regulation of tumor necrosis factor superfamily cytokine production	11	6.26E-05
GO.0038096	Fc-gamma receptor signaling pathway involved in phagocytosis	10	6.73E-05
GO.0060341	Regulation of cellular localization	44	6.73E-05
GO.0009611	Response to wounding	33	7.02E-05
GO.0051050	Positive regulation of transport	36	7.08E-05
GO.0097529	Myeloid leukocyte migration	10	8.45E-05
GO.0045862	Positive regulation of proteolysis	21	9.25E-05
GO.0045621	Positive regulation of lymphocyte differentiation	10	9.47E-05
GO.0033209	Tumor necrosis factor-mediated signaling pathway	7	9.86E-05
GO.0006909	Phagocytosis	14	9.89E-05
GO.0032844	Regulation of homeostatic process	21	9.98E-05
GO.0006508	Proteolysis	41	0.000107
GO.0022610	Biological adhesion	38	0.000107
GO.0032270	Positive regulation of cellular protein metabolic process	46	0.000107
GO.0002224	Toll-like receptor signaling pathway	12	0.000109
GO.0043901	Negative regulation of multi-organism process	13	0.000116
GO.0007264	Small gtpase mediated signal transduction	30	0.000117
GO.0007093	Mitotic cell cycle checkpoint	14	0.000119
GO.0050864	Regulation of b cell activation	11	0.000131
GO.0002443	Leukocyte mediated immunity	13	0.000133
GO.0019835	Cytolysis	6	0.000133
GO.0051239	Regulation of multicellular organismal process	69	0.000152
GO.0050670	Regulation of lymphocyte proliferation	14	0.000156
GO.1903034	Regulation of response to wounding	21	0.000156
GO.0016032	Viral process	31	0.000157
GO.0050727	Regulation of inflammatory response	17	0.000159
GO.0032673	Regulation of interleukin-4 production	6	0.00017
GO.0042113	B cell activation	12	0.000173
GO.0019222	Regulation of metabolic process	144	0.000187
GO.0071222	Cellular response to lipopolysaccharide	12	0.000187
GO.0032663	Regulation of interleukin-2 production	8	0.000194

GO.0080090	Regulation of primary metabolic process	127	0.000195
GO.0050671	Positive regulation of lymphocyte proliferation	11	0.000198
GO.0071216	Cellular response to biotic stimulus	13	0.000198
GO.0045579	Positive regulation of b cell differentiation	5	0.000203
GO.0050852	T cell receptor signaling pathway	10	0.000208
GO.0007155	Cell adhesion	37	0.000218
GO.0043122	Regulation of i-kappab kinase/nf-kappab signaling	16	0.000222
GO.0051223	Regulation of protein transport	31	0.000223
GO.0043123	Positive regulation of i-kappab kinase/nf-kappab signaling	14	0.000232
GO.0050854	Regulation of antigen receptor-mediated signaling pathway	7	0.000239
GO.0051240	Positive regulation of multicellular organismal process	46	0.00024
GO.0043065	Positive regulation of apoptotic process	26	0.00025
GO.0031323	Regulation of cellular metabolic process	130	0.000258
GO.0010942	Positive regulation of cell death	27	0.00027
GO.0032680	Regulation of tumor necrosis factor production	10	0.000272
GO.0051604	Protein maturation	17	0.000289
GO.0098609	Cell-cell adhesion	26	0.000351
GO.0016485	Protein processing	16	0.000385
GO.0002407	Dendritic cell chemotaxis	5	0.000394
GO.0046777	Protein autophosphorylation	14	0.000503
GO.0002706	Regulation of lymphocyte mediated immunity	10	0.000521
GO.0045061	Thymic t cell selection	5	0.000542
GO.0070233	Negative regulation of t cell apoptotic process	5	0.000542
GO.0002366	Leukocyte activation involved in immune response	11	0.000559
GO.0034142	Toll-like receptor 4 signaling pathway	10	0.000564
GO.0042592	Homeostatic process	44	0.000606
GO.0002687	Positive regulation of leukocyte migration	10	0.000612
GO.0051281	Positive regulation of release of sequestered calcium ion into cytosol	6	0.000622
GO.0010818	T cell chemotaxis	4	0.000657
GO.0050715	Positive regulation of cytokine secretion	9	0.00066
GO.1902533	Positive regulation of intracellular signal transduction	32	0.00068
GO.0032753	Positive regulation of interleukin-4 production	5	0.000713
GO.0048525	Negative regulation of viral process	9	0.000725

GO.0031324	Negative regulation of cellular metabolic process	64	0.000729
GO.0042129	Regulation of t cell proliferation	11	0.000738
GO.0001912	Positive regulation of leukocyte mediated cytotoxicity	6	0.000745
GO.0050871	Positive regulation of b cell activation	8	0.000752
GO.0032846	Positive regulation of homeostatic process	12	0.000842
GO.0002708	Positive regulation of lymphocyte mediated immunity	8	0.000844
GO.0097300	Programmed necrotic cell death	6	0.000897
GO.0016043	Cellular component organization	112	0.000929
GO.0050707	Regulation of cytokine secretion	11	0.000976
GO.0002683	Negative regulation of immune system process	19	0.001
GO.0007346	Regulation of mitotic cell cycle	22	0.00102
GO.0010820	Positive regulation of t cell chemotaxis	4	0.00102
GO.0032386	Regulation of intracellular transport	25	0.0011
GO.0050714	Positive regulation of protein secretion	13	0.00115
GO.0048878	Chemical homeostasis	32	0.0012
GO.0051279	Regulation of release of sequestered calcium ion into cytosol	8	0.00129
GO.0031399	Regulation of protein modification process	49	0.00133
GO.0002685	Regulation of leukocyte migration	11	0.00145
GO.0032743	Positive regulation of interleukin-2 production	6	0.00148
GO.0045060	Negative thymic t cell selection	4	0.00152
GO.0048010	Vascular endothelial growth factor receptor signaling pathway	10	0.00154
GO.0071621	Granulocyte chemotaxis	7	0.00155
GO.0051301	Cell division	22	0.00156
GO.0071840	Cellular component organization or biogenesis	113	0.00156
GO.0032845	Negative regulation of homeostatic process	9	0.00158
GO.0032388	Positive regulation of intracellular transport	18	0.00161
GO.0050708	Regulation of protein secretion	19	0.00169
GO.0070661	Leukocyte proliferation	9	0.00171
GO.0009725	Response to hormone	32	0.00172
GO.0001816	Cytokine production	10	0.00174
GO.0006796	Phosphate-containing compound metabolic process	53	0.00184
GO.0002831	Regulation of response to biotic stimulus	14	0.00203
GO.0050793	Regulation of developmental process	59	0.00204
GO.0032479	Regulation of type i interferon production	10	0.00214
GO.0034612	Response to tumor necrosis factor	10	0.00214

GO.0007259	Jak-stat cascade	7	0.00216
GO.0071356	Cellular response to tumor necrosis factor	9	0.00233
GO.0009719	Response to endogenous stimulus	44	0.00245
GO.0006875	Cellular metal ion homeostasis	19	0.00251
GO.0097527	Necroptotic signaling pathway	3	0.00251
GO.0019725	Cellular homeostasis	26	0.00259
GO.0043434	Response to peptide hormone	20	0.00259
GO.0051272	Positive regulation of cellular component movement	18	0.0026
GO.0006301	Postreplication repair	7	0.00268
GO.0006897	Endocytosis	22	0.00275
GO.0007095	Mitotic g2 dna damage checkpoint	4	0.00288
GO.0019220	Regulation of phosphate metabolic process	46	0.00288
GO.0045086	Positive regulation of interleukin-2 biosynthetic process	4	0.00288
GO.0002690	Positive regulation of leukocyte chemotaxis	8	0.00289
GO.0042108	Positive regulation of cytokine biosynthetic process	7	0.00295
GO.0038083	Peptidyl-tyrosine autophosphorylation	6	0.00296
GO.0031334	Positive regulation of protein complex assembly	11	0.00305
GO.0055074	Calcium ion homeostasis	16	0.00305
GO.1903532	Positive regulation of secretion by cell	16	0.00305
GO.0007229	Integrin-mediated signaling pathway	8	0.00312
GO.1903901	Negative regulation of viral life cycle	8	0.00312
GO.0010605	Negative regulation of macromolecule metabolic process	61	0.00314
GO.0019882	Antigen processing and presentation	13	0.00314
GO.0002828	Regulation of type 2 immune response	5	0.00318
GO.0040017	Positive regulation of locomotion	18	0.00318
GO.0051047	Positive regulation of secretion	17	0.00318
GO.0071396	Cellular response to lipid	18	0.00318
GO.2000021	Regulation of ion homeostasis	12	0.0034
GO.0050801	Ion homeostasis	23	0.00341
GO.0045580	Regulation of t cell differentiation	9	0.00347
GO.0044699	Single-organism process	207	0.00352
GO.0051726	Regulation of cell cycle	33	0.00353
GO.0009891	Positive regulation of biosynthetic process	51	0.00368
GO.0050857	Positive regulation of antigen receptor-mediated signaling pathway	4	0.00373
GO.0070266	Necroptotic process	5	0.00375
GO.0031295	T cell costimulation	7	0.00386
GO.0046634	Regulation of alpha-beta t cell activation	7	0.00386
GO.0032103	Positive regulation of response to external stimulus	18	0.00395

GO.0044092	Negative regulation of molecular function	36	0.00411
GO.0009892	Negative regulation of metabolic process	66	0.00417
GO.2000107	Negative regulation of leukocyte apoptotic process	6	0.00421
GO.0002824	Positive regulation of adaptive immune response based on somatic recombination of immune receptors built from immunoglobulin superfamily domains	7	0.00423
GO.0007204	Positive regulation of cytosolic calcium ion concentration	11	0.0043
GO.0045937	Positive regulation of phosphate metabolic process	33	0.0043
GO.0003149	Membranous septum morphogenesis	3	0.00435
GO.0030335	Positive regulation of cell migration	17	0.00436
GO.0044774	Mitotic dna integrity checkpoint	9	0.00446
GO.0034138	Toll-like receptor 3 signaling pathway	8	0.00447
GO.0046651	Lymphocyte proliferation	8	0.00447
GO.0051049	Regulation of transport	50	0.00447
GO.0055082	Cellular chemical homeostasis	22	0.00468
GO.0046641	Positive regulation of alpha-beta t cell proliferation	4	0.00471
GO.1903530	Regulation of secretion by cell	24	0.00471
GO.0051928	Positive regulation of calcium ion transport	9	0.00508
GO.0034113	Heterotypic cell-cell adhesion	5	0.00511
GO.0002637	Regulation of immunoglobulin production	6	0.00517
GO.0019538	Protein metabolic process	94	0.00517
GO.0045071	Negative regulation of viral genome replication	6	0.00517
GO.0051173	Positive regulation of nitrogen compound metabolic process	50	0.00517
GO.0051222	Positive regulation of protein transport	19	0.00529
GO.0006874	Cellular calcium ion homeostasis	15	0.00538
GO.0051347	Positive regulation of transferase activity	23	0.0054
GO.0010557	Positive regulation of macromolecule biosynthetic process	47	0.00546
GO.0055065	Metal ion homeostasis	20	0.0057
GO.0046635	Positive regulation of alpha-beta t cell activation	6	0.00576
GO.0002639	Positive regulation of immunoglobulin production	5	0.00581
GO.0032735	Positive regulation of interleukin-12 production	5	0.00581
GO.0002700	Regulation of production of molecular mediator of immune response	8	0.00587
GO.0031579	Membrane raft organization	4	0.00587

GO.0051091	Positive regulation of sequence-specific dna binding transcription factor activity	13	0.00587
GO.0097305	Response to alcohol	16	0.0059
GO.0051248	Negative regulation of protein metabolic process	34	0.00616
GO.0002285	Lymphocyte activation involved in immune response	8	0.0063
GO.0045935	Positive regulation of nucleobase-containing compound metabolic process	48	0.00642
GO.0051046	Regulation of secretion	25	0.00646
GO.0001913	T cell mediated cytotoxicity	3	0.00674
GO.0019441	Tryptophan catabolic process to kynurenine	3	0.00674
GO.0050862	Positive regulation of t cell receptor signaling pathway	3	0.00674
GO.0031328	Positive regulation of cellular biosynthetic process	49	0.00691
GO.0033554	Cellular response to stress	47	0.00705
GO.0043154	Negative regulation of cysteine-type endopeptidase activity involved in apoptotic process	8	0.00723
GO.0034314	Arp2/3 complex-mediated actin nucleation	4	0.00727
GO.0045069	Regulation of viral genome replication	7	0.0075
GO.0031401	Positive regulation of protein modification process	34	0.00752
GO.0055080	Cation homeostasis	21	0.00757
GO.0042325	Regulation of phosphorylation	39	0.00763
GO.0045124	Regulation of bone resorption	5	0.00764
GO.0019985	Translesion synthesis	6	0.00769
GO.0033077	T cell differentiation in thymus	6	0.00769
GO.0001932	Regulation of protein phosphorylation	37	0.00774
GO.0051090	Regulation of sequence-specific dna binding transcription factor activity	17	0.00797
GO.0038095	Fc-epsilon receptor signaling pathway	11	0.00848
GO.0009165	Nucleotide biosynthetic process	12	0.00854
GO.0006271	Dna strand elongation involved in dna replication	5	0.00866
GO.0051209	Release of sequestered calcium ion into cytosol	5	0.00866
GO.0002274	Myeloid leukocyte activation	8	0.00869
GO.0009987	Cellular process	232	0.00872
GO.0045954	Positive regulation of natural killer cell mediated cytotoxicity	4	0.00872
GO.0048535	Lymph node development	4	0.00872
GO.0001818	Negative regulation of cytokine production	12	0.00918

GO.0001906	Cell killing	6	0.0093
GO.0006468	Protein phosphorylation	28	0.00943
GO.0045595	Regulation of cell differentiation	43	0.00947
GO.0051338	Regulation of transferase activity	30	0.00949
GO.0000082	G1/s transition of mitotic cell cycle	11	0.00953
GO.0031648	Protein destabilization	5	0.00975
GO.0002693	Positive regulation of cellular extravasation	3	0.00979
GO.0098771	Inorganic ion homeostasis	21	0.00983
GO.1903649	Regulation of cytoplasmic transport	19	0.00989
GO.0003366	Cell-matrix adhesion involved in ameboidal cell migration	2	0.01
GO.0045404	Positive regulation of interleukin-4 biosynthetic process	2	0.01
GO.0046631	Alpha-beta t cell activation	6	0.01
GO.0097049	Motor neuron apoptotic process	2	0.01
GO.2000451	Positive regulation of cd8-positive		
GO.0051128	Regulation of cellular component organization	57	0.0102
GO.0002573	Myeloid leukocyte differentiation	8	0.0103
GO.0002822	Regulation of adaptive immune response based on somatic recombination of immune receptors built from immunoglobulin superfamily domains	8	0.0103
GO.0006464	Cellular protein modification process	67	0.0103
GO.0043412	Macromolecule modification	70	0.0103
GO.0051603	Proteolysis involved in cellular protein catabolic process	20	0.0103
GO.2000514	Regulation of cd4-positive		
GO.0045786	Negative regulation of cell cycle	19	0.011
GO.0010564	Regulation of cell cycle process	21	0.0113
GO.0051092	Positive regulation of nf-kappab transcription factor activity	9	0.0119
GO.0070228	Regulation of lymphocyte apoptotic process	6	0.012
GO.0090630	Activation of gtpase activity	5	0.0122
GO.0044773	Mitotic dna damage checkpoint	8	0.0123
GO.0031570	Dna integrity checkpoint	10	0.0124
GO.0045861	Negative regulation of proteolysis	16	0.0129
GO.0048585	Negative regulation of response to stimulus	39	0.0131
GO.0001771	Immunological synapse formation	3	0.0135
GO.0010529	Negative regulation of transposition	3	0.0135
GO.0051495	Positive regulation of cytoskeleton organization	10	0.0136
GO.0032653	Regulation of interleukin-10 production	5	0.0137

GO.0019219	Regulation of nucleobase-containing compound metabolic process	87	0.014
GO.0035666	Trif-dependent toll-like receptor signaling pathway	7	0.0141
GO.0044772	Mitotic cell cycle phase transition	15	0.0147
GO.0045893	Positive regulation of transcription		
GO.2001235	Positive regulation of apoptotic signaling pathway	11	0.0148
GO.0006281	Dna repair	18	0.015
GO.0032269	Negative regulation of cellular protein metabolic process	31	0.015
GO.2000106	Regulation of leukocyte apoptotic process	7	0.015
GO.0034103	Regulation of tissue remodeling	6	0.0154
GO.0044257	Cellular protein catabolic process	20	0.0154
GO.1901990	Regulation of mitotic cell cycle phase transition	13	0.0156
GO.1901575	Organic substance catabolic process	43	0.0159
GO.0051171	Regulation of nitrogen compound metabolic process	92	0.016
GO.0050688	Regulation of defense response to virus	11	0.0166
GO.0051241	Negative regulation of multicellular organismal process	31	0.0168
GO.0000819	Sister chromatid segregation	7	0.017
GO.0016572	Histone phosphorylation	4	0.017
GO.0051130	Positive regulation of cellular component organization	35	0.017
GO.0030163	Protein catabolic process	21	0.0171
GO.0002664	Regulation of t cell tolerance induction	3	0.0179
GO.1901701	Cellular response to oxygen-containing compound	27	0.0182
GO.0010628	Positive regulation of gene expression	46	0.0188
GO.0034121	Regulation of toll-like receptor signaling pathway	5	0.0188
GO.0042098	T cell proliferation	5	0.0188
GO.0051270	Regulation of cellular component movement	25	0.0191
GO.0033273	Response to vitamin	7	0.0195
GO.0071363	Cellular response to growth factor stimulus	23	0.0196
GO.0043254	Regulation of protein complex assembly	14	0.0204
GO.0043113	Receptor clustering	5	0.0209
GO.0045670	Regulation of osteoclast differentiation	6	0.0212
GO.0016310	Phosphorylation	33	0.0213
GO.0046330	Positive regulation of jnk cascade	8	0.0215
GO.0030099	Myeloid cell differentiation	11	0.0226
GO.0071156	Regulation of cell cycle arrest	8	0.0227

GO.0006974	Cellular response to dna damage stimulus	24	0.0234
GO.0032611	Interleukin-1 beta production	3	0.0234
GO.0032740	Positive regulation of interleukin-17 production	3	0.0234
GO.0018193	Peptidyl-amino acid modification	28	0.0238
GO.0044267	Cellular protein metabolic process	79	0.0242
GO.1901698	Response to nitrogen compound	28	0.0246
GO.0001766	Membrane raft polarization	2	0.0249
GO.0001959	Regulation of cytokine-mediated signaling pathway	8	0.0249
GO.0043420	Anthranilate metabolic process	2	0.0249
GO.0045074	Regulation of interleukin-10 biosynthetic process	2	0.0249
GO.0046633	Alpha-beta t cell proliferation	2	0.0249
GO.1901293	Nucleoside phosphate biosynthetic process	11	0.0249
GO.0030593	Neutrophil chemotaxis	5	0.0251
GO.0040012	Regulation of locomotion	24	0.0253
GO.0043903	Regulation of symbiosis		
GO.0002761	Regulation of myeloid leukocyte differentiation	8	0.0261
GO.0034105	Positive regulation of tissue remodeling	4	0.0261
GO.0071158	Positive regulation of cell cycle arrest	7	0.0261
GO.0000077	Dna damage checkpoint	9	0.0263
GO.0010243	Response to organonitrogen compound	26	0.0269
GO.0043086	Negative regulation of catalytic activity	27	0.0273
GO.0050920	Regulation of chemotaxis	9	0.0275
GO.0000904	Cell morphogenesis involved in differentiation	23	0.0281
GO.0051276	Chromosome organization	28	0.0288
GO.0050855	Regulation of b cell receptor signaling pathway	3	0.0291
GO.0051254	Positive regulation of rna metabolic process	41	0.0294
GO.0033280	Response to vitamin d	4	0.0296
GO.0033674	Positive regulation of kinase activity	18	0.0296
GO.0042036	Negative regulation of cytokine biosynthetic process	4	0.0296
GO.2000516	Positive regulation of cd4-positive		
GO.0042327	Positive regulation of phosphorylation	27	0.0298
GO.0045582	Positive regulation of t cell differentiation	6	0.0298
GO.0050792	Regulation of viral process	10	0.0315
GO.1903827	Regulation of cellular protein localization	19	0.0315
GO.2000026	Regulation of multicellular organismal development	42	0.0315
GO.0044248	Cellular catabolic process	40	0.0326

GO.0050856	Regulation of t cell receptor signaling pathway	4	0.0334
GO.0060142	Regulation of syncytium formation by plasma membrane fusion	4	0.0334
GO.0071353	Cellular response to interleukin-4	4	0.0334
GO.0008284	Positive regulation of cell proliferation	26	0.0336
GO.0001934	Positive regulation of protein phosphorylation	26	0.0342
GO.0032501	Multicellular organismal process	119	0.0347
GO.0061564	Axon development	19	0.0349
GO.0043950	Positive regulation of camp-mediated signaling	3	0.0357
GO.0045730	Respiratory burst	3	0.0357
GO.0045780	Positive regulation of bone resorption	3	0.0357
GO.0090026	Positive regulation of monocyte chemotaxis	3	0.0357
GO.0090068	Positive regulation of cell cycle process	12	0.0365
GO.0000280	Nuclear division	17	0.0366
GO.0007162	Negative regulation of cell adhesion	11	0.037
GO.0050690	Regulation of defense response to virus by virus	4	0.0376
GO.0002223	Stimulatory c-type lectin receptor signaling pathway	8	0.0378
GO.0050848	Regulation of calcium-mediated signaling	5	0.0385
GO.0051056	Regulation of small gtpase mediated signal transduction	11	0.0413
GO.0007568	Aging	12	0.0415
GO.0045859	Regulation of protein kinase activity	23	0.0415
GO.2000145	Regulation of cell motility	22	0.0419
GO.0050850	Positive regulation of calcium-mediated signaling	4	0.0425
GO.0000070	Mitotic sister chromatid segregation	6	0.0432
GO.0002827	Positive regulation of t-helper 1 type immune response	3	0.0432
GO.0002830	Positive regulation of type 2 immune response	3	0.0432
GO.0003150	Muscular septum morphogenesis	2	0.0432
GO.0006517	Protein deglycosylation	2	0.0432
GO.0006878	Cellular copper ion homeostasis	3	0.0432
GO.0019805	Quinolinate biosynthetic process	2	0.0432
GO.0030334	Regulation of cell migration	21	0.0432
GO.0032070	Regulation of deoxyribonuclease activity	2	0.0432
GO.0033634	Positive regulation of cell-cell adhesion mediated by integrin	2	0.0432
GO.0034354	De novo nad biosynthetic process from tryptophan	2	0.0432

GO.0038110	Interleukin-2-mediated signaling pathway	2	0.0432
GO.0043306	Positive regulation of mast cell degranulation	3	0.0432
GO.0051701	Interaction with host	8	0.0432
GO.0055099	Response to high density lipoprotein particle	2	0.0432
GO.0070269	Pyroptosis	2	0.0432
GO.0071260	Cellular response to mechanical stimulus	6	0.0432
GO.0071352	Cellular response to interleukin-2	2	0.0432
GO.0010389	Regulation of g2/m transition of mitotic cell cycle	5	0.0444
GO.0043549	Regulation of kinase activity	24	0.0446
GO.0007059	Chromosome segregation	10	0.0457
GO.0016192	Vesicle-mediated transport	33	0.0458
GO.0009057	Macromolecule catabolic process	28	0.046
GO.0044707	Single-multicellular organism process	114	0.0477
GO.1903651	Positive regulation of cytoplasmic transport	12	0.0482
GO.0006259	Dna metabolic process	23	0.0487
GO.0000723	Telomere maintenance	6	0.0489

Table S3.2. KEGG pathway enrichment in upregulated group of RA versus normalcy

Pathway ID	Pathway description	Observed gene count	False discovery rate
4064	NF-kappa B signaling pathway	18	9.00E-12
4060	Cytokine-cytokine receptor interaction	27	6.54E-11
4062	Chemokine signaling pathway	21	1.62E-09
5162	Measles	18	2.06E-09
5166	HTLV-I infection	24	2.84E-09
4620	Toll-like receptor signaling pathway	15	2.19E-08
5340	Primary immunodeficiency	10	2.19E-08
4650	Natural killer cell mediated cytotoxicity	16	3.79E-08
4640	Hematopoietic cell lineage	12	1.66E-06
4380	Osteoclast differentiation	14	1.90E-06
5164	Influenza A	16	2.13E-06
5168	Herpes simplex infection	16	3.76E-06
4662	B cell receptor signaling pathway	10	1.09E-05
5140	Leishmaniasis	10	1.09E-05
4670	Leukocyte transendothelial migration	12	2.24E-05
4672	Intestinal immune network for IgA production	8	2.24E-05
4660	T cell receptor signaling pathway	11	3.33E-05
5169	Epstein-Barr virus infection	15	3.98E-05
5323	Rheumatoid arthritis	10	5.68E-05
5202	Transcriptional misregulation in cancer	13	0.000169
5152	Tuberculosis	13	0.000237
4114	Oocyte meiosis	10	0.000326
4668	TNF signaling pathway	10	0.000339
4666	Fc gamma R-mediated phagocytosis	9	0.000393
5133	Pertussis	8	0.000453
4514	Cell adhesion molecules (CAMs)	11	0.000507
5142	Chagas disease (American trypanosomiasis)	9	0.000764
4611	Platelet activation	10	0.00122
4630	Jak-STAT signaling pathway	11	0.0014
4210	Apoptosis	8	0.00155
4115	p53 signaling pathway	7	0.00185
4612	Antigen processing and presentation	7	0.00185
4664	Fc epsilon RI signaling pathway	7	0.00185
5145	Toxoplasmosis	9	0.00199
5161	Hepatitis B	10	0.00218
5143	African trypanosomiasis	5	0.00259
4750	Inflammatory mediator regulation of TRP channels	8	0.00273
5330	Allograft rejection	5	0.00284
5332	Graft-versus-host disease	5	0.00363
4914	Progesterone-mediated oocyte maturation	7	0.00462

920	Sulfur metabolism	3	0.00477
5132	Salmonella infection	7	0.00511
4940	Type I diabetes mellitus	5	0.00529
4970	Salivary secretion	7	0.00529
3050	Proteasome	5	0.00723
4270	Vascular smooth muscle contraction	8	0.00836
5144	Malaria	5	0.00942
4810	Regulation of actin cytoskeleton	11	0.0105
4110	Cell cycle	8	0.0109
4916	Melanogenesis	7	0.0115
5320	Autoimmune thyroid disease	5	0.0115
5110	Vibrio cholerae infection	5	0.0135
4010	MAPK signaling pathway	12	0.0139
5160	Hepatitis C	8	0.014
5416	Viral myocarditis	5	0.0179
4621	NOD-like receptor signaling pathway	5	0.019
3030	DNA replication	4	0.0192
4145	Phagosome	8	0.0245
4720	Long-term potentiation	5	0.0298
5203	Viral carcinogenesis	9	0.0318
3430	Mismatch repair	3	0.0396
3420	Nucleotide excision repair	4	0.047

Table S3.3. GO pathway enrichment in downregulated group of RA versus normalcy

Pathway ID	Pathway description	Observed gene count	False discovery rate
GO.0051246	Regulation of protein metabolic process	47	2.89E-07
GO.0071310	Cellular response to organic substance	40	3.16E-07
GO.0051240	Positive regulation of multicellular organismal process	33	7.24E-07
GO.0031325	Positive regulation of cellular metabolic process	49	8.06E-07
GO.0070848	Response to growth factor	23	8.06E-07
GO.0032268	Regulation of cellular protein metabolic process	43	9.60E-07
GO.0001568	Blood vessel development	19	1.14E-06
GO.0010628	Positive regulation of gene expression	36	1.14E-06
GO.0071363	Cellular response to growth factor stimulus	22	1.28E-06
GO.0001944	Vasculature development	19	2.01E-06
GO.0051173	Positive regulation of nitrogen compound metabolic process	36	2.02E-06
GO.0051254	Positive regulation of rna metabolic process	33	2.02E-06
GO.0070887	Cellular response to chemical stimulus	42	2.02E-06
GO.0071495	Cellular response to endogenous stimulus	27	2.04E-06
GO.0000122	Negative regulation of transcription from rna polymerase ii promoter	23	3.56E-06
GO.0051239	Regulation of multicellular organismal process	42	3.57E-06
GO.0048513	Organ development	45	3.85E-06
GO.0050793	Regulation of developmental process	39	3.85E-06
GO.0080090	Regulation of primary metabolic process	69	3.85E-06
GO.0007167	Enzyme linked receptor protein signaling pathway	25	4.49E-06
GO.0045935	Positive regulation of nucleobase-containing compound metabolic process	34	5.66E-06
GO.0010558	Negative regulation of macromolecule biosynthetic process	30	5.87E-06
GO.0071559	Response to transforming growth factor beta	12	6.04E-06
GO.0010604	Positive regulation of macromolecule metabolic process	44	6.92E-06
GO.0048523	Negative regulation of cellular process	56	6.92E-06
GO.0009893	Positive regulation of metabolic process	52	6.93E-06
GO.2000113	Negative regulation of cellular macromolecule biosynthetic process	29	7.43E-06
GO.0010033	Response to organic substance	42	7.78E-06
GO.0048514	Blood vessel morphogenesis	16	7.78E-06
GO.0031328	Positive regulation of cellular biosynthetic process	34	8.57E-06
GO.0019222	Regulation of metabolic process	75	8.78E-06
GO.0060255	Regulation of macromolecule metabolic process	67	1.05E-05
GO.0001655	Urogenital system development	14	1.16E-05
GO.0001822	Kidney development	13	1.16E-05
GO.0010605	Negative regulation of macromolecule metabolic process	39	1.16E-05

GO.0045934	Negative regulation of nucleobase-containing compound metabolic process	29	1.16E-05
GO.0045893	Positive regulation of transcription		
GO.0010557	Positive regulation of macromolecule biosynthetic process	32	1.52E-05
GO.0051094	Positive regulation of developmental process	26	1.60E-05
GO.0051253	Negative regulation of rna metabolic process	27	1.70E-05
GO.0072001	Renal system development	13	1.70E-05
GO.0051591	Response to camp	9	1.73E-05
GO.0031323	Regulation of cellular metabolic process	68	1.78E-05
GO.0048522	Positive regulation of cellular process	58	1.78E-05
GO.0045892	Negative regulation of transcription		
GO.2000112	Regulation of cellular macromolecule biosynthetic process	52	1.94E-05
GO.0031326	Regulation of cellular biosynthetic process	54	2.38E-05
GO.1903507	Negative regulation of nucleic acid-templated transcription	26	2.38E-05
GO.0031327	Negative regulation of cellular biosynthetic process	29	2.43E-05
GO.0048518	Positive regulation of biological process	63	2.43E-05
GO.0071560	Cellular response to transforming growth factor beta stimulus	11	2.45E-05
GO.0031324	Negative regulation of cellular metabolic process	38	2.72E-05
GO.0051172	Negative regulation of nitrogen compound metabolic process	29	3.08E-05
GO.0014074	Response to purine-containing compound	10	3.68E-05
GO.0051270	Regulation of cellular component movement	20	3.92E-05
GO.0061061	Muscle structure development	16	3.98E-05
GO.0072358	Cardiovascular system development	21	3.98E-05
GO.0072359	Circulatory system development	21	3.98E-05
GO.0009892	Negative regulation of metabolic process	40	4.41E-05
GO.0071248	Cellular response to metal ion	9	4.83E-05
GO.0010468	Regulation of gene expression	53	5.81E-05
GO.0010941	Regulation of cell death	29	5.82E-05
GO.0030334	Regulation of cell migration	18	5.82E-05
GO.0032501	Multicellular organismal process	67	5.99E-05
GO.0048608	Reproductive structure development	15	5.99E-05
GO.0001503	Ossification	12	6.11E-05
GO.0009889	Regulation of biosynthetic process	53	6.24E-05
GO.0001657	Ureteric bud development	8	6.41E-05
GO.0061458	Reproductive system development	15	6.41E-05
GO.0007275	Multicellular organismal development	54	6.65E-05
GO.0009719	Response to endogenous stimulus	28	6.65E-05
GO.0048519	Negative regulation of biological process	55	7.52E-05
GO.0040012	Regulation of locomotion	19	8.07E-05
GO.0001823	Mesonephros development	8	8.28E-05
GO.0048731	System development	49	8.29E-05

GO.0051171	Regulation of nitrogen compound metabolic process	53	8.75E-05
GO.0009653	Anatomical structure morphogenesis	35	9.26E-05
GO.0007179	Transforming growth factor beta receptor signaling pathway	9	0.000123
GO.0009888	Tissue development	29	0.000123
GO.0042981	Regulation of apoptotic process	27	0.000134
GO.0044707	Single-multicellular organism process	64	0.000134
GO.0006417	Regulation of translation	12	0.00015
GO.0007166	Cell surface receptor signaling pathway	34	0.000171
GO.0060429	Epithelium development	22	0.000186
GO.0060548	Negative regulation of cell death	21	0.000215
GO.0048511	Rhythmic process	12	0.00022
GO.0010629	Negative regulation of gene expression	27	0.00024
GO.0032870	Cellular response to hormone stimulus	16	0.000252
GO.2000026	Regulation of multicellular organismal development	28	0.000276
GO.0045168	Cell-cell signaling involved in cell fate commitment	5	0.000321
GO.0032970	Regulation of actin filament-based process	12	0.000325
GO.0048145	Regulation of fibroblast proliferation	7	0.000325
GO.0048660	Regulation of smooth muscle cell proliferation	7	0.000325
GO.0009628	Response to abiotic stimulus	23	0.000379
GO.0042127	Regulation of cell proliferation	27	0.000379
GO.0051726	Regulation of cell cycle	21	0.000384
GO.0006355	Regulation of transcription		
GO.0002009	Morphogenesis of an epithelium	14	0.000452
GO.0007155	Cell adhesion	21	0.000461
GO.0045597	Positive regulation of cell differentiation	19	0.000461
GO.0048729	Tissue morphogenesis	16	0.000461
GO.0009968	Negative regulation of signal transduction	22	0.000486
GO.0071504	Cellular response to heparin	3	0.000489
GO.0007399	Nervous system development	32	0.000513
GO.0042063	Gliogenesis	9	0.000513
GO.0051493	Regulation of cytoskeleton organization	13	0.000513
GO.0042221	Response to chemical	48	0.000536
GO.0038095	Fc-epsilon receptor signaling pathway	9	0.000558
GO.0072073	Kidney epithelium development	8	0.00059
GO.0009314	Response to radiation	14	0.000616
GO.0010001	Glial cell differentiation	8	0.000616
GO.0048646	Anatomical structure formation involved in morphogenesis	21	0.000619
GO.0016477	Cell migration	18	0.000641
GO.0000956	Nuclear-transcribed mrna catabolic process	9	0.000677
GO.0038093	Fc receptor signaling pathway	10	0.000749
GO.0043066	Negative regulation of apoptotic process	19	0.000766

GO.0010608	Posttranscriptional regulation of gene expression	12	0.000795
GO.0048732	Gland development	13	0.000801
GO.0007169	Transmembrane receptor protein tyrosine kinase signaling pathway	17	0.000909
GO.0007417	Central nervous system development	19	0.000909
GO.0010464	Regulation of mesenchymal cell proliferation	5	0.000953
GO.0043065	Positive regulation of apoptotic process	15	0.000979
GO.0006357	Regulation of transcription from rna polymerase ii promoter	28	0.000989
GO.0034097	Response to cytokine	16	0.00101
GO.1901698	Response to nitrogen compound	19	0.00101
GO.0043523	Regulation of neuron apoptotic process	9	0.0011
GO.0048598	Embryonic morphogenesis	15	0.00115
GO.0014070	Response to organic cyclic compound	18	0.00124
GO.0009612	Response to mechanical stimulus	9	0.00139
GO.0008285	Negative regulation of cell proliferation	16	0.00147
GO.0035295	Tube development	15	0.00159
GO.0032956	Regulation of actin cytoskeleton organization	10	0.0016
GO.0051252	Regulation of rna metabolic process	44	0.00164
GO.0051674	Localization of cell	18	0.00173
GO.0071277	Cellular response to calcium ion	5	0.00177
GO.0051128	Regulation of cellular component organization	32	0.0019
GO.0030510	Regulation of bmp signaling pathway	6	0.00192
GO.0042325	Regulation of phosphorylation	23	0.00192
GO.0001932	Regulation of protein phosphorylation	22	0.00196
GO.0022414	Reproductive process	22	0.00196
GO.0090092	Regulation of transmembrane receptor protein serine/threonine kinase signaling pathway	9	0.00196
GO.0045944	Positive regulation of transcription from rna polymerase ii promoter	20	0.00199
GO.0008283	Cell proliferation	16	0.002
GO.2000826	Regulation of heart morphogenesis	4	0.00207
GO.0001649	Osteoblast differentiation	7	0.00214
GO.0019219	Regulation of nucleobase-containing compound metabolic process	46	0.00214
GO.0045595	Regulation of cell differentiation	25	0.00214
GO.0009605	Response to external stimulus	29	0.00241
GO.0048856	Anatomical structure development	49	0.00241
GO.0031399	Regulation of protein modification process	26	0.00261
GO.0051248	Negative regulation of protein metabolic process	20	0.00266
GO.0000288	Nuclear-transcribed mrna catabolic process		
GO.0007178	Transmembrane receptor protein serine/threonine kinase signaling pathway	9	0.00276
GO.0010467	Gene expression	48	0.00295
GO.0007517	Muscle organ development	10	0.003
GO.0048583	Regulation of response to stimulus	42	0.003

GO.0019220	Regulation of phosphate metabolic process	25	0.00302
GO.0033993	Response to lipid	17	0.00302
GO.0009790	Embryo development	19	0.00307
GO.0044702	Single organism reproductive process	20	0.00342
GO.0060395	Smad protein signal transduction	5	0.00342
GO.0048146	Positive regulation of fibroblast proliferation	5	0.00366
GO.0048844	Artery morphogenesis	5	0.00366
GO.0060675	Ureteric bud morphogenesis	5	0.00366
GO.0010038	Response to metal ion	10	0.00374
GO.0071496	Cellular response to external stimulus	10	0.00394
GO.0010035	Response to inorganic substance	12	0.00398
GO.0031128	Developmental induction	4	0.00398
GO.0090287	Regulation of cellular response to growth factor stimulus	9	0.00398
GO.0007420	Brain development	15	0.0044
GO.1901654	Response to ketone	7	0.00444
GO.0048662	Negative regulation of smooth muscle cell proliferation	4	0.0045
GO.0071396	Cellular response to lipid	11	0.0046
GO.1901722	Regulation of cell proliferation involved in kidney development	3	0.00462
GO.0032879	Regulation of localization	32	0.00463
GO.0006402	Mrna catabolic process	8	0.00517
GO.0048015	Phosphatidylinositol-mediated signaling	7	0.00518
GO.0071383	Cellular response to steroid hormone stimulus	7	0.00518
GO.0042490	Mechanoreceptor differentiation	5	0.00519
GO.0040011	Locomotion	21	0.00521
GO.0044238	Primary metabolic process	80	0.00574
GO.0014033	Neural crest cell differentiation	5	0.00597
GO.0051492	Regulation of stress fiber assembly	5	0.00597
GO.0030154	Cell differentiation	39	0.00599
GO.0051704	Multi-organism process	31	0.00615
GO.0007548	Sex differentiation	9	0.00633
GO.0060840	Artery development	5	0.00635
GO.0098602	Single organism cell adhesion	12	0.00676
GO.0048585	Negative regulation of response to stimulus	22	0.00691
GO.0050679	Positive regulation of epithelial cell proliferation	7	0.00763
GO.0043583	Ear development	8	0.00774
GO.0035556	Intracellular signal transduction	27	0.00828
GO.0060322	Head development	15	0.00835
GO.0010243	Response to organonitrogen compound	16	0.00836
GO.0023051	Regulation of signaling	35	0.00859
GO.0032269	Negative regulation of cellular protein metabolic process	18	0.0086
GO.0007173	Epidermal growth factor receptor signaling pathway	8	0.00863

GO.0045667	Regulation of osteoblast differentiation	6	0.00863
GO.0043170	Macromolecule metabolic process	71	0.00869
GO.0072176	Nephric duct development	3	0.0088
GO.1903055	Positive regulation of extracellular matrix organization	3	0.0088
GO.0043009	Chordate embryonic development	14	0.00885
GO.0022612	Gland morphogenesis	6	0.0089
GO.0051145	Smooth muscle cell differentiation	4	0.00915
GO.0051496	Positive regulation of stress fiber assembly	4	0.00915
GO.0006807	Nitrogen compound metabolic process	58	0.00941
GO.0060197	Cloacal septation	2	0.0097
GO.0072190	Ureter urothelium development	2	0.0097
GO.0072191	Ureter smooth muscle development	2	0.0097
GO.0072193	Ureter smooth muscle cell differentiation	2	0.0097
GO.0072197	Ureter morphogenesis	2	0.0097
GO.0016070	Rna metabolic process	42	0.0101
GO.0030224	Monocyte differentiation	3	0.0101
GO.0032502	Developmental process	51	0.0101
GO.0045995	Regulation of embryonic development	6	0.0101
GO.0051592	Response to calcium ion	6	0.0101
GO.1900153	Positive regulation of nuclear-transcribed mrna catabolic process		
GO.1901215	Negative regulation of neuron death	7	0.0101
GO.0040007	Growth	11	0.0104
GO.0044089	Positive regulation of cellular component biogenesis	10	0.0104
GO.0051271	Negative regulation of cellular component movement	8	0.0104
GO.2000736	Regulation of stem cell differentiation	6	0.0104
GO.0003007	Heart morphogenesis	8	0.0107
GO.0009058	Biosynthetic process	52	0.011
GO.0034641	Cellular nitrogen compound metabolic process	55	0.0112
GO.0042493	Response to drug	11	0.0118
GO.2000136	Regulation of cell proliferation involved in heart morphogenesis	3	0.0118
GO.0044260	Cellular macromolecule metabolic process	66	0.0121
GO.0051896	Regulation of protein kinase b signaling	6	0.0123
GO.0090184	Positive regulation of kidney development	4	0.0123
GO.0007422	Peripheral nervous system development	5	0.0129
GO.0014013	Regulation of gliogenesis	5	0.0129
GO.0048011	Neurotrophin trk receptor signaling pathway	9	0.0129
GO.0048869	Cellular developmental process	39	0.0129
GO.1901576	Organic substance biosynthetic process	51	0.0129
GO.0030850	Prostate gland development	4	0.0132
GO.0032270	Positive regulation of cellular protein metabolic process	21	0.0132
GO.0070555	Response to interleukin-1	5	0.0134

GO.0065009	Regulation of molecular function	34	0.0136
GO.0072012	Glomerulus vasculature development	3	0.0136
GO.0022008	Neurogenesis	22	0.0137
GO.0002040	Sprouting angiogenesis	4	0.0141
GO.0030198	Extracellular matrix organization	10	0.0141
GO.0016337	Single organismal cell-cell adhesion	11	0.0142
GO.0044767	Single-organism developmental process	50	0.0145
GO.0051893	Regulation of focal adhesion assembly	4	0.0151
GO.0060485	Mesenchyme development	7	0.0151
GO.0045165	Cell fate commitment	8	0.0158
GO.0001656	Metanephros development	5	0.0161
GO.0035272	Exocrine system development	4	0.0161
GO.0035844	Cloaca development	2	0.0161
GO.0040013	Negative regulation of locomotion	8	0.0161
GO.0060235	Lens induction in camera-type eye	2	0.0161
GO.0051179	Localization	47	0.0171
GO.0032774	Rna biosynthetic process	35	0.0173
GO.0048839	Inner ear development	7	0.0173
GO.0009966	Regulation of signal transduction	31	0.0175
GO.0010646	Regulation of cell communication	35	0.0175
GO.0071453	Cellular response to oxygen levels	6	0.0176
GO.0030324	Lung development	7	0.0181
GO.0009725	Response to hormone	16	0.0182
GO.0044085	Cellular component biogenesis	27	0.0183
GO.0009991	Response to extracellular stimulus	11	0.0185
GO.0009059	Macromolecule biosynthetic process	43	0.0188
GO.0019438	Aromatic compound biosynthetic process	38	0.019
GO.0009887	Organ morphogenesis	16	0.0196
GO.0050794	Regulation of cellular process	81	0.0197
GO.0072273	Metanephric nephron morphogenesis	3	0.0197
GO.0017038	Protein import	6	0.0199
GO.0030323	Respiratory tube development	7	0.0199
GO.0043524	Negative regulation of neuron apoptotic process	6	0.0199
GO.0065008	Regulation of biological quality	36	0.0199
GO.0006366	Transcription from rna polymerase ii promoter	15	0.0201
GO.0019538	Protein metabolic process	44	0.0207
GO.0043525	Positive regulation of neuron apoptotic process	4	0.0207
GO.0060711	Labyrinthine layer development	4	0.0207
GO.0044344	Cellular response to fibroblast growth factor stimulus	7	0.0209
GO.0007219	Notch signaling pathway	6	0.0212
GO.0044403	Symbiosis		
GO.1903706	Regulation of hemopoiesis	9	0.0216
GO.0050789	Regulation of biological process	83	0.0221
GO.0097305	Response to alcohol	9	0.0221
GO.0006468	Protein phosphorylation	15	0.0224

GO.0008284	Positive regulation of cell proliferation	15	0.0224
GO.0019439	Aromatic compound catabolic process	10	0.0224
GO.0030336	Negative regulation of cell migration	7	0.0224
GO.0040008	Regulation of growth	13	0.0226
GO.0010891	Negative regulation of sequestering of triglyceride	2	0.0231
GO.0016032	Viral process	14	0.0231
GO.0034654	Nucleobase-containing compound biosynthetic process	37	0.0231
GO.0042692	Muscle cell differentiation	8	0.0231
GO.0061009	Common bile duct development	2	0.0231
GO.0061046	Regulation of branching involved in lung morphogenesis	2	0.0231
GO.0044237	Cellular metabolic process	76	0.0237
GO.0044708	Single-organism behavior	10	0.0237
GO.0006084	Acetyl-coa metabolic process	3	0.0239
GO.0043488	Regulation of mrna stability	4	0.0241
GO.0001892	Embryonic placenta development	5	0.0245
GO.0048589	Developmental growth	9	0.0245
GO.0034504	Protein localization to nucleus	6	0.0249
GO.0044249	Cellular biosynthetic process	49	0.025
GO.0008406	Gonad development	7	0.0252
GO.0010634	Positive regulation of epithelial cell migration	5	0.0252
GO.0014032	Neural crest cell development	4	0.0252
GO.0051247	Positive regulation of protein metabolic process	21	0.0252
GO.0060113	Inner ear receptor cell differentiation	4	0.0252
GO.0071347	Cellular response to interleukin-1	4	0.0252
GO.0000165	Mapk cascade	7	0.0258
GO.0007623	Circadian rhythm	6	0.026
GO.0044087	Regulation of cellular component biogenesis	13	0.026
GO.0090304	Nucleic acid metabolic process	44	0.026
GO.0044271	Cellular nitrogen compound biosynthetic process	40	0.0263
GO.1901360	Organic cyclic compound metabolic process	51	0.0263
GO.0045685	Regulation of glial cell differentiation	4	0.0264
GO.0048545	Response to steroid hormone	10	0.0264
GO.0048546	Digestive tract morphogenesis	4	0.0264
GO.0009416	Response to light stimulus	9	0.0276
GO.0098609	Cell-cell adhesion	12	0.0276
GO.0045137	Development of primary sexual characteristics	7	0.0281
GO.0000289	Nuclear-transcribed mrna poly(a) tail shortening	3	0.0287
GO.0001890	Placenta development	6	0.0291
GO.0030155	Regulation of cell adhesion	12	0.0301
GO.0042327	Positive regulation of phosphorylation	15	0.0302
GO.0000904	Cell morphogenesis involved in differentiation	13	0.0305
GO.0014909	Smooth muscle cell migration	2	0.0305
GO.0030335	Positive regulation of cell migration	9	0.0305
GO.0045596	Negative regulation of cell differentiation	13	0.0305

GO.0072201	Negative regulation of mesenchymal cell proliferation	2	0.0305
GO.1901724	Positive regulation of cell proliferation involved in kidney development	2	0.0305
GO.0006928	Movement of cell or subcellular component	20	0.0306
GO.0050678	Regulation of epithelial cell proliferation	8	0.0306
GO.1901362	Organic cyclic compound biosynthetic process	38	0.0307
GO.0001933	Negative regulation of protein phosphorylation	9	0.0309
GO.0010762	Regulation of fibroblast migration	3	0.0309
GO.0021885	Forebrain cell migration	4	0.0309
GO.0051716	Cellular response to stimulus	58	0.0309
GO.0060740	Prostate gland epithelium morphogenesis	3	0.0309
GO.0072215	Regulation of metanephros development	3	0.0309
GO.1901700	Response to oxygen-containing compound	20	0.0309
GO.0006605	Protein targeting	10	0.0312
GO.0030900	Forebrain development	9	0.0314
GO.0031400	Negative regulation of protein modification process	12	0.0314
GO.0048699	Generation of neurons	20	0.0318
GO.0043618	Regulation of transcription from rna polymerase ii promoter in response to stress	4	0.0321
GO.0042698	Ovulation cycle	5	0.0329
GO.0051170	Nuclear import	5	0.0329
GO.0060284	Regulation of cell development	14	0.0331
GO.0003151	Outflow tract morphogenesis	4	0.0332
GO.0003338	Metanephros morphogenesis	3	0.0332
GO.0030855	Epithelial cell differentiation	11	0.0332
GO.0031589	Cell-substrate adhesion	6	0.0332
GO.0043401	Steroid hormone mediated signaling pathway	4	0.0332
GO.0045936	Negative regulation of phosphate metabolic process	11	0.0332
GO.0055025	Positive regulation of cardiac muscle tissue development	3	0.0332
GO.1901361	Organic cyclic compound catabolic process	10	0.0332
GO.2000027	Regulation of organ morphogenesis	6	0.0332
GO.2001022	Positive regulation of response to dna damage stimulus	4	0.0332
GO.0051495	Positive regulation of cytoskeleton organization	6	0.0337
GO.0090090	Negative regulation of canonical wnt signaling pathway	6	0.0337
GO.0034645	Cellular macromolecule biosynthetic process	41	0.0347
GO.0050790	Regulation of catalytic activity	28	0.035
GO.0060537	Muscle tissue development	8	0.035
GO.0060541	Respiratory system development	7	0.0353
GO.0048147	Negative regulation of fibroblast proliferation	3	0.0361
GO.0061037	Negative regulation of cartilage development	3	0.0361

GO.0051052	Regulation of dna metabolic process	8	0.0362
GO.0071702	Organic substance transport	25	0.0362
GO.0072594	Establishment of protein localization to organelle	9	0.0362
GO.0045859	Regulation of protein kinase activity	13	0.0375
GO.0007154	Cell communication	51	0.0376
GO.0031668	Cellular response to extracellular stimulus	7	0.0376
GO.0032504	Multicellular organism reproduction	14	0.0376
GO.0032808	Lacrimal gland development	2	0.0376
GO.0045937	Positive regulation of phosphate metabolic process	16	0.0376
GO.0046942	Carboxylic acid transport	7	0.0376
GO.0048709	Oligodendrocyte differentiation	4	0.0376
GO.0060687	Regulation of branching involved in prostate gland morphogenesis	2	0.0376
GO.0072124	Regulation of glomerular mesangial cell proliferation	2	0.0376
GO.0072205	Metanephric collecting duct development	2	0.0376
GO.0071704	Organic substance metabolic process	77	0.0378
GO.0045687	Positive regulation of glial cell differentiation	3	0.0381
GO.0048713	Regulation of oligodendrocyte differentiation	3	0.0381
GO.0060512	Prostate gland morphogenesis	3	0.0381
GO.0021915	Neural tube development	6	0.0384
GO.0035239	Tube morphogenesis	9	0.0388
GO.0003008	System process	23	0.0389
GO.0060563	Neuroepithelial cell differentiation	4	0.0389
GO.0051090	Regulation of sequence-specific dna binding transcription factor activity	9	0.0399
GO.0022607	Cellular component assembly	24	0.0403
GO.0048568	Embryonic organ development	10	0.0406
GO.0002065	Columnar/cuboidal epithelial cell differentiation	5	0.0408
GO.0072078	Nephron tubule morphogenesis	4	0.0408
GO.0003156	Regulation of organ formation	3	0.041
GO.0006950	Response to stress	38	0.041
GO.0070849	Response to epidermal growth factor	3	0.041
GO.0071345	Cellular response to cytokine stimulus	11	0.0414
GO.0070482	Response to oxygen levels	8	0.0418
GO.0006351	Transcription		
GO.0072028	Nephron morphogenesis	4	0.0424
GO.0006725	Cellular aromatic compound metabolic process	48	0.0425
GO.0001701	In utero embryonic development	9	0.0426
GO.0008543	Fibroblast growth factor receptor signaling pathway	6	0.0429
GO.0033036	Macromolecule localization	26	0.0439
GO.0072210	Metanephric nephron development	3	0.0441
GO.0043407	Negative regulation of map kinase activity	4	0.0443
GO.0001101	Response to acid chemical	8	0.0446
GO.0046660	Female sex differentiation	5	0.0446

GO.0033365	Protein localization to organelle	11	0.0449
GO.0042447	Hormone catabolic process	2	0.0451
GO.0071455	Cellular response to hyperoxia	2	0.0451
GO.0072282	Metanephric nephron tubule morphogenesis	2	0.0451
GO.0001525	Angiogenesis	8	0.0457
GO.0001934	Positive regulation of protein phosphorylation	14	0.0457
GO.0002761	Regulation of myeloid leukocyte differentiation	5	0.0457
GO.0071229	Cellular response to acid chemical	6	0.0457
GO.1901564	Organonitrogen compound metabolic process	22	0.0457
GO.1901701	Cellular response to oxygen-containing compound	14	0.0457
GO.0048762	Mesenchymal cell differentiation	5	0.047
GO.0060674	Placenta blood vessel development	3	0.047
GO.2000648	Positive regulation of stem cell proliferation	4	0.0478
GO.0033043	Regulation of organelle organization	17	0.0493
GO.0065007	Biological regulation	83	0.0493
GO.0003279	Cardiac septum development	4	0.05
GO.0071456	Cellular response to hypoxia	5	0.05

Table S3.4. KEGG pathway enrichment in downregulated group of RA versus normalcy

Pathway ID	Pathway description	Observed gene count	False discovery rate
4010	MAPK signaling pathway	13	1.83E-05
4380	Osteoclast differentiation	9	5.90E-05
5200	Pathways in cancer	12	0.000589
4151	PI3K-Akt signaling pathway	11	0.00378
4915	Estrogen signaling pathway	6	0.00437
4152	AMPK signaling pathway	6	0.0141
4012	ErbB signaling pathway	5	0.0173
5166	HTLV-I infection	8	0.0235
61	Fatty acid biosynthesis	2	0.0267
4668	TNF signaling pathway	5	0.0359
5031	Amphetamine addiction	4	0.0359
5205	Proteoglycans in cancer	7	0.0359
4520	Adherens junction	4	0.0435

Table S3.5. GO pathway enrichment in upregulated group of OA versus normalcy

Pathway ID	Pathway description	Observed gene count	False discovery rate
GO.0006950	Response to stress	74	0.00183
GO.0016265	Death	35	0.00199
GO.0065008	Regulation of biological quality	66	0.00199
GO.0016042	Lipid catabolic process	15	0.00223
GO.0006629	Lipid metabolic process	33	0.00229
GO.0008219	Cell death	34	0.00229
GO.0012501	Programmed cell death	33	0.00254
GO.0002376	Immune system process	48	0.0039
GO.0006915	Apoptotic process	32	0.0039
GO.0032880	Regulation of protein localization	29	0.0039
GO.1901698	Response to nitrogen compound	28	0.0039
GO.0010243	Response to organonitrogen compound	26	0.00473
GO.0048584	Positive regulation of response to stimulus	46	0.00473
GO.0070887	Cellular response to chemical stimulus	52	0.00473
GO.0071496	Cellular response to external stimulus	15	0.00473
GO.0016477	Cell migration	25	0.00607
GO.0010033	Response to organic substance	54	0.00683
GO.0044710	Single-organism metabolic process	78	0.0124
GO.0031330	Negative regulation of cellular catabolic process	10	0.013
GO.0070201	Regulation of establishment of protein localization	25	0.0131
GO.0008610	Lipid biosynthetic process	19	0.0145
GO.0071310	Cellular response to organic substance	43	0.0149
GO.0002684	Positive regulation of immune system process	25	0.0151
GO.0006928	Movement of cell or subcellular component	34	0.0151
GO.0042981	Regulation of apoptotic process	35	0.0151
GO.0051674	Localization of cell	25	0.0151
GO.1901700	Response to oxygen-containing compound	34	0.0151
GO.0001775	Cell activation	21	0.0159
GO.0009895	Negative regulation of catabolic process	11	0.0159
GO.0044281	Small molecule metabolic process	47	0.0159
GO.0050878	Regulation of body fluid levels	22	0.0159
GO.0055114	Oxidation-reduction process	28	0.0159
GO.0051128	Regulation of cellular component organization	47	0.016
GO.0042127	Regulation of cell proliferation	36	0.017
GO.0010941	Regulation of cell death	36	0.0173
GO.0060341	Regulation of cellular localization	31	0.0198
GO.0044723	Single-organism carbohydrate metabolic process	20	0.0199

GO.0006955	Immune response	33	0.0208
GO.0044255	Cellular lipid metabolic process	24	0.0211
GO.0044711	Single-organism biosynthetic process	33	0.0211
GO.0097529	Myeloid leukocyte migration	7	0.0213
GO.0008104	Protein localization	39	0.0221
GO.0061024	Membrane organization	25	0.0228
GO.0042493	Response to drug	16	0.025
GO.0006952	Defense response	34	0.026
GO.0033036	Macromolecule localization	44	0.026
GO.0044242	Cellular lipid catabolic process	9	0.026
GO.0044282	Small molecule catabolic process	12	0.026
GO.0044712	Single-organism catabolic process	25	0.026
GO.0030155	Regulation of cell adhesion	19	0.0278
GO.0009967	Positive regulation of signal transduction	32	0.0303
GO.0051223	Regulation of protein transport	22	0.0303
GO.0097190	Apoptotic signaling pathway	14	0.0303
GO.0034613	Cellular protein localization	29	0.0312
GO.0040011	Locomotion	30	0.0312
GO.0045321	Leukocyte activation	15	0.0312
GO.0045937	Positive regulation of phosphate metabolic process	26	0.0317
GO.0046395	Carboxylic acid catabolic process	10	0.0317
GO.0048519	Negative regulation of biological process	75	0.0317
GO.1903426	Regulation of reactive oxygen species biosynthetic process	6	0.0317
GO.0001934	Positive regulation of protein phosphorylation	23	0.0318
GO.0009056	Catabolic process	39	0.0325
GO.0010647	Positive regulation of cell communication	36	0.0325
GO.0032879	Regulation of localization	47	0.0325
GO.0043113	Receptor clustering	5	0.0325
GO.0051239	Regulation of multicellular organismal process	48	0.0325
GO.0071812	Positive regulation of fever generation by positive regulation of prostaglandin secretion	2	0.0325
GO.0071848	Positive regulation of erk1 and erk2 cascade via tnfsf11-mediated signaling	2	0.0325
GO.1901575	Organic substance catabolic process	35	0.0325
GO.0048522	Positive regulation of cellular process	76	0.0326
GO.0048583	Regulation of response to stimulus	62	0.0326
GO.0009611	Response to wounding	22	0.0327
GO.0030162	Regulation of proteolysis	22	0.0327
GO.0009719	Response to endogenous stimulus	33	0.0336
GO.0051046	Regulation of secretion	20	0.0336
GO.0051050	Positive regulation of transport	24	0.0336

GO.0051641	Cellular localization	44	0.0336
GO.1903827	Regulation of cellular protein localization	17	0.0336
GO.0051701	Interaction with host	8	0.0359
GO.0002682	Regulation of immune system process	32	0.0373
GO.0050900	Leukocyte migration	11	0.0381
GO.1901137	Carbohydrate derivative biosynthetic process	19	0.0389
GO.0001932	Regulation of protein phosphorylation	29	0.0397
GO.0048518	Positive regulation of biological process	84	0.0399
GO.0032103	Positive regulation of response to external stimulus	14	0.0407
GO.0042327	Positive regulation of phosphorylation	23	0.0407
GO.1901135	Carbohydrate derivative metabolic process	26	0.041
GO.0030595	Leukocyte chemotaxis	7	0.042
GO.0001941	Postsynaptic membrane organization	4	0.0428
GO.0002696	Positive regulation of leukocyte activation	11	0.0428
GO.0005975	Carbohydrate metabolic process	22	0.0428
GO.0032386	Regulation of intracellular transport	18	0.0428
GO.0044248	Cellular catabolic process	33	0.0446
GO.0023056	Positive regulation of signaling	33	0.0455
GO.0045785	Positive regulation of cell adhesion	13	0.0455
GO.0001774	Microglial cell activation	3	0.0461
GO.0072330	Monocarboxylic acid biosynthetic process	9	0.0461
GO.1902531	Regulation of intracellular signal transduction	32	0.0461
GO.0010646	Regulation of cell communication	54	0.0491
GO.0051241	Negative regulation of multicellular organismal process	25	0.0491
GO.1903363	Negative regulation of cellular protein catabolic process	6	0.0491
GO.2001233	Regulation of apoptotic signaling pathway	14	0.0494

Table S3.6. KEGG pathway enrichment in upregulated group of OA versus normalcy

Pathway ID	Pathway description	Observed gene count	False discovery rate
1100	Metabolic pathways	35	0.00138
4142	Lysosome	9	0.00458

Table S3.7. GO pathway enrichment in downregulated group of OA versus normalcy

Pathway ID	Pathway description	Observed gene count	False discovery rate
GO.0009893	Positive regulation of metabolic process	59	9.33E-11
GO.0010604	Positive regulation of macromolecule metabolic process	50	2.60E-10
GO.0031325	Positive regulation of cellular metabolic process	51	4.52E-10
GO.0071310	Cellular response to organic substance	41	4.52E-10
GO.0010033	Response to organic substance	47	6.61E-10
GO.0048522	Positive regulation of cellular process	61	1.66E-08
GO.0051254	Positive regulation of rna metabolic process	34	2.19E-08
GO.0007275	Multicellular organismal development	58	3.62E-08
GO.0048518	Positive regulation of biological process	65	3.62E-08
GO.0070887	Cellular response to chemical stimulus	42	3.62E-08
GO.0007165	Signal transduction	62	4.06E-08
GO.0051173	Positive regulation of nitrogen compound metabolic process	36	5.36E-08
GO.0048731	System development	53	5.54E-08
GO.0010628	Positive regulation of gene expression	35	6.81E-08
GO.0006807	Nitrogen compound metabolic process	68	8.02E-08
GO.0010558	Negative regulation of macromolecule biosynthetic process	31	8.02E-08
GO.0019222	Regulation of metabolic process	73	9.71E-08
GO.0060255	Regulation of macromolecule metabolic process	66	9.71E-08
GO.0031328	Positive regulation of cellular biosynthetic process	35	1.07E-07
GO.0080090	Regulation of primary metabolic process	66	1.28E-07
GO.0042221	Response to chemical	53	1.81E-07
GO.0045934	Negative regulation of nucleobase-containing compound metabolic process	30	1.83E-07
GO.0010557	Positive regulation of macromolecule biosynthetic process	33	2.36E-07
GO.0044237	Cellular metabolic process	85	2.36E-07
GO.0045944	Positive regulation of transcription from rna polymerase ii promoter	26	2.36E-07
GO.0034641	Cellular nitrogen compound metabolic process	64	2.52E-07
GO.0016070	Rna metabolic process	51	2.58E-07
GO.0048523	Negative regulation of cellular process	54	2.58E-07
GO.1902680	Positive regulation of rna biosynthetic process	31	2.58E-07
GO.0048511	Rhythmic process	15	2.78E-07
GO.0010468	Regulation of gene expression	54	2.97E-07
GO.0051253	Negative regulation of rna metabolic process	28	3.02E-07
GO.0044260	Cellular macromolecule metabolic process	74	3.26E-07
GO.0031323	Regulation of cellular metabolic process	66	3.47E-07
GO.0044707	Single-multicellular organism process	65	3.85E-07

GO.0031327	Negative regulation of cellular biosynthetic process	30	4.36E-07
GO.0044700	Single organism signaling	61	5.17E-07
GO.0045893	Positive regulation of transcription		
GO.0090304	Nucleic acid metabolic process	54	5.23E-07
GO.0031326	Regulation of cellular biosynthetic process	53	5.62E-07
GO.0051252	Regulation of rna metabolic process	49	6.31E-07
GO.0051246	Regulation of protein metabolic process	40	6.34E-07
GO.0042325	Regulation of phosphorylation	28	7.77E-07
GO.0048519	Negative regulation of biological process	55	8.66E-07
GO.0010556	Regulation of macromolecule biosynthetic process	51	8.86E-07
GO.2000113	Negative regulation of cellular macromolecule biosynthetic process	28	8.86E-07
GO.0048856	Anatomical structure development	54	8.96E-07
GO.0006139	Nucleobase-containing compound metabolic process	57	9.20E-07
GO.0007154	Cell communication	61	9.38E-07
GO.0014074	Response to purine-containing compound	11	9.41E-07
GO.0042981	Regulation of apoptotic process	29	9.65E-07
GO.0010941	Regulation of cell death	30	9.98E-07
GO.0014070	Response to organic cyclic compound	22	9.98E-07
GO.1901360	Organic cyclic compound metabolic process	60	9.98E-07
GO.0032502	Developmental process	58	1.01E-06
GO.0051171	Regulation of nitrogen compound metabolic process	53	1.01E-06
GO.0051716	Cellular response to stimulus	67	1.01E-06
GO.0010467	Gene expression	53	1.05E-06
GO.2000112	Regulation of cellular macromolecule biosynthetic process	50	1.13E-06
GO.0032501	Multicellular organismal process	65	1.20E-06
GO.0008406	Gonad development	12	1.26E-06
GO.1903507	Negative regulation of nucleic acid-templated transcription	26	1.26E-06
GO.0001822	Kidney development	13	1.37E-06
GO.0009889	Regulation of biosynthetic process	52	1.57E-06
GO.0045137	Development of primary sexual characteristics	12	1.57E-06
GO.0048608	Reproductive structure development	16	1.57E-06
GO.0001944	Vasculature development	17	1.59E-06
GO.0010605	Negative regulation of macromolecule metabolic process	37	1.59E-06
GO.0044238	Primary metabolic process	83	1.59E-06
GO.0061458	Reproductive system development	16	1.72E-06
GO.0019219	Regulation of nucleobase-containing compound metabolic process	50	1.73E-06
GO.0044767	Single-organism developmental process	57	1.73E-06

GO.0051239	Regulation of multicellular organismal process	38	1.90E-06
GO.0001932	Regulation of protein phosphorylation	26	2.01E-06
GO.0007167	Enzyme linked receptor protein signaling pathway	23	2.01E-06
GO.0072001	Renal system development	13	2.07E-06
GO.0071495	Cellular response to endogenous stimulus	24	2.34E-06
GO.0007166	Cell surface receptor signaling pathway	35	2.38E-06
GO.0033993	Response to lipid	21	2.68E-06
GO.0046683	Response to organophosphorus	10	2.69E-06
GO.0042326	Negative regulation of phosphorylation	15	2.92E-06
GO.0051591	Response to camp	9	2.96E-06
GO.0000302	Response to reactive oxygen species	11	3.19E-06
GO.0045892	Negative regulation of transcription		
GO.0006355	Regulation of transcription		
GO.0001568	Blood vessel development	16	4.21E-06
GO.0006913	Nucleocytoplasmic transport	12	4.22E-06
GO.0010629	Negative regulation of gene expression	28	4.77E-06
GO.0008380	Rna splicing	14	4.86E-06
GO.0031324	Negative regulation of cellular metabolic process	36	4.86E-06
GO.2000026	Regulation of multicellular organismal development	29	5.26E-06
GO.0060548	Negative regulation of cell death	22	5.33E-06
GO.0006979	Response to oxidative stress	14	5.45E-06
GO.0001655	Urogenital system development	13	6.81E-06
GO.0009892	Negative regulation of metabolic process	38	6.91E-06
GO.0043066	Negative regulation of apoptotic process	21	6.95E-06
GO.0042127	Regulation of cell proliferation	28	7.86E-06
GO.0006357	Regulation of transcription from rna polymerase ii promoter	30	8.28E-06
GO.0009605	Response to external stimulus	32	8.55E-06
GO.0030154	Cell differentiation	43	8.56E-06
GO.0009653	Anatomical structure morphogenesis	34	8.97E-06
GO.0009628	Response to abiotic stimulus	24	9.31E-06
GO.0010243	Response to organonitrogen compound	20	1.00E-05
GO.0007548	Sex differentiation	12	1.01E-05
GO.0009719	Response to endogenous stimulus	27	1.11E-05
GO.0048513	Organ development	39	1.12E-05
GO.0019220	Regulation of phosphate metabolic process	28	1.20E-05
GO.0043170	Macromolecule metabolic process	73	1.25E-05
GO.0045936	Negative regulation of phosphate metabolic process	16	1.31E-05
GO.1901700	Response to oxygen-containing compound	26	1.31E-05
GO.0016071	Mrna metabolic process	17	1.44E-05
GO.0034599	Cellular response to oxidative stress	10	1.44E-05
GO.0048514	Blood vessel morphogenesis	14	1.57E-05
GO.0043549	Regulation of kinase activity	19	1.58E-05

GO.0000398	Mrna splicing		
GO.0051240	Positive regulation of multicellular organismal process	26	1.65E-05
GO.0032268	Regulation of cellular protein metabolic process	35	1.68E-05
GO.0009059	Macromolecule biosynthetic process	48	1.75E-05
GO.0071704	Organic substance metabolic process	81	1.98E-05
GO.0032496	Response to lipopolysaccharide	12	2.04E-05
GO.0000122	Negative regulation of transcription from rna polymerase ii promoter	19	2.57E-05
GO.0045859	Regulation of protein kinase activity	18	2.62E-05
GO.0051704	Multi-organism process	34	2.78E-05
GO.0031399	Regulation of protein modification process	28	2.85E-05
GO.0006397	Mrna processing	14	2.89E-05
GO.0001933	Negative regulation of protein phosphorylation	13	2.91E-05
GO.0032870	Cellular response to hormone stimulus	16	3.22E-05
GO.0044271	Cellular nitrogen compound biosynthetic process	45	3.56E-05
GO.0051726	Regulation of cell cycle	21	3.56E-05
GO.1901698	Response to nitrogen compound	20	3.56E-05
GO.0007623	Circadian rhythm	9	3.70E-05
GO.1901576	Organic substance biosynthetic process	54	3.70E-05
GO.0006351	Transcription		
GO.0051338	Regulation of transferase activity	20	5.04E-05
GO.0009612	Response to mechanical stimulus	10	5.51E-05
GO.0009888	Tissue development	27	5.79E-05
GO.0023057	Negative regulation of signaling	23	5.91E-05
GO.0034645	Cellular macromolecule biosynthetic process	46	5.91E-05
GO.0010648	Negative regulation of cell communication	23	6.74E-05
GO.0050896	Response to stimulus	68	6.86E-05
GO.0009991	Response to extracellular stimulus	14	8.86E-05
GO.0030947	Regulation of vascular endothelial growth factor receptor signaling pathway	5	9.14E-05
GO.0048660	Regulation of smooth muscle cell proliferation	7	9.15E-05
GO.0008152	Metabolic process	84	9.16E-05
GO.0051094	Positive regulation of developmental process	22	9.16E-05
GO.0030334	Regulation of cell migration	16	0.000101
GO.0003006	Developmental process involved in reproduction	16	0.000103
GO.0033673	Negative regulation of kinase activity	10	0.00012
GO.0044249	Cellular biosynthetic process	52	0.000125
GO.0033554	Cellular response to stress	27	0.000135
GO.0009968	Negative regulation of signal transduction	21	0.000155
GO.0034097	Response to cytokine	16	0.000166
GO.0043065	Positive regulation of apoptotic process	15	0.000167
GO.0072358	Cardiovascular system development	18	0.000167
GO.0072359	Circulatory system development	18	0.000167
GO.0032922	Circadian regulation of gene expression	6	0.000169
GO.0040007	Growth	13	0.000169

GO.0043618	Regulation of transcription from rna polymerase ii promoter in response to stress	6	0.000169
GO.0050793	Regulation of developmental process	31	0.000171
GO.0008284	Positive regulation of cell proliferation	18	0.000178
GO.0051270	Regulation of cellular component movement	17	0.000186
GO.0031667	Response to nutrient levels	13	0.000204
GO.0008585	Female gonad development	7	0.000207
GO.0006950	Response to stress	42	0.000209
GO.0010035	Response to inorganic substance	13	0.000218
GO.0022414	Reproductive process	22	0.00022
GO.0030949	Positive regulation of vascular endothelial growth factor receptor signaling pathway	4	0.000236
GO.0070848	Response to growth factor	16	0.000261
GO.0001525	Angiogenesis	11	0.000268
GO.0042542	Response to hydrogen peroxide	7	0.000282
GO.0046545	Development of primary female sexual characteristics	7	0.000282
GO.0001701	In utero embryonic development	12	0.000297
GO.0048585	Negative regulation of response to stimulus	23	0.00033
GO.1903311	Regulation of mrna metabolic process	7	0.000362
GO.2000172	Regulation of branching morphogenesis of a nerve	3	0.000395
GO.0034654	Nucleobase-containing compound biosynthetic process	39	0.000414
GO.0097305	Response to alcohol	11	0.00043
GO.0006469	Negative regulation of protein kinase activity	9	0.000437
GO.1901652	Response to peptide	13	0.000463
GO.0009314	Response to radiation	13	0.000471
GO.0007169	Transmembrane receptor protein tyrosine kinase signaling pathway	16	0.000487
GO.1902532	Negative regulation of intracellular signal transduction	12	0.000506
GO.0046660	Female sex differentiation	7	0.000604
GO.0051168	Nuclear export	7	0.000604
GO.1901362	Organic cyclic compound biosynthetic process	40	0.000636
GO.0006396	Rna processing	16	0.000641
GO.0036003	Positive regulation of transcription from rna polymerase ii promoter in response to stress	4	0.00067
GO.0048545	Response to steroid hormone	12	0.000671
GO.0071363	Cellular response to growth factor stimulus	15	0.000702
GO.1902531	Regulation of intracellular signal transduction	23	0.000709
GO.0040011	Locomotion	21	0.000747
GO.0042593	Glucose homeostasis	8	0.000847
GO.0002521	Leukocyte differentiation	10	0.000939
GO.0006405	Rna export from nucleus	6	0.000991
GO.0043525	Positive regulation of neuron apoptotic process	5	0.00104

GO.0016477	Cell migration	16	0.0011
GO.0051028	Mrna transport	7	0.00123
GO.0002520	Immune system development	14	0.00125
GO.0007399	Nervous system development	28	0.00126
GO.0043484	Regulation of rna splicing	6	0.00127
GO.0090287	Regulation of cellular response to growth factor stimulus	9	0.00127
GO.0044702	Single organism reproductive process	19	0.00152
GO.0050794	Regulation of cellular process	77	0.00154
GO.0009725	Response to hormone	17	0.00155
GO.0043408	Regulation of mapk cascade	14	0.00155
GO.0065008	Regulation of biological quality	36	0.00181
GO.0050684	Regulation of mrna processing	6	0.00184
GO.0006366	Transcription from rna polymerase ii promoter	16	0.00186
GO.0001541	Ovarian follicle development	5	0.00191
GO.0060429	Epithelium development	18	0.00195
GO.0009966	Regulation of signal transduction	31	0.00199
GO.1903706	Regulation of hemopoiesis	10	0.00211
GO.0051247	Positive regulation of protein metabolic process	22	0.00217
GO.1901701	Cellular response to oxygen-containing compound	16	0.00217
GO.0001775	Cell activation	14	0.0022
GO.0035556	Intracellular signal transduction	26	0.00221
GO.0045321	Leukocyte activation	11	0.00221
GO.0048646	Anatomical structure formation involved in morphogenesis	18	0.00221
GO.0022008	Neurogenesis	22	0.00236
GO.0061394	Regulation of transcription from rna polymerase ii promoter in response to arsenic-containing substance	2	0.00243
GO.0070372	Regulation of erk1 and erk2 cascade	8	0.00243
GO.2000173	Negative regulation of branching morphogenesis of a nerve	2	0.00243
GO.0007159	Leukocyte cell-cell adhesion	9	0.00247
GO.0048583	Regulation of response to stimulus	38	0.00277
GO.0051674	Localization of cell	16	0.00277
GO.0042698	Ovulation cycle	6	0.00288
GO.0051170	Nuclear import	6	0.00288
GO.0006928	Movement of cell or subcellular component	21	0.00294
GO.0002376	Immune system process	27	0.00295
GO.0045785	Positive regulation of cell adhesion	10	0.00295
GO.0023051	Regulation of signaling	33	0.00296
GO.0048534	Hematopoietic or lymphoid organ development	13	0.00298
GO.0048699	Generation of neurons	21	0.00308
GO.0032868	Response to insulin	9	0.00312
GO.0036499	Perk-mediated unfolded protein response	3	0.00312

GO.1990440	Positive regulation of transcription from rna polymerase ii promoter in response to endoplasmic reticulum stress	3	0.00312
GO.0048008	Platelet-derived growth factor receptor signaling pathway	4	0.00341
GO.0009266	Response to temperature stimulus	8	0.00344
GO.0001570	Vasculogenesis	5	0.00373
GO.0002053	Positive regulation of mesenchymal cell proliferation	4	0.00379
GO.0009611	Response to wounding	15	0.00387
GO.0006403	Rna localization	7	0.00416
GO.0008283	Cell proliferation	14	0.00418
GO.0045429	Positive regulation of nitric oxide biosynthetic process	4	0.00418
GO.0009790	Embryo development	17	0.00425
GO.0044706	Multi-multicellular organism process	8	0.00448
GO.0051592	Response to calcium ion	6	0.00448
GO.0030097	Hemopoiesis	12	0.00485
GO.0006406	Mrna export from nucleus	5	0.0051
GO.0071427	Mrna-containing ribonucleoprotein complex export from nucleus	5	0.0051
GO.0050789	Regulation of biological process	77	0.00519
GO.0071496	Cellular response to external stimulus	9	0.00532
GO.0051090	Regulation of sequence-specific dna binding transcription factor activity	10	0.00533
GO.0070486	Leukocyte aggregation	8	0.00547
GO.0065009	Regulation of molecular function	32	0.0056
GO.0008584	Male gonad development	6	0.00599
GO.0031100	Organ regeneration	5	0.00599
GO.0044403	Symbiosis		
GO.0046546	Development of primary male sexual characteristics	6	0.00599
GO.0050790	Regulation of catalytic activity	28	0.00599
GO.1903034	Regulation of response to wounding	10	0.00599
GO.1902105	Regulation of leukocyte differentiation	8	0.006
GO.0002690	Positive regulation of leukocyte chemotaxis	5	0.00673
GO.0009617	Response to bacterium	11	0.00687
GO.0010646	Regulation of cell communication	33	0.00687
GO.0016482	Cytoplasmic transport	14	0.00687
GO.0043009	Chordate embryonic development	13	0.00687
GO.1900744	Regulation of p38mapk cascade	3	0.00687
GO.1901214	Regulation of neuron death	8	0.00687
GO.0097529	Myeloid leukocyte migration	5	0.007
GO.0006935	Chemotaxis	13	0.00725
GO.2000379	Positive regulation of reactive oxygen species metabolic process	5	0.00736

GO.0009749	Response to glucose	6	0.00754
GO.0044319	Wound healing		
GO.0090505	Epiboly involved in wound healing	3	0.00806
GO.0016265	Death	18	0.00862
GO.0044763	Single-organism cellular process	80	0.00882
GO.0023014	Signal transduction by protein phosphorylation	8	0.00901
GO.0071277	Cellular response to calcium ion	4	0.00901
GO.0001763	Morphogenesis of a branching structure	7	0.00933
GO.0002682	Regulation of immune system process	20	0.00933
GO.0007568	Aging	8	0.00933
GO.0030728	Ovulation	3	0.00933
GO.0050727	Regulation of inflammatory response	8	0.00933
GO.0071166	Ribonucleoprotein complex localization	5	0.00933
GO.0008285	Negative regulation of cell proliferation	13	0.00939
GO.0045637	Regulation of myeloid cell differentiation	7	0.00939
GO.0046649	Lymphocyte activation	9	0.0094
GO.0017038	Protein import	6	0.00956
GO.0045595	Regulation of cell differentiation	21	0.00958
GO.0043523	Regulation of neuron apoptotic process	7	0.00992
GO.0048589	Developmental growth	9	0.00998
GO.0051054	Positive regulation of dna metabolic process	6	0.0102
GO.1901654	Response to ketone	6	0.0102
GO.0030155	Regulation of cell adhesion	12	0.0105
GO.0031175	Neuron projection development	13	0.0105
GO.0071241	Cellular response to inorganic substance	6	0.0105
GO.1901653	Cellular response to peptide	9	0.0105
GO.0051093	Negative regulation of developmental process	15	0.0106
GO.0071417	Cellular response to organonitrogen compound	11	0.0107
GO.0048732	Gland development	10	0.0108
GO.0070301	Cellular response to hydrogen peroxide	4	0.0108
GO.0051179	Localization	43	0.0109
GO.0043409	Negative regulation of mapk cascade	6	0.0112
GO.0060711	Labyrinthine layer development	4	0.0115
GO.0032269	Negative regulation of cellular protein metabolic process	16	0.0116
GO.0048468	Cell development	22	0.0117
GO.0000288	Nuclear-transcribed mrna catabolic process		
GO.0048661	Positive regulation of smooth muscle cell proliferation	4	0.0123
GO.0051241	Negative regulation of multicellular organismal process	16	0.0125
GO.0034614	Cellular response to reactive oxygen species	5	0.0126
GO.2000377	Regulation of reactive oxygen species metabolic process	6	0.0128
GO.0001892	Embryonic placenta development	5	0.0129
GO.0009887	Organ morphogenesis	15	0.0129

GO.0009987	Cellular process	92	0.0129
GO.0010332	Response to gamma radiation	4	0.0129
GO.0030335	Positive regulation of cell migration	9	0.0129
GO.0031099	Regeneration	6	0.0129
GO.0032101	Regulation of response to external stimulus	15	0.0129
GO.0042493	Response to drug	10	0.0129
GO.0046661	Male sex differentiation	6	0.0129
GO.0060713	Labyrinthine layer morphogenesis	3	0.0132
GO.0032270	Positive regulation of cellular protein metabolic process	19	0.0135
GO.0042060	Wound healing	13	0.0137
GO.0043434	Response to peptide hormone	10	0.0137
GO.0043488	Regulation of mrna stability	4	0.0137
GO.0051384	Response to glucocorticoid	6	0.0137
GO.0061564	Axon development	11	0.0137
GO.0071333	Cellular response to glucose stimulus	4	0.0137
GO.0032675	Regulation of interleukin-6 production	5	0.0139
GO.0080134	Regulation of response to stress	20	0.0142
GO.0060688	Regulation of morphogenesis of a branching structure	4	0.0145
GO.0001890	Placenta development	6	0.0146
GO.0001817	Regulation of cytokine production	11	0.0153
GO.0016337	Single organismal cell-cell adhesion	10	0.0153
GO.0030213	Hyaluronan biosynthetic process	2	0.0153
GO.0031427	Response to methotrexate	2	0.0153
GO.0050679	Positive regulation of epithelial cell proliferation	6	0.0153
GO.0071900	Regulation of protein serine/threonine kinase activity	10	0.0153
GO.0006606	Protein import into nucleus	5	0.0155
GO.0044744	Protein targeting to nucleus	5	0.0155
GO.1901342	Regulation of vasculature development	7	0.0158
GO.0030595	Leukocyte chemotaxis	5	0.016
GO.0032869	Cellular response to insulin stimulus	7	0.016
GO.0045669	Positive regulation of osteoblast differentiation	4	0.016
GO.0048024	Regulation of mrna splicing		
GO.0048666	Neuron development	14	0.0162
GO.0002683	Negative regulation of immune system process	9	0.0166
GO.0009408	Response to heat	6	0.0166
GO.0051052	Regulation of dna metabolic process	8	0.0166
GO.0071345	Cellular response to cytokine stimulus	11	0.0166
GO.0006986	Response to unfolded protein	6	0.0169
GO.0002009	Morphogenesis of an epithelium	10	0.017
GO.0048639	Positive regulation of developmental growth	6	0.0175
GO.0048667	Cell morphogenesis involved in neuron differentiation	11	0.0178
GO.0016032	Viral process	13	0.0181

GO.0008219	Cell death	17	0.0182
GO.0031668	Cellular response to extracellular stimulus	7	0.0182
GO.0042110	T cell activation	7	0.0182
GO.0010038	Response to metal ion	8	0.0184
GO.0007178	Transmembrane receptor protein serine/threonine kinase signaling pathway	7	0.0186
GO.0002832	Negative regulation of response to biotic stimulus	3	0.0194
GO.0006376	Mrna splice site selection	3	0.0194
GO.0060669	Embryonic placenta morphogenesis	3	0.0194
GO.1903708	Positive regulation of hemopoiesis	6	0.0194
GO.0051248	Negative regulation of protein metabolic process	16	0.0199
GO.0048812	Neuron projection morphogenesis	11	0.0207
GO.0060710	Chorio-allantoic fusion	2	0.0207
GO.0060754	Positive regulation of mast cell chemotaxis	2	0.0207
GO.1901724	Positive regulation of cell proliferation involved in kidney development	2	0.0207
GO.2000323	Negative regulation of glucocorticoid receptor signaling pathway	2	0.0207
GO.0060326	Cell chemotaxis	6	0.021
GO.0033280	Response to vitamin d	3	0.0214
GO.0042327	Positive regulation of phosphorylation	14	0.0219
GO.0007346	Regulation of mitotic cell cycle	10	0.0221
GO.0034248	Regulation of cellular amide metabolic process	8	0.0222
GO.0005979	Regulation of glycogen biosynthetic process	3	0.0234
GO.0014072	Response to isoquinoline alkaloid	3	0.0234
GO.0030325	Adrenal gland development	3	0.0234
GO.0045740	Positive regulation of dna replication	4	0.0243
GO.0048878	Chemical homeostasis	14	0.0245
GO.0009896	Positive regulation of catabolic process	10	0.0256
GO.0045596	Negative regulation of cell differentiation	12	0.0271
GO.0002761	Regulation of myeloid leukocyte differentiation	5	0.0275
GO.1903203	Regulation of oxidative stress-induced neuron death	2	0.0275
GO.0071375	Cellular response to peptide hormone stimulus	8	0.028
GO.0030212	Hyaluronan metabolic process	3	0.0283
GO.0048662	Negative regulation of smooth muscle cell proliferation	3	0.0283
GO.0043407	Negative regulation of map kinase activity	4	0.0284
GO.0006915	Apoptotic process	16	0.0288
GO.0071901	Negative regulation of protein serine/threonine kinase activity	5	0.0305
GO.0007565	Female pregnancy	6	0.0308
GO.0061138	Morphogenesis of a branching epithelium	6	0.0308
GO.0010574	Regulation of vascular endothelial growth factor production	3	0.0309

GO.0007409	Axonogenesis	10	0.0311
GO.0048729	Tissue morphogenesis	11	0.0311
GO.0051707	Response to other organism	12	0.0311
GO.0071248	Cellular response to metal ion	5	0.0311
GO.0044092	Negative regulation of molecular function	16	0.0318
GO.0051960	Regulation of nervous system development	12	0.0319
GO.0001889	Liver development	5	0.032
GO.0006275	Regulation of dna replication	5	0.0327
GO.0046627	Negative regulation of insulin receptor signaling pathway	3	0.0327
GO.0050767	Regulation of neurogenesis	11	0.0327
GO.0060674	Placenta blood vessel development	3	0.0327
GO.0070663	Regulation of leukocyte proliferation	6	0.0327
GO.1904018	Positive regulation of vasculature development	5	0.0327
GO.0030030	Cell projection organization	15	0.0329
GO.0002666	Positive regulation of t cell tolerance induction	2	0.0339
GO.0021796	Cerebral cortex regionalization	2	0.0339
GO.0030324	Lung development	6	0.0339
GO.0031620	Regulation of fever generation	2	0.0339
GO.0031665	Negative regulation of lipopolysaccharide-mediated signaling pathway	2	0.0339
GO.0035767	Endothelial cell chemotaxis	2	0.0339
GO.0071559	Response to transforming growth factor beta	6	0.0339
GO.0061008	Hepaticobiliary system development	5	0.0346
GO.0065007	Biological regulation	75	0.0354
GO.0030217	T cell differentiation	5	0.0358
GO.2000177	Regulation of neural precursor cell proliferation	4	0.0366
GO.0030323	Respiratory tube development	6	0.0375
GO.0051249	Regulation of lymphocyte activation	8	0.038
GO.0030879	Mammary gland development	5	0.0381
GO.0051817	Modification of morphology or physiology of other organism involved in symbiotic interaction	4	0.0382
GO.0001934	Positive regulation of protein phosphorylation	13	0.0401
GO.0006468	Protein phosphorylation	13	0.0401
GO.0031669	Cellular response to nutrient levels	6	0.0401
GO.0042594	Response to starvation	6	0.0401
GO.0071222	Cellular response to lipopolysaccharide	5	0.0405
GO.0045597	Positive regulation of cell differentiation	13	0.0412
GO.0044267	Cellular protein metabolic process	34	0.0415
GO.1902107	Positive regulation of leukocyte differentiation	5	0.0415
GO.0016973	Poly(a)+ mrna export from nucleus	2	0.0416
GO.0051234	Establishment of localization	35	0.0416
GO.0097190	Apoptotic signaling pathway	8	0.0422
GO.0070555	Response to interleukin-1	4	0.0431
GO.0045765	Regulation of angiogenesis	6	0.046
GO.0045860	Positive regulation of protein kinase activity	9	0.046

GO.0001666	Response to hypoxia	7	0.0463
GO.0002684	Positive regulation of immune system process	13	0.0469
GO.0010212	Response to ionizing radiation	5	0.0483
GO.0080135	Regulation of cellular response to stress	12	0.0485
GO.0050678	Regulation of epithelial cell proliferation	7	0.049
GO.0030098	Lymphocyte differentiation	6	0.0491
GO.0010761	Fibroblast migration	2	0.0498
GO.0070935	3 -utr-mediated mrna stabilization	2	0.0498
GO.1900745	Positive regulation of p38mapk cascade	2	0.0498

Table S3.8. KEGG pathway enrichment in downregulated group of OA versus normalcy

Pathway ID	Pathway description	Observed gene count	False discovery rate
4668	TNF signaling pathway	11	3.76E-08
4010	MAPK signaling pathway	14	2.17E-07
4380	Osteoclast differentiation	9	1.37E-05
5168	Herpes simplex infection	10	1.98E-05
5166	HTLV-I infection	11	5.71E-05
3040	Spliceosome	8	9.03E-05
4068	FoxO signaling pathway	7	0.000601
4151	PI3K-Akt signaling pathway	11	0.000601
5134	Legionellosis	5	0.000885
4621	NOD-like receptor signaling pathway	5	0.00104
5206	MicroRNAs in cancer	7	0.00133
4932	Non-alcoholic fatty liver disease (NAFLD)	7	0.00145
5164	Influenza A	7	0.00286
5162	Measles	6	0.0048
4064	NF-kappa B signaling pathway	5	0.00563
5200	Pathways in cancer	9	0.00563
4060	Cytokine-cytokine receptor interaction	8	0.00629
4915	Estrogen signaling pathway	5	0.00647
5142	Chagas disease (American trypanosomiasis)	5	0.00709
4620	Toll-like receptor signaling pathway	5	0.00825
4630	Jak-STAT signaling pathway	6	0.00825
4710	Circadian rhythm	3	0.0123
5133	Pertussis	4	0.0142
5220	Chronic myeloid leukemia	4	0.0142
4152	AMPK signaling pathway	5	0.0143
5020	Prion diseases	3	0.019
5169	Epstein-Barr virus infection	6	0.019
4910	Insulin signaling pathway	5	0.0201
5132	Salmonella infection	4	0.0213
4640	Hematopoietic cell lineage	4	0.023
5161	Hepatitis B	5	0.023
5323	Rheumatoid arthritis	4	0.023
4930	Type II diabetes mellitus	3	0.0295
5144	Malaria	3	0.0305
4660	T cell receptor signaling pathway	4	0.0345
5030	Cocaine addiction	3	0.0345
4066	HIF-1 signaling pathway	4	0.0396
5210	Colorectal cancer	3	0.0499

Table S4. Biological functions of 16 differential biomarker candidates.

Entrez ID	Approved symbol	Approved name	Chromosomal location	Combined effects size[1]	Biological function
7111	TMOD1	Tropomodulin 1	9q22.33	-2.16	TMOD1 encodes tropomodulin 1, a member of the tropomodulin family. Tropomodulin 1 tightly caps tropomyosin-actin pointed ends, blocking actin monomer association and elongation of the pointed ends [1]. The overexpression of TMOD1 mediated by NF-kappaB was associated with enhanced growth of triple-negative breast cancer [2]. The role of TMOD1 in inflammatory diseases, especially in arthritis, has not been studied thoroughly.
10248	POP7	POP7 homolog, ribonuclease P/MRP subunit	7q22.1	-0.87	The role of POP7 in inflammatory diseases, especially in arthritis, has not been studied thoroughly.
6442	SGCA	Sarcoglycan alpha	17q21.33	-2.74	SGCA encodes the alpha-sarcoglycan subunit of the dystrophin-glycoprotein complex whose absence resulted in the loss of linkage between the actin cytoskeleton and the extracellular matrix, causing the instability of sarcolemma under physical stress [3]. Mutations in this gene were associated with increased prevalence of limb-girdle muscular dystrophy [4,5]. The role of SGCA in inflammatory diseases, especially in arthritis, has not been studied thoroughly.
3824	KLRD1	Killer cell lectin like receptor D1	12p13	1.35	KLRD1, also known as CD94, is a peptide-selective natural killer (NK) cell receptor. The higher level of CD94 was reported in RA patients response to TNF inhibitors therapy than in active RA patients [6]. CD94 also played a crucial role in the regulation of cytokine secretion in RA [7].
240	ALOX5	Arachidonate 5-lipoxygenase	10q11.21	1.36	ALOX5 encodes 5-lipoxygenase, an enzyme necessary for the oxidation of arachidonic acid to leukotriene A4. Leukotrienes, in turn, are major mediators of a variety of inflammatory and allergic conditions, including

					arthritis [8].
57403	RAB22A	RAB22A, member RAS oncogene family	20q13.32	-1.20	RAB22A encodes a member of the RAB family of small GTPases being implicated in the early endocytic pathway [9]. RAB22A may affect the transport from endosomes to the Golgi apparatus [10]. The overexpression of RAB22A promoted some tumor growth and invasion, such as melanoma and lung cancer [11,12]. The role of RAB22A in inflammatory diseases, especially in arthritis, has not been studied thoroughly.
288	ANK3	Ankyrin 3	10q21.2	-1.92	ANK3 encodes the ankyrin G protein that links the integral membrane proteins to the spectrin-actin cytoskeleton. ANK3 was originally localised at axon initial segments and nodes of Ranvier of neurons and was involved in the initiation and propagation of action potentials by grouping sodium channels [13]. The role of ANK3 in inflammatory diseases, especially in arthritis, has not been studied thoroughly.
5774	PTPN3	Protein tyrosine phosphatase, non-receptor type 3	9q31	-1.31	PTPN3 encodes a member of the protein tyrosine phosphatase family. Mutations of PTPN3 were known to be associated with a number of biological events, including cell proliferation, migration and cholangiocarcinoma recurrence [14]. The role of PTPN3 in inflammatory diseases, especially in arthritis, has not been studied thoroughly.
3003	GZMK	Granzyme K	5q11.2	2.92	GZMK encodes granzyme K in the cytoplasmic granules of cytolytic T lymphocytes and natural killer cells, mediating target cell death and controlling inflammation. Extracellular granzyme K promoted proinflammatory responses in endothelial cells [15] and induced cytokine secretion by binding to Gram-negative bacteria and their cell-wall lipopolysaccharide [16].
1191	CLU	Clusterin	8p21.1	-2.17	CLU encodes clusterin. It has been suggested to be implicated in several biological processes such as

					tumor growth and cell death [17]. The role of CLU in inflammatory diseases, especially in arthritis, has not been studied thoroughly.
3002	GZMB	Granzyme B	14q12	2.61	GZMB encodes granzyme B which was demonstrated to be involved in the erosive progression of RA and may become prognostic biomarker of early RA [18]. High level of GZMB was found in the plasma and synovial fluid of RA patients [19].
23194	FBXL7	F-box and leucine rich repeat protein 7	5p15.1	-0.93	FBXL7 encodes a member of the F-box protein family. FBXL7 was reported to regulate mitotic cell cycle progression by poly-ubiquitinating and degrading Aurora A, a key mediator of mitotic spindle formation and chromosome segregation [20]. The role of FBXL7 in inflammatory diseases, especially in arthritis, has not been studied thoroughly.
7293	TNFRSF4	TNF receptor superfamily member 4	1p36.33	1.26	TNFRSF4 encodes a member of the TNF receptor super family. TNFRSF4 and its ligands were the co-stimulatory molecules for T lymphocytes, controlled cytokine production from T cells, and regulated cytokine receptor signaling. They have become therapeutic targets for a number of inflammatory and autoimmune diseases [21].
9235	IL32	Interleukin 32	16p13.3	2.73	IL32 encodes a member of the cytokine family, interleukin 32 whose overexpression was induced by pathogens and proinflammatory cytokines. IL32 was also involved in the pathogenesis of inflammatory diseases and cancer [22].
439921	MXRA7	Matrix remodeling associated 7	17q25.1	-2.19	The role of MXRA7 in inflammatory diseases, especially in arthritis, has not been studied thoroughly.
925	CD8A	CD8a molecule	2p11.2	2.83	CD8A encodes CD8 alpha chain of the CD8 glycoprotein found on the surface of most cytotoxic T lymphocytes that acts for antigens recognition and T cells activation. The CD8 T cells function in both adaptive and innate immunity [23].

[1]: Adopted from the the section of functional meta-analysis, which was independent to the biomarker selection.

Reference

1. Yamashiro, S.; Gokhin, D.S.; Kimura, S.; Nowak, R.B.; Fowler, V.M. Tropomodulins: pointed-end capping proteins that regulate actin filament architecture in diverse cell types. *Cytoskeleton (Hoboken, N.J.)* **2012**, *69*, 337-370, doi:10.1002/cm.21031.
2. Ito-Kureha, T.; Koshikawa, N.; Yamamoto, M.; Semba, K.; Yamaguchi, N.; Yamamoto, T.; Seiki, M.; Inoue, J. Tropomodulin 1 expression driven by NF-kappaB enhances breast cancer growth. *Cancer research* **2015**, *75*, 62-72, doi:10.1158/0008-5472.can-13-3455.
3. Matsumura, K.; Campbell, K.P. Dystrophin-glycoprotein complex: its role in the molecular pathogenesis of muscular dystrophies. *Muscle & nerve* **1994**, *17*, 2-15, doi:10.1002/mus.880170103.
4. Liang, W.C.; Chou, P.C.; Hung, C.C.; Su, Y.N.; Kan, T.M.; Chen, W.Z.; Hayashi, Y.K.; Nishino, I.; Jong, Y.J. Probable high prevalence of limb-girdle muscular dystrophy type 2D in Taiwan. *Journal of the neurological sciences* **2016**, *362*, 304-308, doi:10.1016/j.jns.2016.02.002.
5. Reddy, H.M.; Hamed, S.A.; Lek, M.; Mitsuhashi, S.; Estrella, E.; Jones, M.D.; Mahoney, L.J.; Duncan, A.R.; Cho, K.A.; Macarthur, D.G., et al. Homozygous nonsense mutation in SGCA is a common cause of limb-girdle muscular dystrophy in Assiut, Egypt. *Muscle & nerve* **2016**, *54*, 690-695, doi:10.1002/mus.25094.
6. Walsh, C.E.; Ryan, E.J.; O'Farrelly, C.; Golden-Mason, L.; FitzGerald, O.; Veale, D.J.; Bresnihan, B.; Fearon, U. Differential expression of NK receptors CD94 and NKG2A by T cells in rheumatoid arthritis patients in remission compared to active disease. *PloS one* **2011**, *6*, e27182, doi:10.1371/journal.pone.0027182.
7. de Matos, C.T.; Berg, L.; Michaelsson, J.; Fellander-Tsai, L.; Karre, K.; Soderstrom, K. Activating and inhibitory receptors on synovial fluid natural killer cells of arthritis patients: role of CD94/NKG2A in control of cytokine secretion. *Immunology* **2007**, *122*, 291-301, doi:10.1111/j.1365-2567.2007.02638.x.
8. Assimes, T.L.; Knowles, J.W.; Priest, J.R.; Basu, A.; Volcik, K.A.; Southwick, A.; Tabor, H.K.; Hartiala, J.; Allayee, H.; Grove, M.L., et al. Common polymorphisms of ALOX5 and ALOX5AP and risk of coronary artery disease. *Human genetics* **2008**, *123*, 399-408, doi:10.1007/s00439-008-0489-5.
9. Mesa, R.; Salomon, C.; Roggero, M.; Stahl, P.D.; Mayorga, L.S. Rab22a affects the morphology and function of the endocytic pathway. *Journal of cell science* **2001**, *114*, 4041-4049.
10. Mesa, R.; Magadan, J.; Barbieri, A.; Lopez, C.; Stahl, P.D.; Mayorga, L.S. Overexpression of Rab22a hampers the transport between endosomes and the Golgi apparatus. *Experimental cell research* **2005**, *304*, 339-353, doi:10.1016/j.yexcr.2004.11.017.
11. Su, F.; Chen, Y.; Zhu, S.; Li, F.; Zhao, S.; Wu, L.; Chen, X.; Su, J. RAB22A overexpression promotes the tumor growth of melanoma. *Oncotarget* **2016**, *7*, 71744-71753, doi:10.18632/oncotarget.12329.
12. Zhou, Y.; Wu, B.; Li, J.H.; Nan, G.; Jiang, J.L.; Chen, Z.N. Rab22a enhances CD147 recycling and is required for lung cancer cell migration and invasion. *Experimental cell research* **2017**, *357*, 9-16, doi:10.1016/j.yexcr.2017.04.020.
13. Zhou, D.; Lambert, S.; Malen, P.L.; Carpenter, S.; Boland, L.M.; Bennett, V. AnkyrinG is required for clustering of voltage-gated Na channels at axon initial segments and for normal action potential firing. *The Journal of cell biology* **1998**, *143*, 1295-1304.
14. Gao, Q.; Zhao, Y.J.; Wang, X.Y.; Guo, W.J.; Gao, S.; Wei, L.; Shi, J.Y.; Shi, G.M.; Wang, Z.C.; Zhang, Y.N., et al. Activating mutations in PTPN3 promote cholangiocarcinoma cell proliferation and migration and are associated with tumor recurrence in patients. *Gastroenterology* **2014**, *146*, 1397-1407, doi:10.1053/j.gastro.2014.01.062.

15. Sharma, M.; Merkulova, Y.; Raithatha, S.; Parkinson, L.G.; Shen, Y.; Cooper, D.; Granville, D.J. Extracellular granzyme K mediates endothelial activation through the cleavage of protease-activated receptor-1. *The FEBS journal* **2016**, *283*, 1734-1747, doi:10.1111/febs.13699.
16. Wensink, A.C.; Kemp, V.; Fermie, J.; Garcia Laorden, M.I.; van der Poll, T.; Hack, C.E.; Bovenschen, N. Granzyme K synergistically potentiates LPS-induced cytokine responses in human monocytes. *Proceedings of the National Academy of Sciences of the United States of America* **2014**, *111*, 5974-5979, doi:10.1073/pnas.1317347111.
17. Trougakos, I.P.; Lourda, M.; Antonelou, M.H.; Kletsas, D.; Gorgoulis, V.G.; Papassideri, I.S.; Zou, Y.; Margaritis, L.H.; Boothman, D.A.; Gonos, E.S. Intracellular clusterin inhibits mitochondrial apoptosis by suppressing p53-activating stress signals and stabilizing the cytosolic Ku70-Bax protein complex. *Clinical cancer research : an official journal of the American Association for Cancer Research* **2009**, *15*, 48-59, doi:10.1158/1078-0432.ccr-08-1805.
18. Goldbach-Mansky, R.; Suson, S.; Wesley, R.; Hack, C.E.; El-Gabalawy, H.S.; Tak, P.P. Raised granzyme B levels are associated with erosions in patients with early rheumatoid factor positive rheumatoid arthritis. *Annals of the rheumatic diseases* **2005**, *64*, 715-721, doi:10.1136/ard.2003.007039.
19. Tak, P.P.; Spaeny-Dekking, L.; Kraan, M.C.; Breedveld, F.C.; Froelich, C.J.; Hack, C.E. The levels of soluble granzyme A and B are elevated in plasma and synovial fluid of patients with rheumatoid arthritis (RA). *Clinical and experimental immunology* **1999**, *116*, 366-370.
20. Coon, T.A.; Glasser, J.R.; Mallampalli, R.K.; Chen, B.B. Novel E3 ligase component FBXL7 ubiquitinates and degrades Aurora A, causing mitotic arrest. *Cell cycle (Georgetown, Tex.)* **2012**, *11*, 721-729, doi:10.4161/cc.11.4.19171.
21. Croft, M.; So, T.; Duan, W.; Soroosh, P. The significance of OX40 and OX40L to T-cell biology and immune disease. *Immunological reviews* **2009**, *229*, 173-191, doi:10.1111/j.1600-065X.2009.00766.x.
22. Hong, J.T.; Son, D.J.; Lee, C.K.; Yoon, D.Y.; Lee, D.H.; Park, M.H. Interleukin 32, inflammation and cancer. *Pharmacology & therapeutics* **2017**, *174*, 127-137, doi:10.1016/j.pharmthera.2017.02.025.
23. Berg, R.E.; Forman, J. The role of CD8 T cells in innate immunity and in antigen non-specific protection. *Current opinion in immunology* **2006**, *18*, 338-343, doi:10.1016/j.coi.2006.03.010.

Figure S1. Principal component analysis of two batch effects removed data sets. (a) Set A (GSE1919, GSE39340, and GSE36700), (b) Set B (GSE55457, GSE55584, and GSE55235)

a

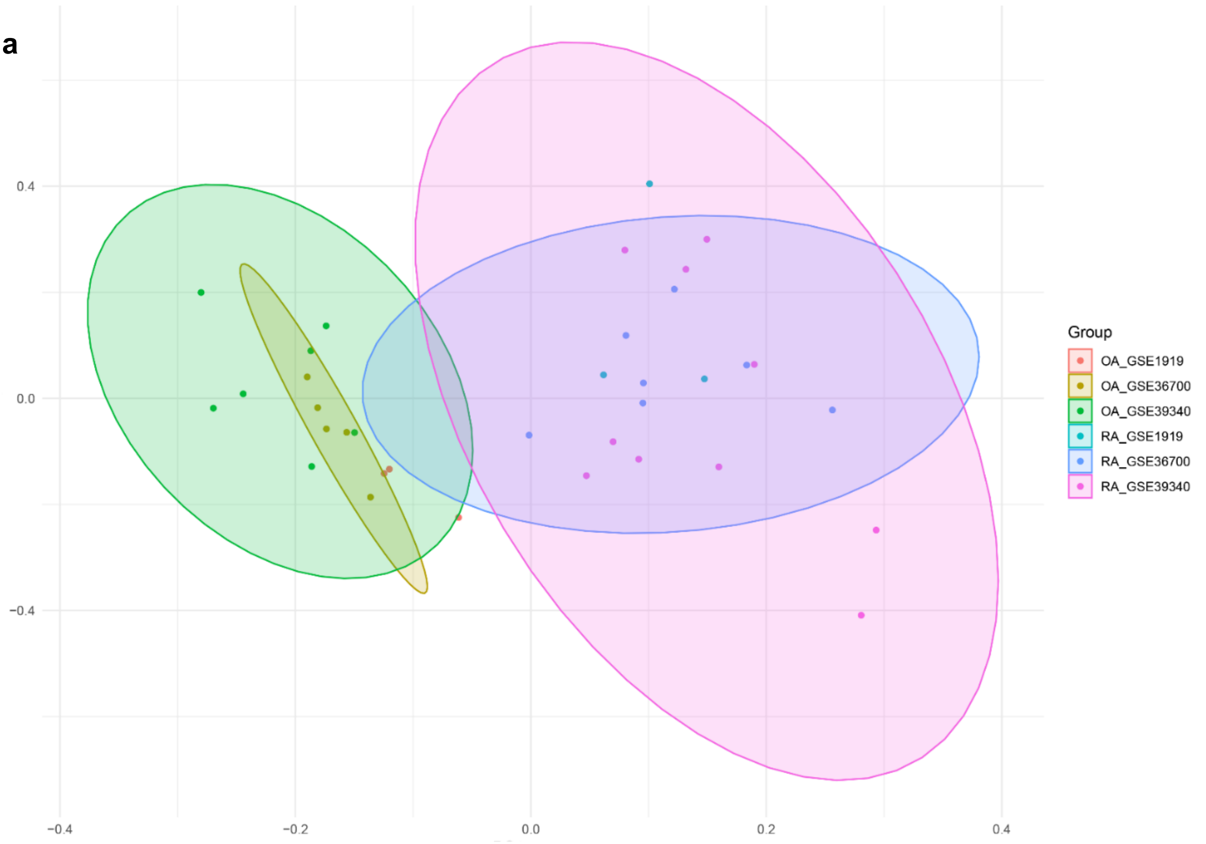

b

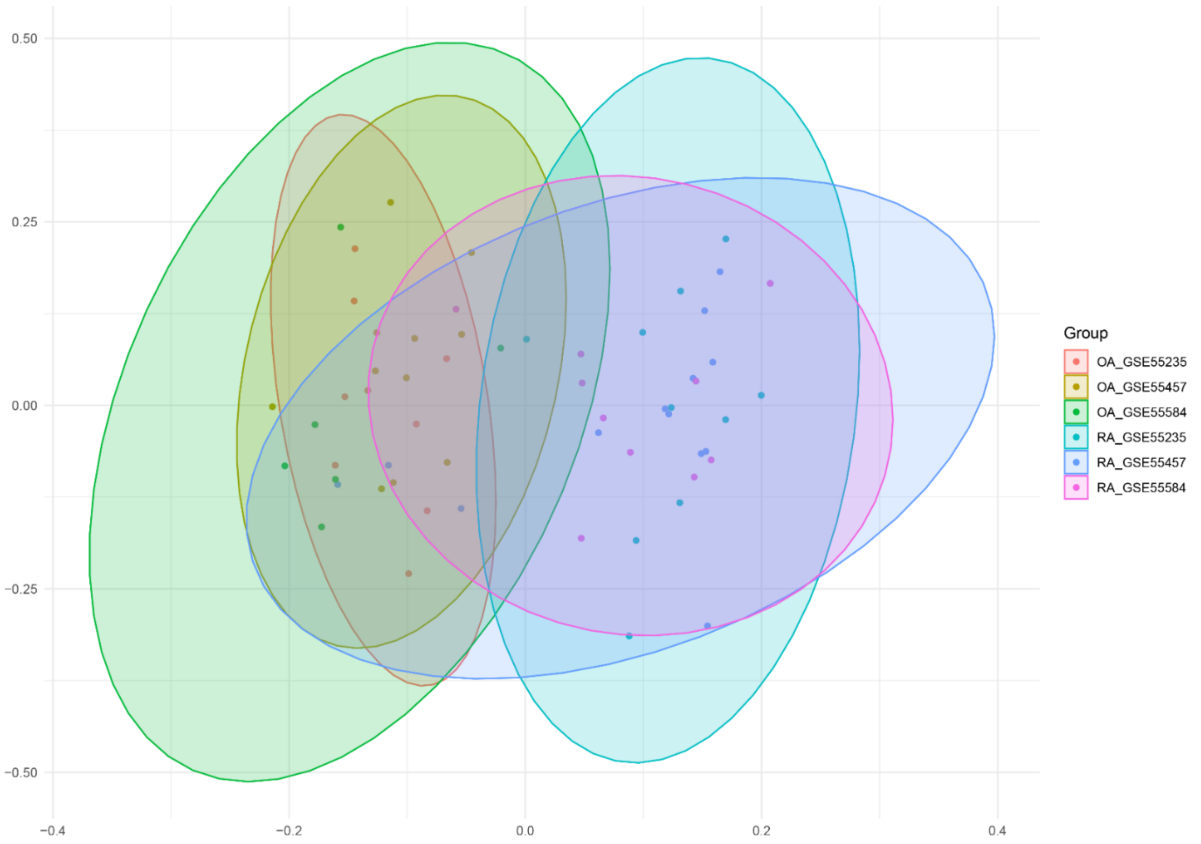

Figure S2. The STRING protein-protein interaction network of differentially expressed genes. (a) Up-regulated network. (b) Down-regulated network. According to STRING, red, green, blue, purple, yellow, light blue, and black strings demonstrated the presence of fusion, neighborhood, co-occurrence, experimental, text mining, database, and co-expression evidence, respectively.

a

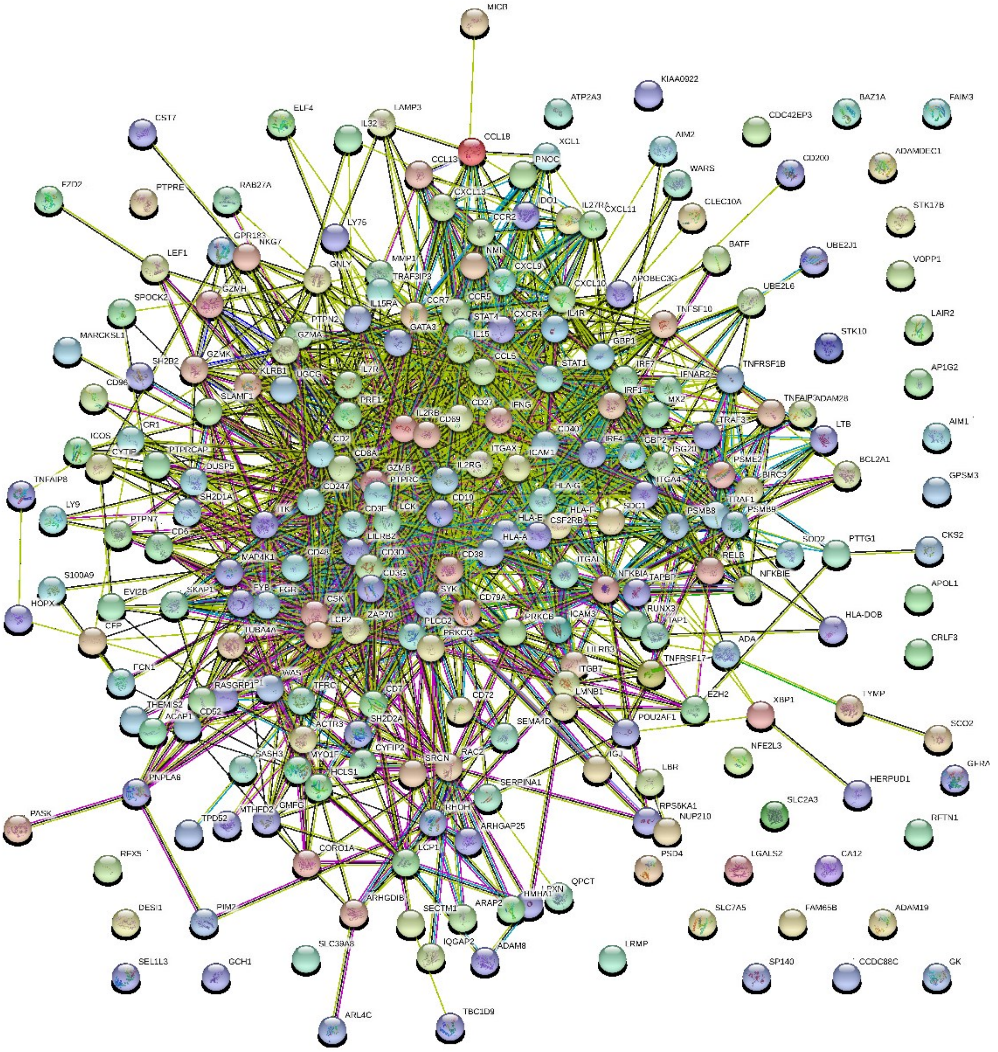

b

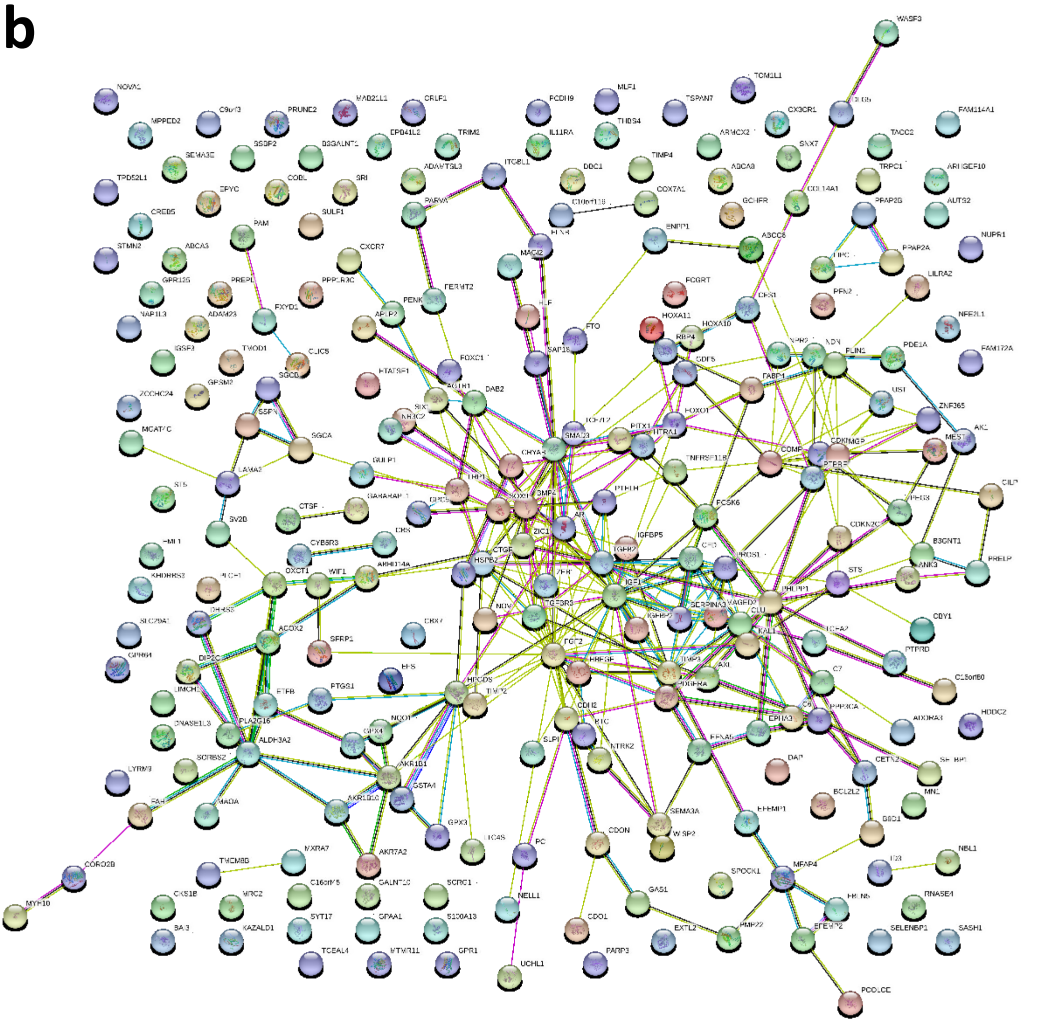

Data S1. Descriptive analysis and visualization of 16 predictors in RA versus OA

Summary statistic of 16 genes in the signature.

Exploratory data analysis

17 Variables 59 Observations

Class

n	missing	distinct
59	0	2

Value	OA	RA
Frequency	26	33
Proportion	0.441	0.559

TMOD1

n	missing	distinct	Info	Mean	Gmd	.05	.10	.25	.50	.75	.90	.95
59	0	59	1	8.846	0.6968	7.818	8.109	8.437	8.829	9.231	9.638	10.002

lowest : 7.610727 7.745064 7.772906 7.822517 7.958150, highest: 9.849471 10.000442 10.016928 10.065825 10.090771

POP7

n	missing	distinct	Info	Mean	Gmd	.05	.10	.25	.50	.75	.90	.95
59	0	58	1	8.717	0.2745	8.352	8.393	8.568	8.715	8.855	9.024	9.095

lowest : 8.106792 8.266364 8.266836 8.361928 8.376697, highest: 9.070290 9.084416 9.192743 9.257256 9.316017

SGCA

n	missing	distinct	Info	Mean	Gmd	.05	.10	.25	.50	.75	.90	.95
59	0	59	1	7.181	0.6291	6.516	6.611	6.723	7.059	7.648	7.940	8.217

lowest : 6.183825 6.482914 6.485094 6.519905 6.539315, highest: 8.059750 8.215069 8.229736 8.309589 8.317227

KLRD1

n	missing	distinct	Info	Mean	Gmd	.05	.10	.25	.50	.75	.90	.95
59	0	59	1	7.089	0.2206	6.836	6.889	6.963	7.023	7.196	7.419	7.518

lowest : 6.735841 6.810775 6.833975 6.836329 6.854272, highest: 7.468002 7.517152 7.529966 7.569629 7.593283

ALOX5

n	missing	distinct	Info	Mean	Gmd	.05	.10	.25	.50	.75	.90	.95
59	0	59	1	8.197	0.441	7.626	7.659	7.871	8.165	8.488	8.671	8.848

lowest : 7.570915 7.575339 7.592925 7.629234 7.644564, highest: 8.729255 8.847581 8.847684 8.946761 8.991679

RAB22A

n	missing	distinct	Info	Mean	Gmd	.05	.10	.25	.50	.75	.90	.95
59	0	59	1	8.857	0.2703	8.470	8.497	8.720	8.851	9.013	9.155	9.242

lowest : 8.272843 8.324045 8.468389 8.469915 8.474224, highest: 9.200658 9.238832 9.266402 9.306271 9.323720

ANK3

n	missing	distinct	Info	Mean	Gmd	.05	.10	.25	.50	.75	.90	.95
59	0	59	1	8.353	0.8178	7.264	7.448	7.794	8.398	8.742	9.325	9.600

lowest : 6.898430 7.048985 7.206742 7.269947 7.314950, highest: 9.482349 9.599703 9.605982 9.788882 9.860875

PTPN3

n	missing	distinct	Info	Mean	Gmd	.05	.10	.25	.50	.75	.90	.95
59	0	59	1	7.785	0.2498	7.412	7.521	7.635	7.811	7.930	8.036	8.059

lowest : 7.339715 7.391992 7.407612 7.411971 7.441290, highest: 8.043195 8.051574 8.123203 8.158928 8.312575

GZMK

n	missing	distinct	Info	Mean	Gmd	.05	.10	.25	.50	.75	.90	.95
59	0	59	1	9.463	1.067	8.221	8.437	8.713	9.243	10.194	10.651	11.193

lowest : 7.590958 8.073029 8.132376 8.230423 8.263204, highest: 11.012626 11.172825 11.376806 11.466007 11.581469

CLU

n	missing	distinct	Info	Mean	Gmd	.05	.10	.25	.50	.75	.90	.95
59	0	59	1	12.33	1.068	10.33	10.92	11.85	12.46	13.03	13.42	13.49

lowest : 9.736136 10.071174 10.329075 10.335610 10.771648, highest: 13.473839 13.483270 13.504305 13.534891 13.687239

GZMB

n	missing	distinct	Info	Mean	Gmd	.05	.10	.25	.50	.75	.90	.95
59	0	59	1	7.041	0.9236	6.082	6.209	6.323	6.847	7.649	8.122	8.671

lowest : 5.675563 5.937515 6.031668 6.087965 6.090291, highest: 8.598172 8.671046 8.672235 8.687011 8.729226

FBXL7

n	missing	distinct	Info	Mean	Gmd	.05	.10	.25	.50	.75	.90	.95
59	0	59	1	9.112	0.3671	8.654	8.722	8.880	9.050	9.260	9.511	9.663

lowest : 8.508760 8.631044 8.640953 8.655982 8.664510, highest: 9.556073 9.634681 9.919319 9.926876 10.121697

TNFRSF4

n	missing	distinct	Info	Mean	Gmd	.05	.10	.25	.50	.75	.90	.95
59	0	59	1	7.076	0.1839	6.833	6.881	6.961	7.067	7.149	7.276	7.383

lowest : 6.764937 6.789501 6.798118 6.836650 6.840137, highest: 7.304520 7.381716 7.391577 7.424735 7.560860

IL32

n	missing	distinct	Info	Mean	Gmd	.05	.10	.25	.50	.75	.90	.95
59	0	59	1	9.007	0.9259	8.023	8.110	8.346	8.823	9.654	10.153	10.289

lowest : 7.252458 7.663641 8.010068 8.024958 8.040544, highest: 10.242136 10.284808 10.326930 10.397811 11.140009

MXRA7

n	missing	distinct	Info	Mean	Gmd	.05	.10	.25	.50	.75	.90	.95
59	0	59	1	12.09	0.4233	11.52	11.60	11.75	12.12	12.38	12.54	12.59

lowest : 11.42489 11.47054 11.50686 11.51939 11.54377, highest: 12.56083 12.56786 12.75576 12.76353 12.90759

CD8A

n	missing	distinct	Info	Mean	Gmd	.05	.10	.25	.50	.75	.90	.95
59	0	59	1	8.857	0.6964	7.985	8.134	8.348	8.762	9.251	9.615	9.967

lowest : 7.897155 7.952875 7.959216 7.988354 8.116281, highest: 9.922451 9.944335 10.169961 10.261575 10.328671

Note: Descriptive analysis was conducted using Hmisc package in R

Violin Plots of the expression levels of 16 genes in the signature.

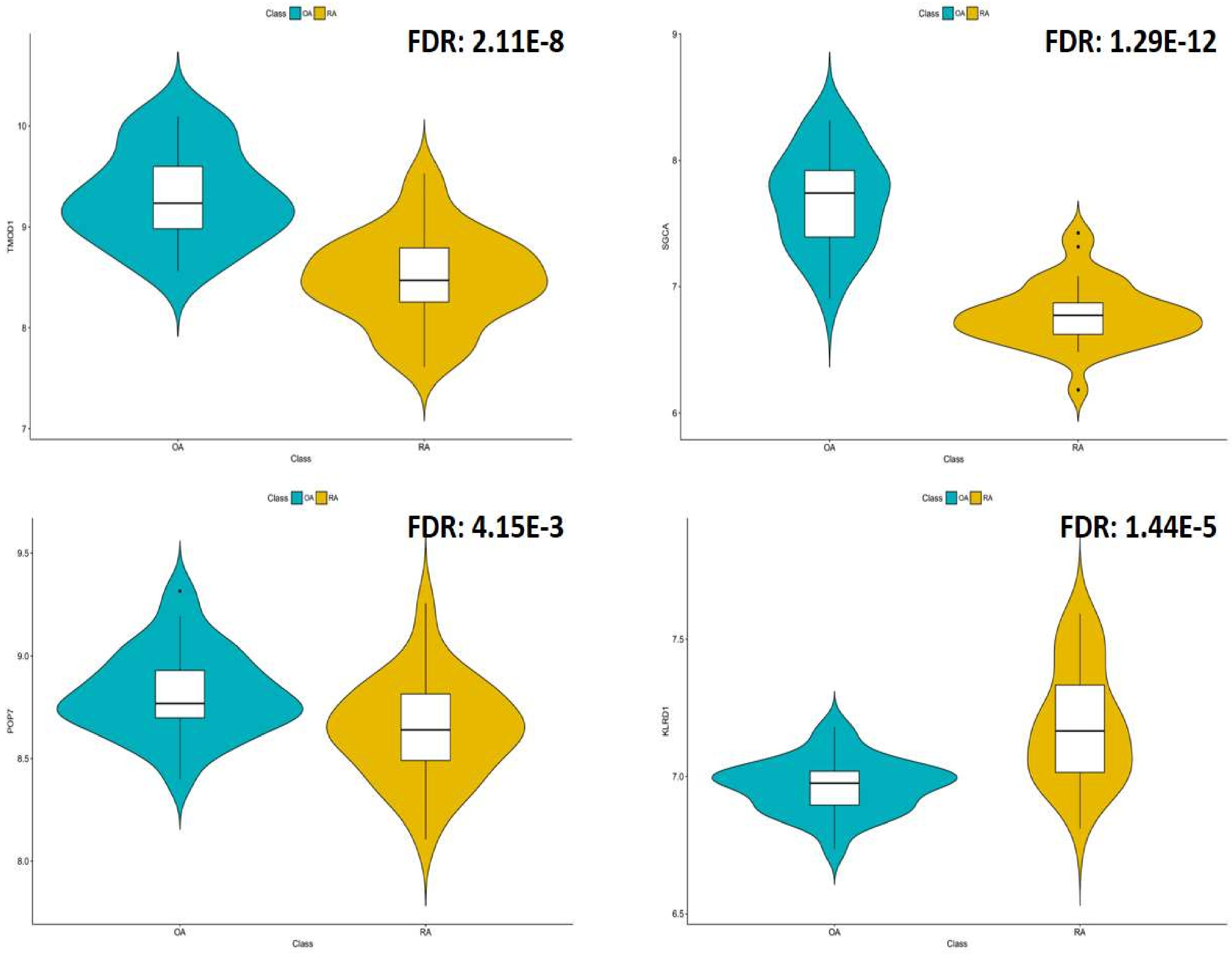
Class OA RA
FDR: 2.11E-8
TMOD1
10
9
8
7
OA
RA
Class
Class OA RA
FDR: 1.29E-12
SGCA
9
8
7
6
OA
RA
Class
Class OA RA
FDR: 4.15E-3
POP7
9.5
9.0
8.5
8.0
OA
RA
Class
Class OA RA
FDR: 1.44E-5
KLRD1
7.5
7.0
6.5
OA
RA
Class

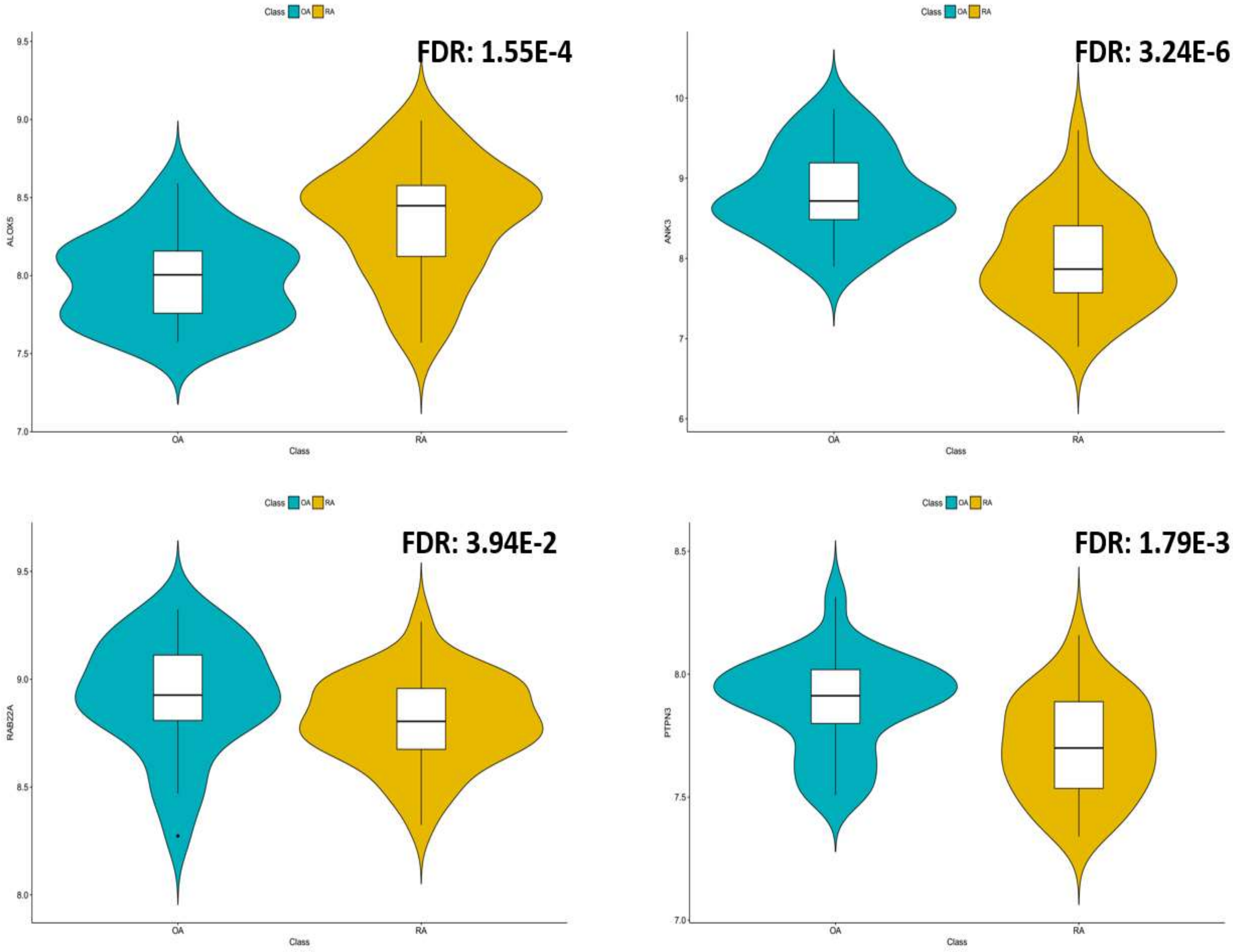
Class OA RA
FDR: 1.55E-4
ALOX5
9.5
9.0
8.5
8.0
7.5
7.0
OA
RA
Class
Class OA RA
FDR: 3.24E-6
ANK3
10
9
8
7
6
OA
RA
Class
Class OA RA
FDR: 3.94E-2
RAB22A
9.5
9.0
8.5
8.0
OA
RA
Class
Class OA RA
FDR: 1.79E-3
PTPN3
8.5
8.0
7.5
7.0
OA
RA
Class

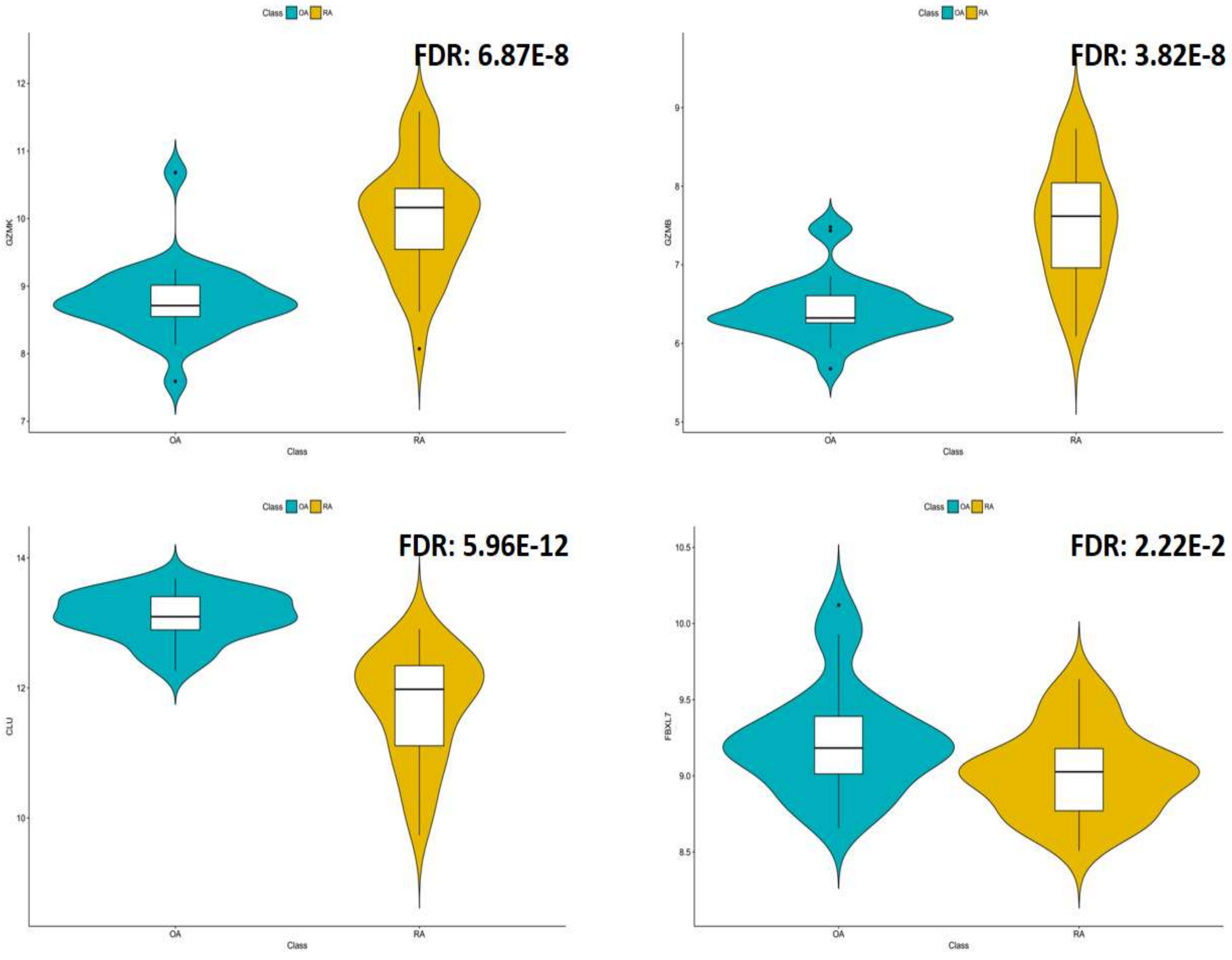
Class OA RA
FDR: 6.87E-8
GZMK
12
11
10
9
8
7
OA
RA
Class
Class OA RA
FDR: 3.82E-8
GZMB
9
8
7
6
5
OA
RA
Class
Class OA RA
FDR: 5.96E-12
CLU
14
12
10
OA
RA
Class
Class OA RA
FDR: 2.22E-2
FBXL7
10.5
10.0
9.5
9.0
8.5
OA
RA
Class

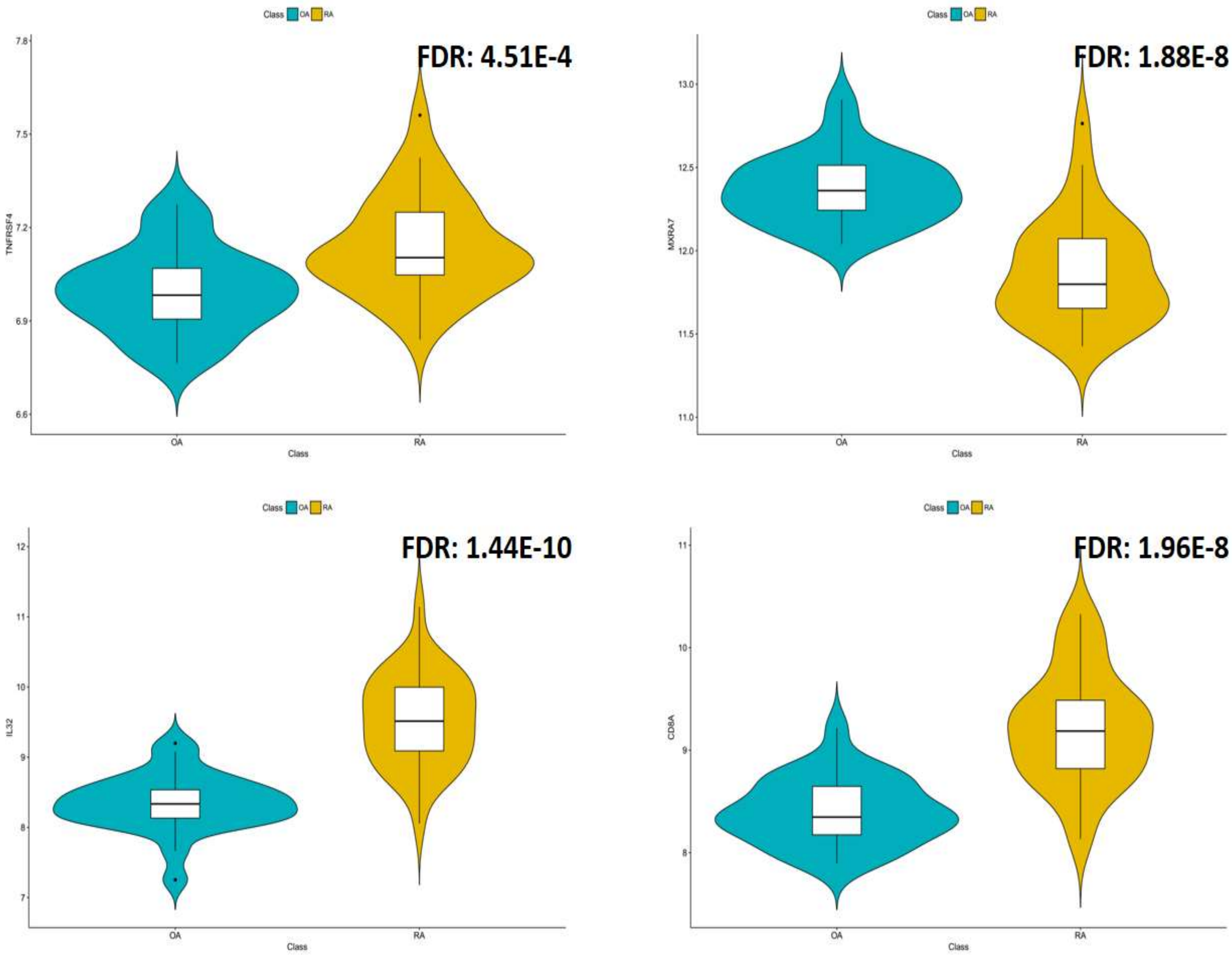
Class OA RA
FDR: 4.51E-4
TNFRSF4
7.8
7.5
7.2
6.9
6.6
OA
RA
Class
Class OA RA
FDR: 1.88E-8
MXRA7
13.0
12.5
12.0
11.5
11.0
OA
RA
Class
Class OA RA
FDR: 1.44E-10
IL32
12
11
10
9
8
7
OA
RA
Class
Class OA RA
FDR: 1.96E-8
CD8A
11
10
9
8
OA
RA
Class
